# Microbiome and aging: Trajectories of microbiome age across human ecosystems and their systemic effects

**DOI:** 10.1002/imt2.70148

**Published:** 2026-07-06

**Authors:** Zhexin Ni, Jiaojiao Xie, Zhichun Zhang, Keming Zhang, Jie Ding, Mingyang Chang, Ying Su, Yunan Zhang, Mingfei Yao, Chengyuan Wang, Yan Yan, Wenjie Fang, Hainv Gao, Xuecong Tian, Yangshuo Li, Xinlin Zhu, Wen Zhang, Wanqing Liao, Gaoyuan Xu, Guojing Chen, Xiaoyi Lv, Chaoqin Yu, Weihua Pan, Vadim Molodtsov, Lanjuan Li, Yue Gao, Wei Zhou

**Affiliations:** ^1^ Academy of Military Medical Sciences Beijing China; ^2^ State Key Laboratory for Diagnosis and Treatment of Infectious Diseases, National Clinical Research Center for Infectious Diseases, China‐Singapore Belt and Road Joint Laboratory on Infection Research and Drug Development, National Medical Center for Infectious Diseases, Collaborative Innovation Center for Diagnosis and Treatment of Infectious Diseases, The First Affiliated Hospital Zhejiang University School of Medicine Hangzhou China; ^3^ Department of Cariology and Endodontology Peking University School and Hospital of Stomatology Beijing China; ^4^ Department of Dermatology The Second Affiliated Hospital of Naval Medical University Shanghai China; ^5^ Gynecology Department of Traditional Chinese Medicine The First Affiliated Hospital of Naval Medical University Shanghai China; ^6^ Institute of Traditional Chinese Medicine Tianjin University of Traditional Chinese Medicine Tianjin China; ^7^ School of Computer Science and Technology Xinjiang University Urumqi China; ^8^ School of Life Sciences Beijing University of Chinese Medicine Beijing China; ^9^ Yuhang Institute for Collaborative Innovation and Translational Research in Life Sciences and Technology Hangzhou China; ^10^ Shanghai Institute of Materia Medica Chinese Academy of Sciences Shanghai China; ^11^ Department of Infectious Diseases, Key Laboratory of Artificial Organs and Computational Medicine of Zhejiang Province, Shulan (Hangzhou) Hospital, Shulan International Medical College, Zhejiang Shuren University Hangzhou China; ^12^ Ghent University Ghent Belgium; ^13^ Florida Atlantic University Boca Raton Florida USA; ^14^ China Medical University Shenyang China; ^15^ College of Software, Xinjiang University Urumqi China; ^16^ Peoples' Friendship University of Russia Moscow Russian Federation; ^17^ Xinjiang Key Laboratory of High‐Altitude Diseases and Characteristic Preparations Urumqi China

**Keywords:** aging, cross‐organ homeostasis, machine learning, microbiome age, multisite microbiome, systems biology

## Abstract

The “microbiome age,” a computationally derived systemic biosignature, is emerging as a pivotal framework for deciphering host aging trajectories and multi‐organ health status. Beyond merely cataloging taxonomic shifts within specific niches, this concept integrates the cumulative biological effects of microbial community structure, functional homeostasis, ecological interactions, and host regulatory dynamics over time. Accumulating evidence indicates that a deviation between chronological and microbiome age‐termed the “microbiome age gap”‐closely correlates with systemic declines in immunomodulation, metabolic robustness, barrier integrity, and neuroendocrine regulation, positioning it as a potent predictor of healthspan and disease risk. A defining feature of microbial aging is the convergence of divergent ecological niches toward a state of dysbiosis. Despite distinct compositional profiles across the gut, oral cavity, skin, and urogenital tract, these ecosystems commonly exhibit diminished stability, functional remodeling, and altered host–microbe interaction modes with advancing age. Concurrently, site‐specific signatures persist, linking microbial shifts to distinct physiological dimensions such as metabolic‐inflammatory axes, barrier function, and local hormonal environments. In this review, we systematically delineate the theoretical underpinnings and computational strategies for modeling microbiome age. We synthesize current evidence regarding age‐related microbial trajectories across diverse body habitats and propose an integrative framework: microbiome age serves dually as a holistic indicator of systemic aging and a sensitive window into localized organ vulnerability. This perspective not only advances our understanding of host‐microbe interactions in aging but also opens new avenues for precision stratification, personalized intervention, and healthspan management.

## Highlights

- Microbiome age is established as a computational biosignature unifying multi‐organ aging trajectories.
- Convergent aging trajectories are identified across gut, oral, skin, and reproductive microbiomes.
- Directional microbiome shifts mirror systemic declines in immunity, metabolism, and barrier function.
- Microbiome age serves as a dual biomarker predicting systemic risks and local vulnerabilities, enabling precision health assessment.

## Graphical abstract

Microbiome age (MA) has emerged as an innovative biomarker of biological aging, reflecting host aging trajectories through dynamic alterations in microbial composition, function, and host–microbe interactions across multiple body ecosystems. Diverse computational strategies—including traditional machine learning, deep learning, and multi‐omics longitudinal integration—enable the construction of microbiome age models from heterogeneous biological data. Across major human microbiota, including the gut, oral, skin, and reproductive, age‐associated microbial remodeling exhibits both site‐specific characteristics and convergent systemic patterns. Integration of these ecosystem‐specific microbiome ages (X‐MA) provides a multidimensional framework for evaluating organismal aging status. Mechanistically, microbiome aging is closely linked to inflammaging and systemic physiological decline through chronic low‐grade inflammation, epithelial barrier dysfunction, metabolic dysregulation, immune imbalance, neuroendocrine disruption, oxidative stress, and cellular senescence. Together, MA represents a promising systems‐level biomarker bridging computational prediction, microbial ecology, and aging biology, with broad implications for aging assessment, risk stratification, and precision intervention.

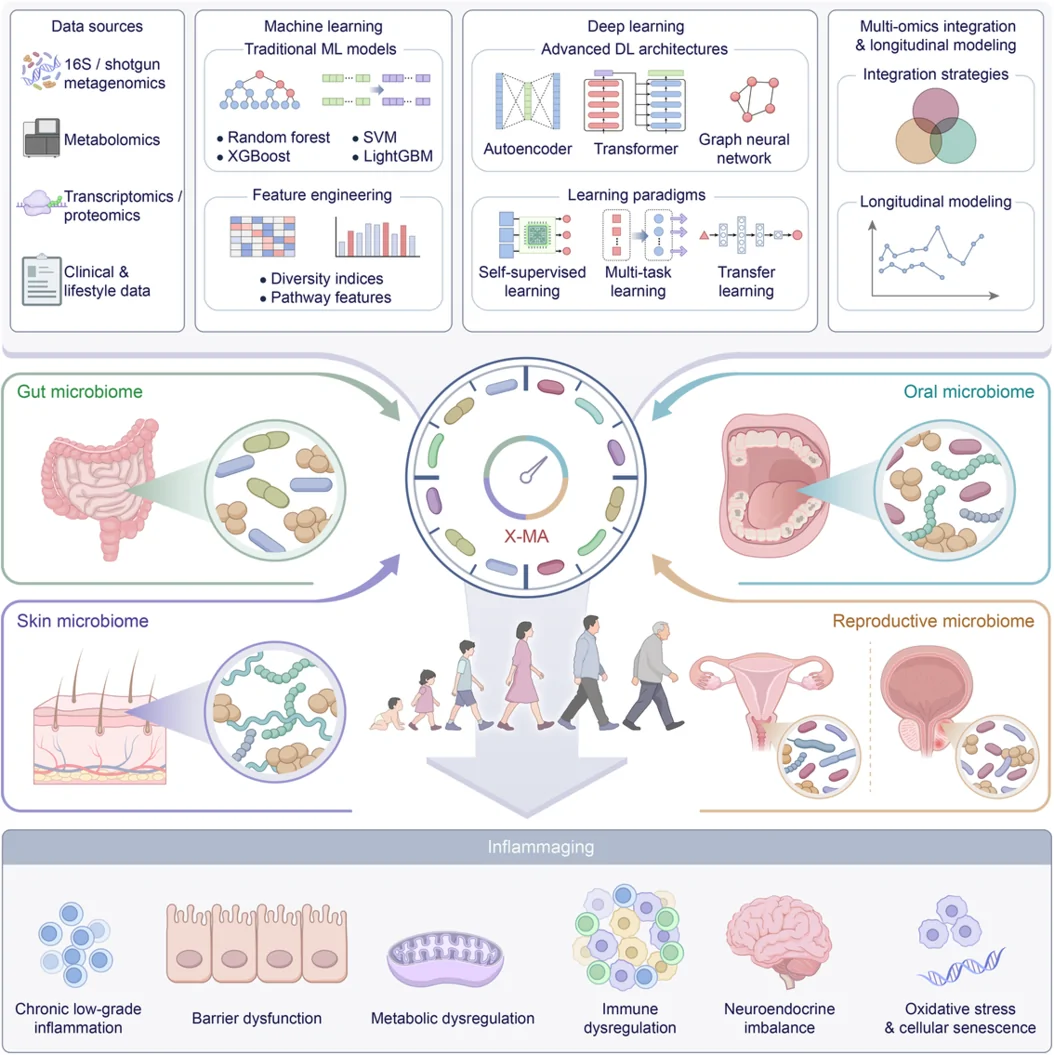

## INTRODUCTION

Aging is a complex, progressive, and multisystemic process characterized by the gradual loss of physiological integrity, leading to increased vulnerability and disease susceptibility. While classical and modern theories—ranging from genomic instability to stem cell exhaustion—have established a multidimensional framework for understanding aging mechanisms [1], the rise of microbiome science has positioned host‐associated microbial communities as central players in this process. The 2023 update to the “Hallmarks of Aging” officially recognized “dysbiosis” alongside “inflammaging” as a core hallmark, underscoring the theoretical importance of microbe‐host interactions in systemic aging [1]. Consequently, quantifying the dynamic relationship between microbial ecology and host aging has become a critical scientific challenge linking micro‐ecological shifts to macro‐scale phenotypes.

By adopting the quantitative perspective of microbiome age (MA), this review synthesizes the dynamic rules governing multi‐site microbial ecosystems. We aim to elucidate how local ecological shifts drive systemic aging through mechanisms like inflammaging, thereby deepening our understanding of aging complexity and laying a theoretical foundation for microbiome‐based risk assessment, early warning systems, and targeted interventions.

## MA: ASSESSMENT METHODOLOGIES AND BIOLOGICAL IMPLICATIONS

The proliferation of high‐throughput sequencing has generated high‐dimensional microbial datasets, necessitating a shift from descriptive statistics to predictive modeling. The integration of machine learning—particularly ensemble learning and deep learning frameworks—has enabled the construction of robust age prediction models. These algorithms operate within high‐dimensional feature spaces to condense complex taxonomic profiles, functional gene repertoires, and metabolic characteristics into a single, continuous aging readout. This transition marks a paradigm shift from mere description of microbial changes to the quantitative prediction of host aging status.

**Figure 1:**
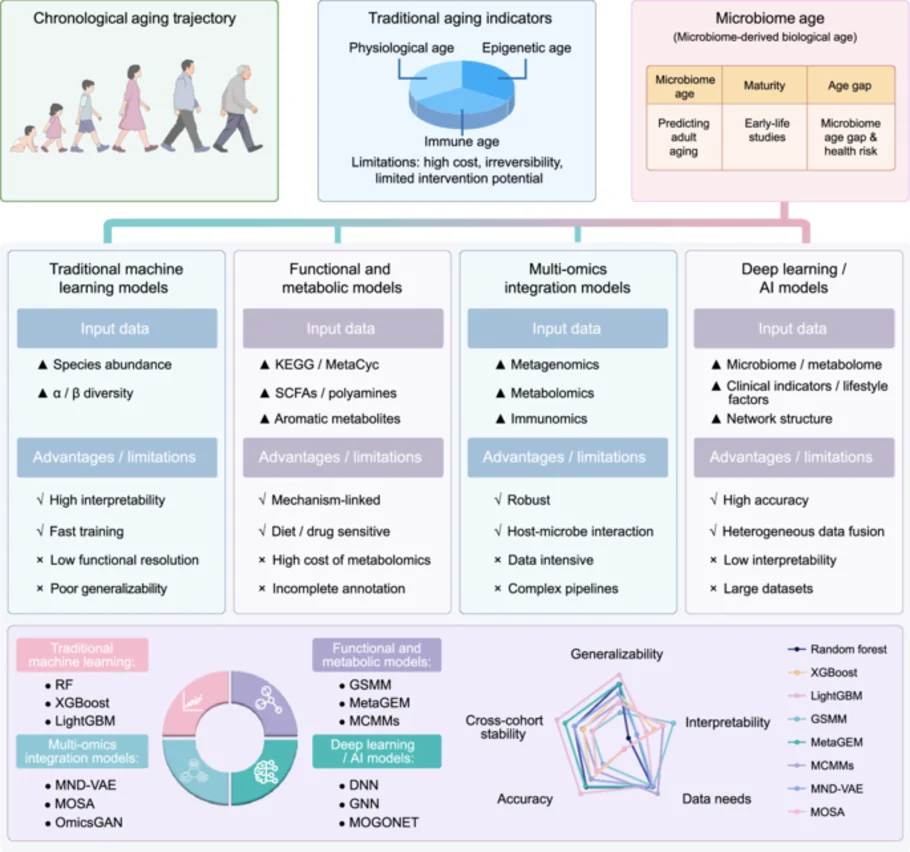
Conceptual framework and computational approaches for microbiome age. Traditional aging biomarkers, including physiological, epigenetic, and immune age metrics, have contributed substantially to biological aging assessment but are often constrained by limited accessibility, high cost, poor dynamic responsiveness, or restricted intervention relevance. In contrast, microbiome age (MA), defined as a microbiome‐derived indicator of biological aging based on compositional and functional microbial features, has emerged as a promising and dynamically responsive biomarker reflecting host aging trajectories. Related concepts include microbiome maturity, primarily applied to infant and early‐life developmental studies, and microbiome age gap, representing the deviation between predicted microbiome age and chronological age, which may indicate altered health status or disease risk. Current computational strategies for MA prediction can be broadly categorized into four major classes: (1) traditional machine learning models, which provide rapid training and strong interpretability but limited functional sensitivity and cross‐cohort generalizability; (2) functional and metabolic models, which offer improved biological interpretability by incorporating metabolic pathways and microbial metabolites, yet remain limited by incomplete functional annotations and the high cost of metabolomics profiling; (3) multi‐omics integration models, which enhance predictive robustness and capture host–microbiome interactions, although they require complex analytical workflows and substantial data resources; and (4) deep learning and artificial intelligence models, which achieve superior predictive performance and accommodate heterogeneous high‐dimensional data, but often suffer from reduced interpretability and dependence on large‐scale training datasets. Together, these complementary computational frameworks define the current methodological landscape of microbiome age modeling and provide a foundation for its future application in precision aging research and clinical translation.

Consequently, MA is not merely a biological marker but an algorithm‐derived phenotype (ADP). Its core logic relies on supervised learning to establish a mapping function between host age and microbial features; the deviation between predicted and chronological age—the microbiome age gap (MAG)—serves as a quantitative proxy for individual aging trajectories. The maturation of these algorithmic frameworks, coupled with enhanced capabilities for multi‐modal data integration, has driven the conceptual evolution from observing “microbial shifts” to defining MA; the progression establishes a rigorous methodological foundation for subsequent functional dissection and precision interventions (Figure 1).

### Conceptual evolution: From “microbial shifts” to MA

#### The rationale behind the emergence of MA

Aging assessment is a cornerstone of gerontology and public health. While conventional aging metrics have been established, they exhibit significant limitations [1]. Chronological age often fails to capture biological heterogeneity [2], epigenetic clocks are constrained by high costs and technical complexity, limiting large‐scale population screening [3], and immune aging scores lack the robustness required for long‐term dynamic monitoring [4]. These gaps underscore the urgent need for scalable, cost‐effective, and dynamic biomarkers to facilitate personalized health management and interventional studies.

The human microbiome—encompassing the gut, skin, oral, and reproductive ecosystems—offers a transformative solution. Unlike static genetic blueprints, microbial communities are inherently modifiable and reversible, presenting a unique “anchor point” for aging research. Specifically, the gut microbiome can be quantitatively profiled via sequencing and is responsive to interventions targeting age‐related decline [5]. The skin microbiome, integral to barrier function and immunity, exhibits distinct aging signatures amenable to topical modulation. The oral microbiome provides a non‐invasive, accessible sampling site with strong predictive power for systemic health [6]. This convergence of quantifiability, modifiability, and reversibility positions the microbiome as a superior dynamic biosensor for aging.

Consequently, this review defines MA as a unifying conceptual framework describing age‐related microbial features across the host. However, it is crucial to distinguish between the conceptual framework and the current empirical evidence: current data primarily support site‐specific MA (e.g., gut‐MA, skin‐MA, oral‐MA, reproductive‐MA). These are constructed based on the unique taxonomic, functional, and metabolic profiles of distinct ecological niches and are not yet interchangeable as a universal “microbial clock” [7]. Furthermore, the rapid advancement of multi‐omics technologies has become a mainstream trend in life sciences, enhancing the characterization of biological age through the integration of multidimensional data [8]. As a critical component of this multi‐omics ecosystem, the microbiome propels aging research from a reductionist, single‐biomarker approach toward a systemic, multi‐site, and integrated paradigm [7].

#### Core definition of MA

The concept of MA was first proposed by Galkin et al. and subsequently refined and validated by multiple independent consortia. It is defined as a quantitative biosignature derived from standardized computational models that integrate the taxonomic composition, functional gene repertoires, and metabolic profiles of microbial communities across various body habitats (e.g., gut, skin, oral, and reproductive tracts). MA serves as a proxy for the host's biological aging status, capturing age‐related deviations in microbial ecology [6, 9, 10].

It is critical to distinguish MA from “microbiome maturity,” a term traditionally used to describe the developmental trajectory of microbial communities from infancy to early adulthood. In contrast, MA spans the entire human lifespan, focusing specifically on the dynamic transition from maturity to senescence. This allows for the continuous monitoring of aging progression [10]. Currently, the gut‐MA represents the most mature and robust iteration of this concept. In contrast, models for the skin, oral, and reproductive tracts are at varying stages of development. Crucially, these site‐specific clocks (X‐MA) reflect distinct biological dimensions: Gut‐MA primarily mirrors systemic metabolic and immune senescence, whereas skin, oral, and reproductive MA predominantly index the aging of peripheral barriers, systemic inflammation, and reproductive endocrinology, respectively [6].

A key derivative metric is the MAG, defined as the residual difference between the predicted X‐MA and the host's chronological age. Expanding evidence suggests that MAG quantifies the deviation from a healthy reference trajectory. A negative MAG indicates an “accelerated” microbial phenotype relative to peers, often correlating with localized functional decline or elevated disease risk [6]. However, whether MAG represents systemic aging acceleration remains to be validated through multi‐site integration and phenotypic correlation [11, 12]. Additionally, microbial richness serves as a complementary indicator; a consistent inverse correlation between reduced α diversity and increased MA has been observed across multiple niches, positioning richness as a universal reference for microbial senescence [13].

#### Systems biology implications of MA

From a systems biology perspective, MA should not be viewed as a uniform readout from a single source. Instead, it is more accurately conceptualized as a set of partitioned “ecological readouts” arising from the interactions between specific niches and distinct host physiological systems [14]. For instance, the gut microbiome is intrinsically linked to metabolic and inflammatory homeostasis, while the skin microbiome reflects barrier function and exposome exposure. The oral microbiome interfaces with mucosal immunity and periodontal health, and the reproductive microbiome is predominantly governed by endocrine fluctuations [15, 16]. Crucially, these microbial dynamics are reciprocally shaped by the host's physiological state, which alters the niche landscape [16]. Therefore, X‐MAs are best understood as parallel, non‐interchangeable indicators reflecting distinct dimensions of host health, rather than components of a single, unified biological clock.

MA constitutes a critical pillar of the multi‐dimensional biological age assessment framework, complementing epigenetic clocks and immune age. Epigenetic age captures aging imprints at the genomic regulatory level [17], while immune age quantifies senescence within the immune system's cellular diversity and functional efficiency [18]. In contrast, MA specifically reports on the functional decline of microbe‐host interactions, particularly in the realms of metabolic and immune regulation mediated by the microbiota [19]. Together, these three axes provide a comprehensive, multi‐perspective map of the aging process, elucidating mechanisms that are invisible to any single metric alone.

#### Heterogeneity and asynchrony across body habitats

Accumulating evidence demonstrates that microbial aging is not a synchronous process; instead, it exhibits profound site‐specific heterogeneity (Table 1). Therefore, MA is more accurately defined as a site‐specific MA composed of distinct X‐MAs rather than a singular index.

**Table 1:** Heterogeneity and comparison of site‐specific microbiome age (X‐MA) across diverse ecological niches.

Dimension	Gut	Skin	Oral	Reproductive	Reference support
Modeling of the microbial aging clock	Maturity	Better	Preliminary	None	[20, 21]
Main driving factors	Inflammation‐metabolism‐immune axis	Environmental exposure, Decline of barriers	Oral hygiene, periodontal condition, cognitive function	Estrogen changes, menopause	[6, 21, 22, 23, 24]
Aging pattern	Progressive continuous change	Progressive continuous change	Progressive continuous change	Periodic jumps	[6, 10, 23, 24, 25]
The direction of changes in microbial community diversity with age	Tending towards individualization	Increase in α‐diversity	Correlated positively with age and negatively with frailty	Increased diversity after menopause	[6, 10, 21, 22, 24]
The main associated host aging axis	SCFAs, LPS leakage, inflammatory pathways	Pathogen reservoir, AMR transmission, barrier function	SCFAs‐producing bacteria, periodontitis, brain‐mouth axis	Estrogen‐estrobolome axis	[6, 10, 21, 22, 24, 25]

Abbreviations: LPS, lipopolysaccharide; SCFAs, short‐chain fatty acids.

First, significant disparities exist in predictive fidelity. Comparative studies indicate that skin microbiome models often yield lower Mean Absolute Errors than oral or gut models, suggesting fundamental differences in the information architecture of age signals across niches [20, 21]. Second, the biological mechanisms driving these changes are distinct. Gut microbiota aging is linked to metabolic‐inflammatory axes, skin to barrier function and exposome, oral to mucosal immunity, and reproductive microbiota to hormonal fluctuations, often exhibiting stage‐like transitions rather than linear progression [6, 22, 25]. This mechanistic divergence argues against the interchangeability of X‐MAs. Third, longitudinal trajectories are frequently asynchronous within the same individual. The discordance between different X‐MAs is often more strongly associated with frailty, infection risk, or localized functional decline than with chronological time itself. This suggests that each X‐MA captures a unique facet of functional decay. Currently, the lack of large‐scale, multi‐site sampling within single cohorts, coupled with methodological variations across studies, limits the feasibility of direct cross‐site integration.

In summary, MA functions as a multi‐site, multi‐dimensional aging phenome. Future research must prioritize the simultaneous construction of multiple X‐MAs within unified cohorts to evaluate their correlation, complementarity, and ultimate value in holistic aging assessment.

#### X‐MA: Characterization of host aging and its deviation

The construction of host age prediction models based on microbial signatures has emerged as a prominent research avenue. While microbial communities across diverse body habitats harbor age‐associated information, the ontological meaning of this “age” varies significantly. Current evidence distinguishes between the capacity to predict chronological age and the biological implications of the deviation between predicted and actual age—the MAG. This gap is increasingly recognized not as a mere model residual but as a potential biosignature reflecting physiological status, disease risk, or healthy aging trajectories. Therefore, interpreting MA requires a dual focus on predictive fidelity and the biological significance of its deviation.

The gut microbiota represents the most mature model, where the gut‐MA gap correlates robustly with metabolic status, systemic inflammation, disease burden, and survival outcomes [10]. This suggests that the gut–MA gap primarily quantifies a deviation along the metabolic‐inflammatory axis rather than simple chronological misalignment. Similarly, oral and skin microbiomes exhibit stable predictive power. Their respective gaps serve as sensitive indicators of health status shifts [23, 24, 26]. For instance, the oral MAG reflects diverse disease states, while deviations in skin–MA are linked to barrier function decline and exposome accumulation, capturing localized barrier integrity and mucosal immunity [27].

In contrast, the reproductive microbiome follows a distinct logic. Its age‐related dynamics are predominantly driven by endocrine fluctuations, manifesting as stage‐like restructuring during the menopausal transition rather than a continuous linear gradient [28, 29]. Consequently, the reproductive‐MA functions more as a proxy for “reproductive age” or “endocrine age.” Direct comparison with the linear age models of other sites is inappropriate due to this fundamental difference in temporal dynamics.

In summary, while MA models are broadly applicable, the biological dimensions they capture are site‐specific: gut–MA reflects metabolic‐inflammation, skin and oral MA index barrier function and exposure, and reproductive MA signifies hormonally driven life stages. The MAG should not be dismissed as a technical error but interpreted within its specific ecological and physiological context.

### Assessment methodologies: From statistical models to multimodal AI

#### Traditional taxonomic models and biomarker discovery

Traditional linear models often fail to capture the high‐dimensional, sparse, and non‐linear interaction features inherent in microbiome data. Consequently, ensemble learning algorithms based on decision trees have emerged as the predominant approach for constructing “microbial clocks.” Random Forest (RF), a Bagging‐based ensemble algorithm [30], effectively mitigates overfitting in high‐dimensional microbial datasets and serves as the benchmark for evaluating new model performance. RF has been instrumental in defining the Microbiome Maturity Index [31] and establishing correlations between gut microbial aging patterns and host cardiovascular health and mortality risk [26]. Building on this, gradient boosting frameworks such as XGBoost and LightGBM optimize sparse awareness and memory efficiency, serving as ideal cornerstones for processing high‐dimensional microbial features [32, 33, 34, 35]. Machine learning models rely on the age‐dependent succession of specific microbial taxa, some of which are conserved across populations. *Bifidobacterium spp*. represents core biomarkers of early gut development, often serving as key negative predictors in pediatric models [36]. Conversely, studies by Biagi et al. highlight taxa enriched in centenarians—such as *Christensenellaceae* and *Akkermansia*—as hallmarks of healthy aging. *Christensenellaceae* exhibits high heritability and positive correlations with low BMI and metabolic health [12], while *Akkermansia muciniphila* maintains gut barrier integrity and metabolic homeostasis, serving as a positive indicator of metabolic age [37]. In contrast, microbiota like *Enterobacteriaceae* and *Clostridium* are weighted heavily to predict frailty or advanced age, signaling pathological aging [26]. Stable age‐dependent succession patterns are also observed in oral and skin microbiomes, forming the basis for site‐specific clocks [20]. However, it is crucial to note that existing models are predominantly derived from gut data. The training sample size, feature space, and biological assumptions for skin and oral models differ substantially. Therefore, the performance metrics of X‐MAs are not directly analogous, and the reliability of one model should not be extrapolated to represent a universal MA.

In summary, current taxonomic models face limitations. First is geographic and ethnic bias; models trained on Western populations exhibit significant error when applied interracially [38, 39]. Second is functional insensitivity; microbial “functional redundancy” and the neglect of functional genes in 16S profiling result in low sensitivity for predicting physiological decline [40, 41, 42], driving a paradigm shift toward functional resolution.

#### Functional and metabolic age prediction models

The phenomenon of “functional redundancy” challenges models relying solely on taxonomy [43], shifting the research focus from identifying “who is there” (16S sequencing) to revealing “what they do” (metagenomics). Metagenomics provides granular insights into gene functions, metabolic pathways, and strain‐level evolution, primarily leveraging the KEGG and MetaCyc databases [44, 45]. Evidence suggests that metabolic pathway conservation surpasses species‐level conservation in healthy populations, yet aging triggers a directional drift in these pathways. While healthy aging is characterized by the retention of carbohydrate degradation and short‐chain fatty acids (SCFAs) synthesis pathways [46], frailty is marked by a shift toward protein fermentation and the upregulation of lipopolysaccharide synthesis and tryptophan metabolism [47]. Sato et al. demonstrated that despite high taxonomic heterogeneity in centenarians, their microbiomes exhibit remarkable functional convergence; quantitative features of secondary bile acid (BA) metabolism gene clusters outperform species abundance as “functional age markers” [48, 49].

The metabolome serves as the terminal “functional readout” of microbe‐host interactions, encompassing four core classes of biomarkers: (1) SCFAs: butyrate is a primary energy source for the intestinal epithelium; its decline correlates with insulin resistance and cognitive decline [50, 51, 52]. (2) Polyamines: microbiota‐synthesized spermidine induces autophagy and clears damaged organelles, acting as a core “longevity molecule” [53, 54]. (3) Trimethylamine N‐oxide (TMAO): a key negative marker for vascular aging, causally associated with cardiovascular disease and all‐cause mortality [55, 56, 57]. (4) Aromatic Metabolites: age‐related reductions in tryptophan metabolites drive systemic low‐grade inflammation, a core driver of inflammaging [58]. Similarly, skin barrier‐associated metabolites and oral microbial metabolic balance serve as vital markers for their respective site‐specific functional ages [6]. The advent of the “Functional MA” marks a paradigm shift from “species census” to “functional assessment” [59]. Its core tenet is that a “young” microbiome is defined by its capacity to maintain metabolic homeostasis rather than by specific taxonomic labels, offering a more accurate reflection of host biological age. Cutting‐edge research now employs species‐function hybrid models to fuse multi‐dimensional signals, significantly enhancing predictive accuracy while balancing interpretability via explainable AI (XAI) techniques [26, 60, 61]. While functional modeling is most mature in the gut—leveraging SCFAs, BAs, and aromatic metabolites—skin and oral sites likely depend on barrier‐specific metabolites and local inflammatory signals. The reproductive tract, driven by hormonal status and life‐cycle events, may require an independently developed functional framework.

#### Multi‐omics integration models and representative findings

With the advancement of systems biology, single‐omics data are insufficient to decode the complexity of aging. As the host and symbionts constitute a “hologenome,” integrating metagenomics, metabolomics, and host immunology is essential to reconstruct the molecular interactome [62, 63, 64]. The microbial shifts often precede alterations in host metabolic markers, endowing MA with early‐warning capabilities for aging inflection points [65, 66]. Notably, microbial features constitute a significant weight in the inflammatory aging clock (iAge), confirming the core regulatory role of trans‐kingdom signaling in senescence rates [67]. Given that aging is a continuous dynamic process, cross‐sectional data are susceptible to individual heterogeneity. Linear Mixed‐effects Models address correlation structures and imbalances in longitudinal data [68], stripping background noise by integrating baseline individual differences with temporal homeostatic features, thereby outperforming static models [69, 70, 71]. Furthermore, pseudo‐time series analysis infers “aging trajectories” from cross‐sectional snapshots, identifying driver biomarkers at state‐transition critical points linked to early‐onset cognitive decline [72, 73, 74].

Beyond taxonomic shifts, the ecological topology and functional interaction networks of microbial communities are hallmarks of aging. Declining community stability and increased stochastic drift serve as early warning signals of ecological senescence [36, 75]. Next‐generation models are shifting from mere abundance analysis to deep integration of ecological topology. Loss of resilience (anti‐perturbation capacity) and high‐entropy community states are emerging as core topological biomarkers [75, 76, 77, 78, 79]. Combined with cross‐omics prediction algorithms, a full‐chain framework from gene sequences to functional phenotypes has been established [80, 81, 82]. For the skin–MA, multi‐omics is pivotal. Metagenomics reveals species composition and functional genes; for instance, a multicenter study utilizing 2737 species‐level MAGs constructed a Microbiome‐based Facial Aging Index [83]. Amplicon sequencing assesses diversity, such as the colonization preferences of *Staphylococcus* across body sites [84]. Integrating culture‐dependent techniques with metabolomics validates mechanisms—e.g., *Streptococcus* secretions improving skin elasticity via collagen and lipid synthesis genes [85]. Multi‐omics strategies correlate microbial data with host parameters (pH, barrier proteins), revealing regulatory roles of metabolic pathways (e.g., fructose‐1‐phosphate kinase K02770) in aging [86].

#### Deep learning and AI frameworks in the field

The explosion of high‐dimensional, sparse, and heterogeneous multi‐modal data has exposed bottlenecks in traditional machine learning. As Hernandez Medina et al. [87] proposed, deep learning reshapes the assessment paradigm through powerful feature extraction and end‐to‐end learning, uncovering deep non‐linear patterns missed by conventional models. To address the “weak signal” problem in sparse data, autoencoder‐based models denoise through latent representation reconstruction [88]. Attention mechanisms enhance sensitivity to low‐abundance, longevity‐associated microbiota [89]. Phylogenetically‐informed Convolutional Networks precisely identify structural drift [90], while Transformer architectures overcome sequence length limitations [91, 92]. Graph Neural Networks (GNNs) integrate phylogenetic topology to identify disintegrating ecological modules, providing mathematical tools for explainable “topological clocks” [92, 93]. Deep learning breaks data silos, enabling deep fusion of microbiome data with multi‐dimensional host phenotypes. Moving beyond traditional latent variable analysis [94], deep learning utilizes attention mechanisms and heterogeneous GNNs for flexible multi‐modal fusion, capturing the “dysbiosis‐metabolic disorder” coupling mechanism in aging [95, 96]. Furthermore, introducing biologically informed causal inference frameworks and knowledge graphs overcomes the “black box” limitation, significantly enhancing model interpretability and clinical translation potential [97, 98]. In AI‐driven precision medicine, Microbiome Digital Twin models integrate community succession rules with host metabolism to accurately recapitulate microbial trajectories and predict host phenotypes [99]. This facilitates a leap from “trial‐and‐error” to “precision design” in anti‐aging interventions [100].

### Standardization and reproducibility challenges

Despite superior performance on specific datasets, the translation of “microbial clocks” from lab to clinic faces a reproducibility crisis. Microbiome variability spans the entire workflow from sample collection to bioinformatics analysis [101]. Technical inconsistencies easily mask true biological signals, and systematic biases introduced during experiments often exert multiplicative effects that cannot be eliminated by simple linear corrections [102]. Integrating data across sequencing platforms requires correcting platform‐specific fingerprints, and the low overlap rates resulting from different denoising pipelines highlight the urgency of unified reference databases and standardized analytical pipelines [103, 104]. Moreover, low‐biomass niches (skin, oral, reproductive) are exacerbated by contamination, high host DNA backgrounds, extraction inefficiencies, and batch effects. Models may erroneously learn experimental artifacts rather than authentic aging signals [105]. Simultaneously, the high environmental sensitivity of microbiomes makes them susceptible to confounders (geography, diet, drugs). Existing models, mostly trained on Western industrialized populations, often fail in non‐Western cohorts [106], and dietary or pharmacological perturbations are frequently misclassified as aging signatures [76, 107, 108, 109, 110].

Establishing a unified pipeline covering “wet‐lab” QC, metadata standardization, and global multicenter integration is the core path forward. This requires breaking the Western‐centric baseline to build a multicenter reference system [111], standardizing analytical workflows via platforms like QIIME 2 [112], strictly adhering to MIMARKS standards for metadata, and filling genomic gaps in non‐Western populations to enhance model generalizability and drive clinical translation [113]. For multi‐niche joint modeling, statistical adjustments are paramount. We recommend leveraging mixed‐effects models (e.g., MaAsLin2) or specialized tools like DeBIAS‐M to correct for batch and technical biases while adjusting for covariates. Researchers should systematically perform sensitivity analyses—varying denoising methods, rarefaction/dilution strategies, taxonomic databases, and batch correction algorithms—to quantify the prediction robustness of the MA model. Specifically for low‐biomass niches (e.g., skin), integrating negative controls with computational decontamination pipelines (e.g., Decontam) is mandatory to ensure data integrity and reproducibility [114, 115, 116, 117].

## MA AND HUMAN AGING

**Figure 2:**
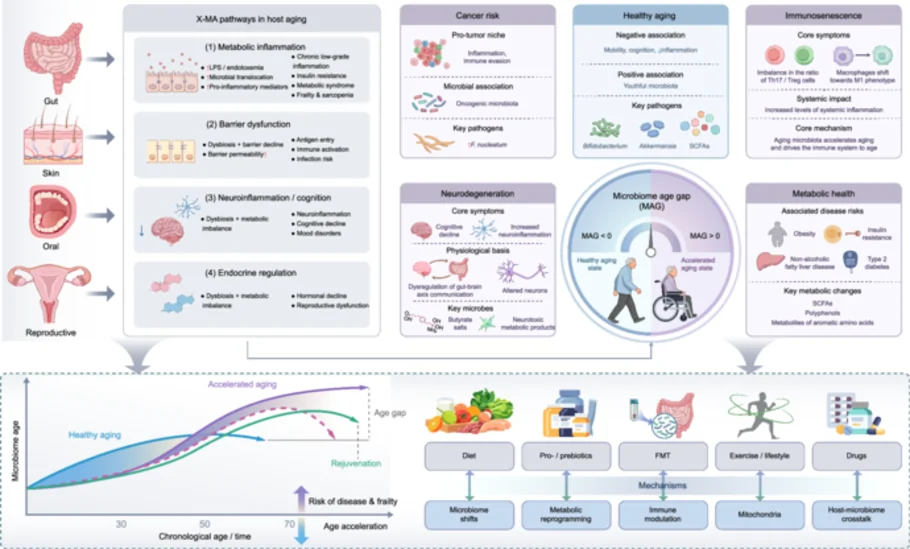
Dynamic trajectory of the microbiome age gap and its systemic health implications. The microbiome age gap (MAG), defined as the deviation between predicted MA and chronological age, has emerged as an informative indicator of biological aging. In general, a negative MAG (MAG < 0) signifies younger biological age, while a positive MAG (MAG > 0) indicates accelerated aging and elevated health risks. MA is closely linked to a full spectrum of health outcomes: youthful microbiome profiles in healthy aging correlate with improved gait speed, enhanced cognitive function, and reduced systemic inflammation, whereas aging‐related dysbiosis is associated with higher risks of tumorigenesis, neurodegenerative diseases, immunosenescence, and metabolic disorders. Critically, MA has marked plasticity, and can be modulated via dietary intervention, pro‐/prebiotic supplementation, fecal microbiota transplantation (FMT), exercise and lifestyle adjustments, and drug therapy (e.g., metformin, acarbose)—strategies that drive microbial rejuvenation and narrow the MAG. Underlying mechanisms include microbiome remodeling, metabolic reprogramming (e.g., modulation of SCFAs and polyamines), immune modulation, mitochondrial optimization, and altered host‐microbiome crosstalk. Collectively, these observations support MA as a potentially modifiable biomarker for aging risk stratification and intervention development.

The age of the microbial community serves as a crucial indicator reflecting the aging status of the host's microbial community. It has a close causal relationship and dynamic regulatory connection with health lifespan. Its changing characteristics can serve as an important biomarker for distinguishing healthy aging from pathological aging (Figure 2 and Table 2). Current evidence linking MAG to healthspan is most robust for the gut, whereas data for skin, oral, and reproductive sites remain nascent. The directionality, magnitude, and clinical implications of MAG vary significantly by niche. Consensus reviews by Tseng et al. and Chen et al. define MAG as the deviation of microbial composition and function from age‐matched healthy trajectories: a positive gap (accelerated aging) predicts adverse outcomes, whereas a negative gap (rejuvenated profile) correlates with longevity, preserved function, and enhanced survival [118, 119]. Tseng et al. [118] demonstrated that increased “uniqueness” of the gut microbiome in the elderly correlates with 4‐year survival. Mechanistically, Nuzum et al. [120] identified that enrichment of secondary BAs‐metabolizing bacteria is a key longevity signature in centenarians. Conversely, Chen et al. [119] reported a significantly elevated gut‐MA in type 1 diabetic patients compared to their chronological age. Pathological aging is characterized by reduced diversity and depleted SCFAs producers [121]. Min et al. [6] revealed that such dysbiosis drives sarcopenia via chronic inflammation. Regarding cognition, Chaudhari et al. [122] confirmed that microbial signatures predict cognitive decline risk. Varesi et al. and Wang et al. summarized that Alzheimer's disease and mild cognitive impairment are marked by pro‐inflammatory taxa enrichment and butyrate producer depletion [123]. Son et al. [124] identified “brain age” as a mediator linking dysbiosis to cognitive impairment, while Laske et al. [125] provided the first evidence that microbial features predict conversion to dementia over 4 years.

**Table 2:** Core logical framework.

Level	Core issue	Biological significance	Clinical/functional correlation
Concepts and Definitions	What is the microbiome age gap	The extent to which the composition and function of the microbiota deviate from the trajectory of a healthy state at the same age	Positive difference (older) → Accelerated aging; Negative difference (younger) → Healthy longevity
Prediction and Association	What does it predict	Panoramic biomarkers integrating multi‐dimensional physiological aging information	Healthy lifespan, physical fitness/weakness, cognitive function
Cause and Effect and Intervention	Can it be reversed	The young microbiome carries “biological information” that promotes health and extends lifespan.	Symbiotic coexistence at different times/Microbiota transplantation prolongs lifespan and improves cognition

The longevity effect of a youthful microbiota is validated in animal models. Smith et al. [126] showed that transplanting microbiota from young fish into middle‐aged killifish significantly extended lifespan and vitality. Yu et al. [127] demonstrated that fecal transplants from young mice reverse cognitive, immune, and inflammatory aging phenotypes in old recipients. However, Olejnik et al. [128] caution that the causal direction between dysbiosis and frailty requires further longitudinal validation.

Distinct from the gut‐MA, skin‐MA, oral‐MA, and reproductive‐MA reflect specific dimensions: barrier function, mucosal immunity, cognitive risk, and hormonal status, respectively. Zhai et al. [129] observed increased skin microbial diversity and loss of site specificity with age, with forearm sites showing earlier senescence. Li et al. [130] correlated skin micro‐ecology with antimicrobial peptides (e.g., claudin‐1) and physiological parameters, suggesting microbiota involvement in skin aging via barrier function. For the oral‐MA, Chiu et al. [131] found a dose‐dependent association between *Porphyromonas gingivalis* infection and age‐related macular degeneration (AMD); high antibody titers increased early AMD risk by over double. Magnusson et al. [132] further elucidated that this pathogen induces microglial activation and tau phosphorylation via NOX4. Similarly, reproductive microbiota undergoes significant age‐related shifts. Qin et al. [133] analyzed 6755 women, identifying age 45 as a structural turning point; *Lactobacillus*‐dominated communities correlated with higher live birth rates (OR = 3.62 and 5.39). Vivekanandan et al. [134] confirmed that *Lactobacillus vaginal* suppositories improve vaginal health indices in perimenopausal women with bacterial vaginosis. Within multi‐omics integration frameworks, Wilmanski et al. [26] utilized MA models to stratify individuals based on differential metabolic responses to dietary interventions, suggesting that microbial maturity dictates host responsiveness. Galkin et al. [9] proposed that gut‐MA serves as a stratification tool for metabolic health, with predictions closely aligning with inflammatory and lipid metabolism abnormalities. Furthermore, microbiome enterotypes have been linked to disease risk and treatment response. However, effective integration requires more than simple summation or averaging of multi‐site data; it demands a foundational understanding of the distinct biological meanings, technical comparability, and correlation coefficients across sites.

Considering that the MA in various ecological niches of the human body exhibits significant site‐specificity, this review will successively discuss the characteristic changes in MA in the gut, skin, oral, and reproductive tract, and elucidate their correlations with disease susceptibility. Furthermore, we explored the feasibility of integrating MA across different sites and proposed that “inflammatory aging” might be the core mechanism underlying the convergence of MA in different ecological niches.

### The gut microbiome: A dynamic ecological timer and systemic regulatory hub

The gut microbiome—a consortium of trillions of microorganisms often termed the “forgotten organ”—harbors a genetic repertoire exceeding that of the human host by several orders of magnitude, encoding a vast array of metabolic and biochemical pathways [105]. Far from being passive commensals, these microbial communities function as a dynamic metabolic organ and an immunological training ground, deeply involved in nutrient extraction, energy homeostasis, immune education, neuro‐signaling, and barrier defense. Unlike the relatively static host genome, the gut microbiome is highly plastic and dynamic, profoundly shaped by diet, lifestyle, pharmacology, and aging itself. This plasticity positions it as a critical interface connecting environmental exposures to host aging phenotypes, serving as a potential leverage point for modulating senescence trajectories. It is crucial to clarify that while the gut niche commands significant research attention due to its biomass and systemic influence, other niches—such as the oral cavity, skin, and reproductive tract—follow unique aging trajectories. These distinct sites act synergistically through shared terminal pathways. Consequently, understanding the gut is not an end in itself, but a linchpin for comprehending the panoramic view of how multi‐niche microbial networks shape human aging.

### Lifecycle ecological succession: Trajectories from colonization and homeostasis to dysbiosis

**Figure 3:**
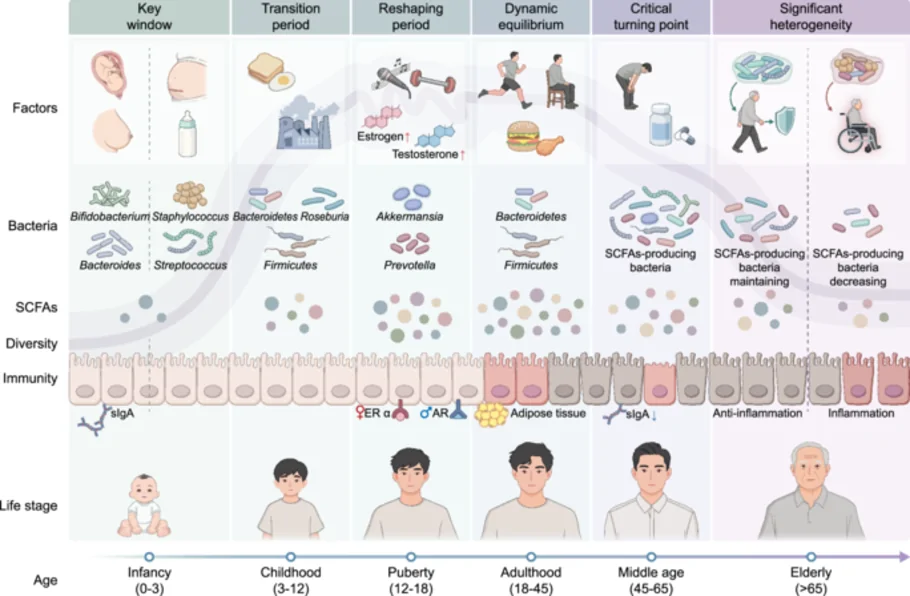
Dynamic changes in gut microbiome composition and function across the human lifespan. The horizontal axis (from left to right) represents sequential life stages across the human lifespan: infancy, childhood, puberty, adulthood, middle age, and elderly. The vertical axis (from top to bottom) depicts key annotated elements: factors, bacteria, short‐chain fatty acids (SCFAs), diversity, immunity, life stage, and age, collectively illustrating the trajectory of microbial characteristics and their determinants across distinct life phases. Both SCFAs levels (depicted as circles) and gut microbiome diversity (represented by the purple curve) exhibit a characteristic pattern of increase with age, followed by a subsequent decline.

The assembly and evolution of the gut microbiome represent a continuous ecological process throughout the host's lifespan. This trajectory is sequentially sculpted by stage‐specific factors—including early‐life colonization, dietary transitions, pubertal upheavals, immunosenescence, and chronic disease—manifesting distinct age‐stratified signatures (Figure 3).

Early life represents a pivotal “programming” window for gut microbial establishment. Birth mode is the primary determinant of the initial inoculum. Vaginally delivered infants acquire maternal vaginal and gut microbiota (e.g., *Lactobacillus*, *Prevotella*), whereas cesarean‐section newborns are predominantly colonized by maternal skin and environmental microbes (e.g., *Staphylococcus, Propionibacterium*), resulting in lower diversity and associations with increased risks of allergies and autoimmunity [135]. Feeding strategy acts as the second major driver. Breastfeeding provides not only passive immunity but also abundant human milk oligosaccharides (HMOs). These indigestible prebiotics selectively promote the proliferation of beneficial microbiota like *Bifidobacterium* [135]. These early colonizers ferment HMOs to produce SCFAs, lowering gut pH, inhibiting pathogens, and educating the developing mucosal immune system to establish immune tolerance. The introduction of solid foods triggers the first major ecological restructuring. Increased dietary complexity drives the expansion of taxa within *Bacteroidetes* and *Firmicutes* capable of metabolizing complex polysaccharides, significantly enhancing both diversity and SCFAs production capacity, thereby transitioning the ecosystem toward an “adult‐like” configuration [136]. During childhood, the gut microbiome gradually converges toward a stable adult configuration, dominated by *Firmicutes* and *Bacteroidetes*, with inter‐individual variability narrowing compared to infancy [135, 137]. During this phase, SCFAs‐producers (e.g., *Lachnospiraceae, Ruminococcaceae*) stabilize, and the microbiome's functional gene repertoire (involved in vitamin synthesis and complex polysaccharide metabolism) becomes fully equipped, establishing a symbiotic homeostasis with the host. Puberty marks a critical differentiation point. Surging sex hormones induce sexual dimorphism in the gut microbiota. Estrogen, for instance, influences gut permeability and mucus secretion, indirectly promoting the enrichment of microbiota like *Akkermansia* [138]. Conversely, androgens may modulate specific bacterial growth by regulating host immune status [139]. The establishment of these sex‐specific signatures signifies the completion of “adult‐like” microbial maturation, laying the groundwork for adult homeostasis. Although adulthood is typically viewed as a stable plateau—maintaining steady levels of *Bacteroides, Prevotella*, and *Lachnospiraceae* [140, 141]—subtle yet directional shifts emerge from middle age. Even in clinically healthy individuals, these changes foreshadow a transition from “platform” to “decline.” They include a slow but significant decrease in alpha diversity, a reduction in health‐associated commensals (e.g., butyrate‐producing *Lachnospiraceae* members) [142, 143], and a relative increase in opportunistic pathogens (e.g., *Enterobacteriaceae*). While asymptomatic, these early shifts lay the groundwork for systemic chronic inflammation and metabolic dysregulation.

In older adulthood, microbial trajectories exhibit profound heterogeneity, clearly differentiating two distinct patterns based on overall health status (specifically the frailty index). Healthy aging is often characterized by the maintenance of high diversity and “youthful” features, such as sustained levels of *Akkermansia* and butyrate producers [144]. This resilient structure correlates with healthy lifestyle factors, including adherence to a Mediterranean diet, regular physical activity, and rich social engagement. In stark contrast, older adults with frailty syndromes and multimorbidity often exhibit “frailty‐associated dysbiosis” [145, 146]: a dramatic loss of diversity, depletion of core beneficial symbionts (e.g., *Bifidobacterium, Faecalibacterium prausnitzii*), expansion of opportunistic pathogens (e.g., *Proteobacteria, Enterococcus*), and significant impairment of beneficial functions like SCFAs synthesis [21, 147]. This disorderly state strongly correlates with elevated systemic inflammatory markers (e.g., CRP, IL‐6) and independently predicts mortality, hospitalization risk, and cognitive decline. Drivers of this collapse are multifactorial, encompassing age‐related declines in gastrointestinal motility, digestive secretions, immunosenescence, widespread inadequate fiber intake, polypharmacy (especially antibiotics and proton pump inhibitors), and chronic comorbidities.

### Systemic regulatory networks: The “gut‐X axis” as a common pathway in multi‐organ aging

**Figure 4:**
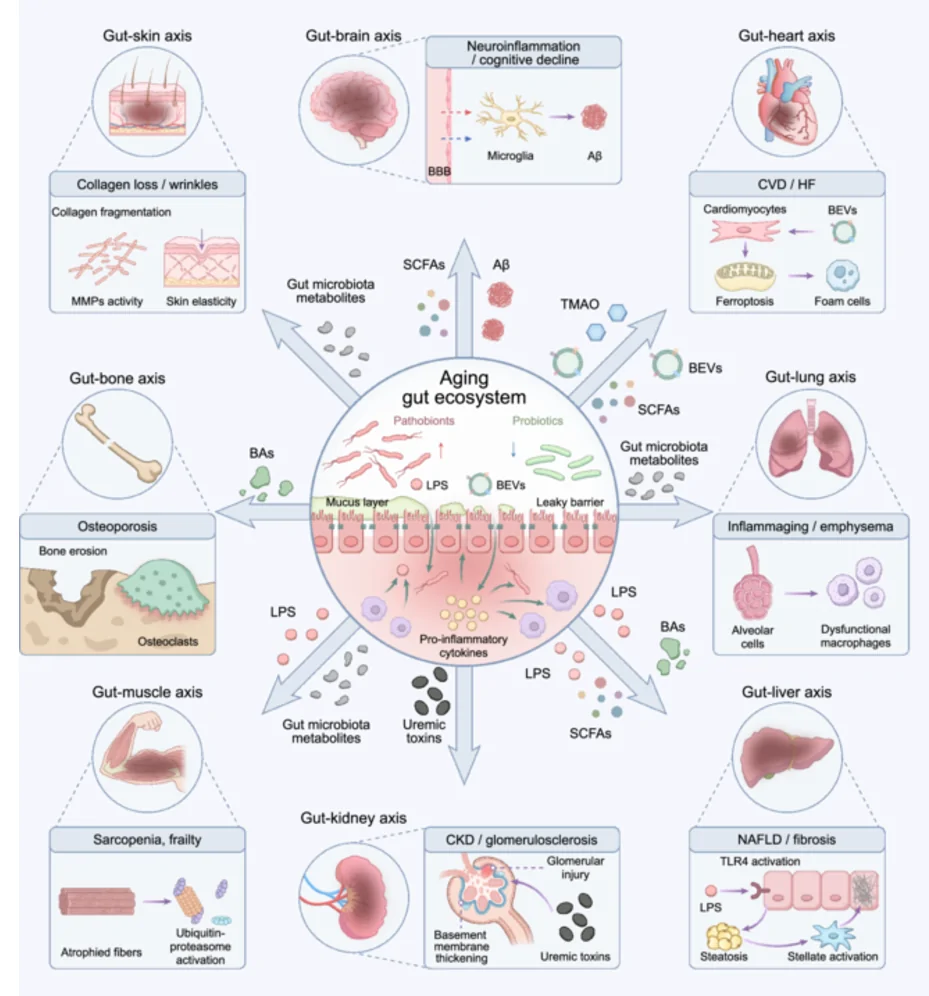
The aging gut connects with multiple organs via the “gut‐X” axis. The central circular diagram depicts the aged gut microenvironment, characterized by microbial dysbiosis with relative pathogen enrichment, impaired intestinal barrier integrity (“leaky gut”), tight junction disruption, and mucus layer degradation. Enhanced paracellular permeability allows luminal factors—including lipopolysaccharide (LPS), microbial metabolites, bacterial extracellular vesicles (BEVs), trimethylamine oxide (TMAO), bile acids (BAs), uremic toxins, amyloid‐β protein (Aβ), altered short‐chain fatty acid (SCFAs) levels, and pro‐inflammatory cytokines—to enter the systemic circulation. Eight blue arrows denote the bidirectional “gut‐X” axis, illustrating how these gut‐derived mediators disseminate to peripheral target organs and drive specific age‐related pathologies. Peripheral annotations detail site‐specific pathophysiological mechanisms: neuroinflammation and cognitive decline in the brain; cardiomyocyte ferroptosis and foam cell formation in the heart, contributing to cardiovascular disease (CVD) and heart failure (HF); enzymatic collagen degradation in the skin; alveolar macrophage dysfunction in the lungs, promoting inflammatory emphysema and chronic kidney disease (CKD); TLR4‐mediated steatosis and fibrosis in the liver, further develop into non‐alcoholic fatty liver disease (NAFLD); toxin‐driven renal injury and chronic kidney disease; sarcopenia and muscle atrophy in muscle; and enhanced osteoclast activity with bone loss in the skeletal system.

The gut microbiome does not merely influence local intestinal health; it constitutes a dense, bidirectional communication network with distal organs via metabolites, immune modulation, and neuro‐signaling. During aging, gut dysbiosis disrupts the homeostasis of these axes, serving as a pivotal upstream driver of systemic senescence (Figure 4).

The gut houses ~70% of the body's immune cells [148]. Dysbiosis drives systemic, chronic, low‐grade inflammation (“inflammaging”) through several mechanisms [148]: Aging reduces tight junction protein expression and thins the mucus layer, increasing permeability (“leaky gut”). This permits translocation of Microbe‐Associated Molecular Patterns (MAMPs), such as LPS, into the portal circulation [149]. LPS binds to LPS‐binding protein and engages TLR4 on monocytes/macrophages, activating NF‐κB and NLRP3 inflammasomes, leading to massive release of IL‐1β, IL‐6, and TNF‐α [150]. Depletion of beneficial symbionts (e.g., *Faecalibacterium prausnitzii*) reduces SCFAs production. Butyrate, a potent HDAC inhibitor, promotes Treg differentiation and suppresses pro‐inflammatory Th17 responses [151]. Its deficiency tilts the Th17/Treg balance toward inflammation. Concurrently, the loss of immunomodulatory taxa like *Akkermansia* further weakens mucosal tolerance [151]. Persistent low‐dose LPS stimulation can induce a prolonged state of innate immune hyper‐responsiveness (“trained immunity”), maintaining a chronic pro‐inflammatory secretion even without new pathogens [150].

The gut‐brain axis integrates neural, endocrine, immune, and metabolic pathways. Age‐related dysbiosis is a key instigator of neurodegeneration. Gut‐derived LPS and pro‐inflammatory cytokines can traverse the bloodstream or vagus nerve afferents to impact the brain [152]. They compromise the blood‐brain barrier (BBB), activating microglia and astrocytes to generate amyloid‐β protein (Aβ) and adopt a pro‐inflammatory phenotype. This releases reactive oxygen species, causing synaptic plasticity impairment and neuronal damage [153]. Elevated LPS and bacterial 16S rRNA have been detected in the CSF and brain tissues of Alzheimer's disease patients [19]. Butyrate and propionate cross the BBB, exerting neuroprotective effects via HDAC inhibition or GPCR activation, thereby promoting BDNF expression. The age‐related decline in SCFAs weakens this neuronal safeguard [154, 155]. Dysbiosis leads to the accumulation of uremic toxins like indoxyl sulfate and phenylacetylglutamine, which induce endothelial senescence and impair BBB function [156]. Recent studies highlight that certain secondary BAs (e.g., Aβ) accumulate aberrantly in the brain, directly activating microglia and driving neuroinflammation [157]. The gut produces ~90% of the body's serotonin, a process modulated by microbiota. Microbes also influence precursors for GABA and dopamine [158]. Dysbiosis disrupts these neuroactive pools, affecting mood and cognition.

As a metabolic hub, the gut microbiome governs glucose, lipid metabolism, and energy homeostasis, positioning it centrally in metabolic syndrome and cardiovascular senescence. Dietary choline and l‐carnitine are metabolized by gut microbes into trimethylamine (TMA), which the liver converts to TMAO. TMAO promotes macrophage foaming, endothelial dysfunction, and platelet hyperreactivity, serving as an independent risk factor for atherosclerosis and thrombosis [157]. Gut microbes convert primary BAs into secondary BAs. These act as signaling molecules via FXR and TGR5 receptors, regulating glucose homeostasis, lipid metabolism, and energy expenditure [159, 160]. Age‐related dysbiosis alters the BAs pool, disrupting these pathways and linking to insulin resistance and non‐alcoholic fatty liver disease (NAFLD). Phenylacetate induces endothelial cell senescence [161, 162]. Conversely, butyrate upregulates barrier function and inhibits cardiomyocyte ferroptosis, conferring cardiac protection [163, 164]. Furthermore, bacterial extracellular vesicles (BEVs)—carrying LPS and other effectors—translocate into circulation upon barrier breach, exacerbating cardiac fibrosis and inflammation. Notably, engineered BEVs offer a novel nanomedicine platform for targeted delivery of protective cargos to restore gut‐heart axis functionality [165].

Sarcopenia and dysbiosis exhibit a vicious cycle. Sarcopenia reduces physical activity, altering gut transit, while dysbiosis accelerates muscle loss via: (1) Systemic Inflammation: LPS and cytokines activate the ubiquitin‐proteasome system and autophagy‐lysosome pathways, upregulating Atrogin‐1 and MuRF‐1 while suppressing IGF‐1/Akt/mTOR signaling, causing net protein loss [166]. (2) SCFAs Depletion: butyrate activates PGC‐1α, promoting mitochondrial biogenesis and oxidative metabolism in muscles [167]. (3) Mitochondrial Dysfunction: dysbiosis‐induced metabolic shifts impair muscle energy metabolism [168]. Microbes regulate bone metabolism via: (1) Immunomodulation: Pro‐inflammatory cytokines (TNF‐α, IL‐1β, IL‐6) stimulate osteoclast formation and inhibit osteoblast activity [169]. (2) Mineral Absorption: SCFAs lower luminal pH, increasing solubility and passive absorption of calcium and magnesium; butyrate also upregulates epithelial calcium channels. (3) Hormones and Metabolites: microbes synthesize Vitamin K (essential for osteocalcin carboxylation), and specific secondary BAs modulate osteoblast/osteoclast function.

Furthermore, the liver receives blood directly from the portal vein. “Leaky gut”‐derived LPS activates hepatic Kupffer cells, driving inflammation and fibrosis in NAFLD [170]. BAs recirculation is central to gut‐liver crosstalk; dysbiosis‐altered BAs profiles directly impact hepatic metabolic and inflammatory states [159]. Sharing a common mucosal immune system, gut dysbiosis exacerbates pulmonary inflammation via circulating immune cells and metabolites. This axis is critically disrupted in aging and COPD [149, 171]. Gut‐derived uremic toxins (indoxyl sulfate, p‐cresol sulfate) accumulate as renal function declines, further promoting renal fibrosis and creating a vicious cycle [172, 173]. Systemic inflammation and microbial metabolites impact skin aging. Pro‐inflammatory cytokines accelerate collagen degradation [174]. Specific probiotics (e.g., *Bifidobacterium longum*) and their metabolites can increase ceramide synthesis, improving barrier function and counteracting skin aging [175]. From the above, it can be seen that the intestinal microbiota plays a crucial role in maintaining the normal functions of human organs and has an all‐round impact on the body. This influence acts imperceptibly on the natural aging process of the human body, and is receiving increasing attention in the current era.

**Figure 5:**
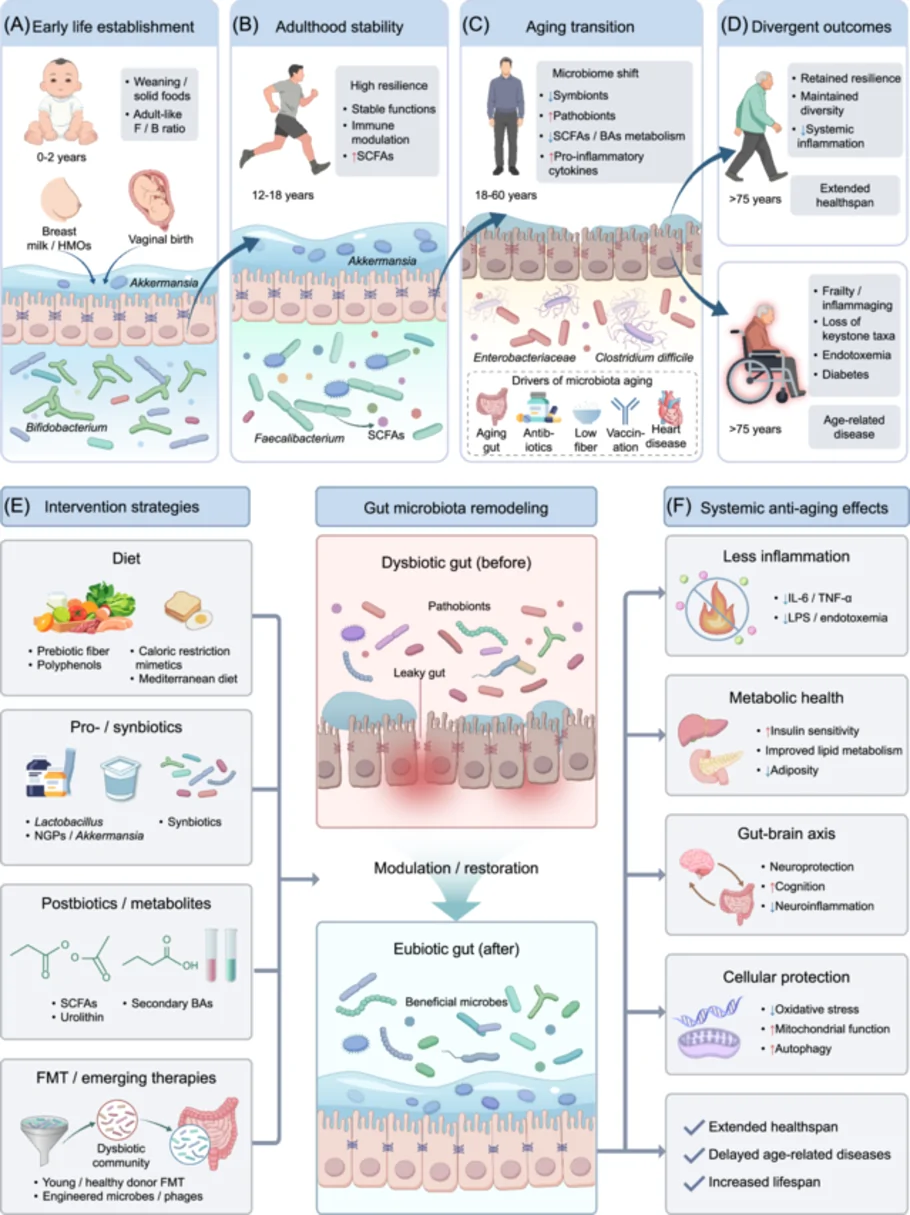
Lifespan dynamics of the human gut microbiome and microbiome‐targeted anti‐aging interventions. (A–D) This panel depicts the full lifespan trajectory of the human gut microbiome with a focus on aging‐related remodeling, covering four consecutive life stages: early‐life establishment and maturation (A; 0–2 years), driven by delivery mode, breastfeeding (HMOs), and weaning, featuring Bifidobacterium dominance and progressive diversification toward an adult‐like *Firmicutes*/*Bacteroidetes* (F/B) ratio; adulthood homeostatic stability (B; 12–18 years), characterized by a high‐resilience eubiotic state dominated by beneficial symbionts *Akkermansia* and *Faecalibacterium*, with robust short‐chain fatty acid (SCFA) production and immune modulatory function; the aging transition phase (C; 18–60 years), defined by the canonical microbial signature and key drivers of microbiome aging (MA), including reduced abundance of beneficial symbiotic microbiota, decreased SCFA production, impaired bile acid (BA) metabolism, increased opportunistic pathogens, and elevated pro‐inflammatory cytokines; and divergent outcomes in later life (D; >75 years), ranging from preserved resilience and healthy aging to dysbiosis‐associated frailty and age‐related disease. (E, F) This panel illustrates gut microbiome‐targeted anti‐aging intervention strategies (E), including dietary modification (prebiotics, fiber), probiotics/synbiotics, postbiotics (e.g., SCFAs), and fecal microbiota transplantation (FMT) from young donors. These strategies aim to restore microbial homeostasis and intestinal barrier integrity (F) and modulate systemic aging‐associated processes, including chronic low‐grade inflammation, metabolic dysfunction, oxidative stress, and gut–brain axis dysregulation, thereby supporting healthy aging and reducing age‐related disease risk.

Given the pivotal role and high plasticity of the gut microbiome in aging, targeted modulation of the microbial ecosystem has emerged as a frontier strategy for delaying senescence and preventing age‐related pathologies (Figure 5). Dietary Modulation as a Cornerstone. Diet is the most potent modulator of the gut microbiota [176]. Fiber‐rich dietary patterns, such as the Mediterranean diet, serve as the cornerstone for sustaining SCFAs producers and mitigating inflammation [177]. Polyphenols exert bidirectional regulatory effects: they are metabolized by microbes into bioactive compounds that activate host antioxidant pathways, while simultaneously exerting selective antimicrobial pressure against pathobionts [178]. Evolution of Microbial Therapies: The field is witnessing a generational shift. While traditional probiotics aim for general ecosystem balance, next‐generation probiotics are rationally designed to target specific nodes of the aging process [38]. *Akkermansia muciniphila* has emerged as a leading candidate for ameliorating metabolic aging, owing to its unparalleled capacity to restore mucosal barrier integrity [179]. Specific *Lactobacillus plantarum strains* have demonstrated efficacy in enhancing BDNF expression via the gut‐brain axis, thereby improving cognitive function [180]. Furthermore, breakthroughs in targeting the gut‐muscle axis reveal that interventions with *Lactobacillus rhamnosus* and *Faecalibacterium prausnitzii* significantly improve muscle mass, strength, and mitochondrial function in aged murine models, providing viable candidates for sarcopenia intervention [181]. To circumvent the challenge of engraftment associated with live biotherapeutics, disorderly and postbiotics offer promising alternatives with enhanced stability and safety profiles. For cases of severe dysbiosis, Fecal Microbiota Transplantation (FMT) presents an opportunity for comprehensive ecosystem reconstruction, although long‐term safety and standardization protocols require further investigation. Complementary lifestyle interventions—including regular physical exercise, adequate sleep hygiene, and stress management—exert synergistic effects on maintaining microbial health.

The trajectory of this field is moving toward precision nutrition and microbiome therapeutics, guided by individual multi‐omics profiles (microbiome, metabolome, genome). Emerging technologies, such as genetically engineered probiotics and phage therapies, promise unprecedented specificity and safety in reshaping the gut ecosystem for healthy aging.

### The oral microbiome: A sentinel of mucosal aging and a systemic regulatory outpost

The oral microbiota constitutes the second largest and most heterogeneous microbial habitat in the human body. Its complex anatomy—encompassing teeth, gingiva, buccal mucosa, tongue, and palate—creates steep ecological gradients ranging from aerobic to strictly anaerobic niches, hosting a highly diverse microbial community. Traditionally, research focused on oral microbiota in the context of local infectious diseases like caries and periodontitis. However, the past two decades have witnessed a paradigm shift. Extensive epidemiological, clinical, and mechanistic studies now link oral homeostasis to systemic conditions, including cardiovascular disease, diabetes, Alzheimer's disease, rheumatoid arthritis, and adverse pregnancy outcome. This strongly suggests that the oral cavity is not an isolated “black box” but a dynamic interface deeply interacting with systemic health; oral dysbiosis serves as both an early warning signal and an active driver of systemic aging processes.

#### Lifecycle dynamics: Mapping the age‐trajectories of the oral microbiome

**Figure 6:**
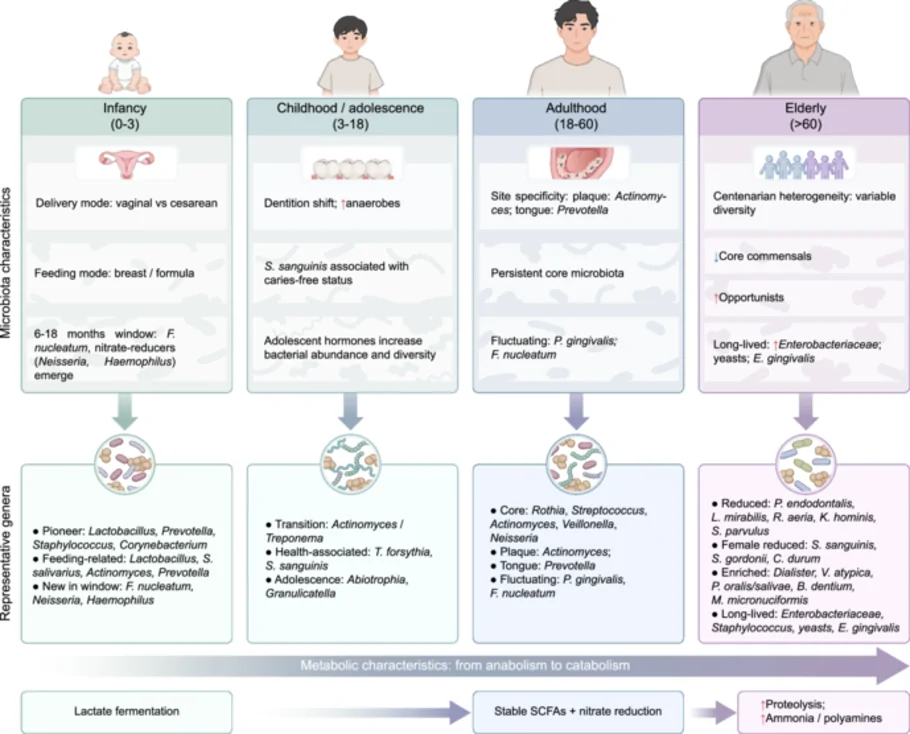
Lifecourse succession of the oral microbiome. This schematic summarizes the dynamic succession of the human oral microbiome across four major life stages: infancy (0–3 years), childhood/adolescence (3–18 years), adulthood (18–60 years), and elderly (>60 years). In infancy, initial colonization is shaped by delivery and feeding modes; notably, *Neisseria*, *Haemophilus*, and *Fusobacterium nucleatum* increase markedly at 6–18 months. Childhood/adolescence (primary‐to‐permanent dentition transition) brings increased complexity and anaerobe prevalence; pubertal hormonal changes elevate bacterial load/diversity and enrich *Abiotrophia* and *Granulicatella*. Adulthood is characterized by a stable core microbiota (*Rothia*, *Streptococcus*, *Actinomyces*, *Veillonella*, *P. melaninogenica*, *Neisseria*) with strong site‐specificity and temporal variability. In elderly, core commensals (*P. endodontalis*, *L. mirabilis*, *R. aeria*, *K. hominis*, *S. parvulus*) decline—with female‐specific reductions in *S. sanguinis*, *S. gordonii*, and *C. durum*—while opportunistic pathogens (*Dialister*, *V. atypica*, *P. oralis/salivae*, *B. dentium*, *M. micronuciformis*) are enriched. The lower panel illustrates a functional shift from anabolism to catabolism: lactate fermentation (infancy), stable SCFAs profiles and nitrate reduction (adulthood), and enhanced proteolysis with elevated polyamines (elderly).

The establishment and development of the oral microbiome is a continuous ecological process tightly coupled with host development and physiological changes. This trajectory is sequentially sculpted by stage‐specific factors, including initial inoculation sources, tooth eruption, dietary patterns, hormonal levels, and immune status (Figure 6).

Early life represents a “critical window period” for the colonization of core oral microbiota and the establishment of ecological relationships; the direction of microbial succession during this phase may exert long‐term effects on oral and even systemic health risks for decades to come. Delivery mode is the primary determinant of the initial inoculum: neonates born via vaginal delivery first encounter maternal vaginal flora, dominated by *Lactobacillus*, *Prevotella*, and *Snodgrassella*. In contrast, cesarean‐delivered infants acquire more microbes from maternal skin and the hospital environment, such as *Staphylococcus, Corynebacterium*, and *Propionibacterium* [182], typically exhibiting lower early diversity. Feeding strategy acts as another key ecological driver. Breastfeeding provides not only nutrition and immune factors but also abundant HMOs, selectively promoting the colonization and growth of beneficial symbionts like *Streptococcus salivarius* and *Actinomyces*. Formula feeding, conversely, may facilitate the earlier emergence of microbiota like *Bacteroidetes* [183]. Approximately at 6 months of age, the eruption of the first tooth marks a “revolutionary event” in oral microbial ecology. Tooth hard tissues provide non‐shedding surfaces for attachment, while the formation of the gingival sulcus creates a unique low‐oxygen niche, enabling the successful colonization of strict anaerobes like *Fusobacterium nucleatum* [184]. As a “bridge bacterium,” *Fusobacterium nucleatum*‐aggregates various bacteria, proving crucial for the three‐dimensional architecture of dental plaque biofilm. During this period, nitrate‐reducing bacteria (e.g., *Neisseria, Haemophilus*) also show significantly increased detection rates [185].

With the establishment of the primary dentition and its replacement by permanent teeth, the diversity of the childhood oral microbiome continues to increase, and its structure becomes progressively more complex. Microbial composition in permanent tooth‐associated plaque differs from that of deciduous teeth; for instance, *Actinomyces* species are more abundant in permanent dentition, while spirochetes are more common in deciduous teeth [186, 187]. Certain bacteria, such as health‐associated *Streptococcus sanguinis*, exhibit a positive correlation with caries‐free status. Upon entering puberty, surging sex hormone levels significantly impact the oral microenvironment [188]. Increased estrogen and testosterone levels lead to heightened gingival capillary permeability and increased gingival crevicular fluid exudate, providing richer nutrients for bacterial growth. This results in increased susceptibility to gingivitis, characterized mainly by fluctuations in the quantity of existing flora rather than the introduction of entirely new species [189]. Overall, adolescent oral microbial diversity exceeds that of childhood, with inter‐individual variability narrowing, converging towards the adult pattern.

The healthy adult oral microbiome exhibits high individuality based on site‐specificity (e.g., tongue dorsum, buccal mucosa, supragingival/subgingival plaque) while concurrently harboring a “core microbiome” consistently present across most individuals. The Human Microbiome Project identified genera such as *Streptococcus, Rothia*, *Veillonella*, *Actinomyces*, *Haemophilus*, and *Prevotella* as core across various oral sites [190]. Despite this individual “fingerprint,” the microbial community maintains a dynamic equilibrium throughout adulthood. This balance relies on intricate interspecies interactions, including symbiosis and competition. For example, genera like *Veillonella* and *Actinomyces* can reduce dietary nitrate to nitrite in saliva, subsequently converting it to nitric oxide (NO) [191]. NO lowers blood pressure, improves vascular function, and exhibits antimicrobial activity against periodontal pathogens, constituting a vital metabolic axis for oral‐systemic homeostasis. Additionally, bacteria like *Haemophilus parainfluenzae* inhibit pathogens by producing bacteriocins and hydrogen peroxide. *Veillonella* and *Actinomyces* can convert lactic acid into weaker acids, curbing the overgrowth of cariogenic bacteria [192], thereby exerting beneficial effects on oral health

Entering old age, physiological aging (e.g., diminished salivary gland function leading to reduced flow rate and altered composition), tooth loss, denture wearing, immunosenescence, and polypharmacy (especially medications with xerostomia side effects) collectively drive systematic changes in the oral microenvironment. This propels characteristic ecological restructuring of the oral microbiome, primarily trending towards a reduction in core symbiotic bacteria [188] and an increase in opportunistic pathogens, including fungi and *Enterobacteriaceae* [193]. Regarding changes in oral microbial α‐diversity during old age, studies report inconsistent conclusions: some document a decline [194], while others show a rebound after a mid‐life dip [188]. This heterogeneity likely stems from variations in the oral health status, lifestyles, and comorbidity profiles of study populations [190]. An insightful perspective suggests that diversity differences between younger and older populations may largely arise from shifts in low‐abundance taxa, whereas the relative stability of the core dominant flora might be more critical for maintaining health [190]. The oral microbiome of extreme longevity populations (e.g., centenarians) may exhibit particularly unique patterns; some studies report abnormally high diversity, while others show reductions [194, 195]. This implies that “healthy aging” may correspond to a distinct and resilient oral ecological state.

Accompanying taxonomic shifts is a fundamental transformation in the overall metabolic function of the microbial community. In healthy adulthood, oral microbial function is dominated by anabolism, such as carbohydrate fermentation and nitrate reduction [191]. However, during old age or under disorderly states, the community metabolic profile shifts strongly towards catabolism. Due to decreased salivary clearance, increased food debris retention, and the release of protein substrates from tissue destruction, microbial proteolytic activity intensifies significantly. This leads to the substantial production of cytotoxic and pro‐inflammatory metabolites like polyamines, ammonia, hydrogen sulfide, and butyric acid [196]. Notably, while butyrate is anti‐inflammatory in the large intestine, within the oral environment, it inhibits epithelial cell proliferation and disrupts tissue integrity, acting as a key virulence factor in periodontitis progression [192]. Therefore, this functional transition of the oral microbiome from “anabolism” to “catabolism” [196] is not only a driver of local tissue destruction but also potentially a mechanism for impacting systemic health via the release of deleterious metabolites.

#### Systemic regulatory networks: Multidimensional pathways linking oral microbiome to systemic senescence

**Figure 7:**
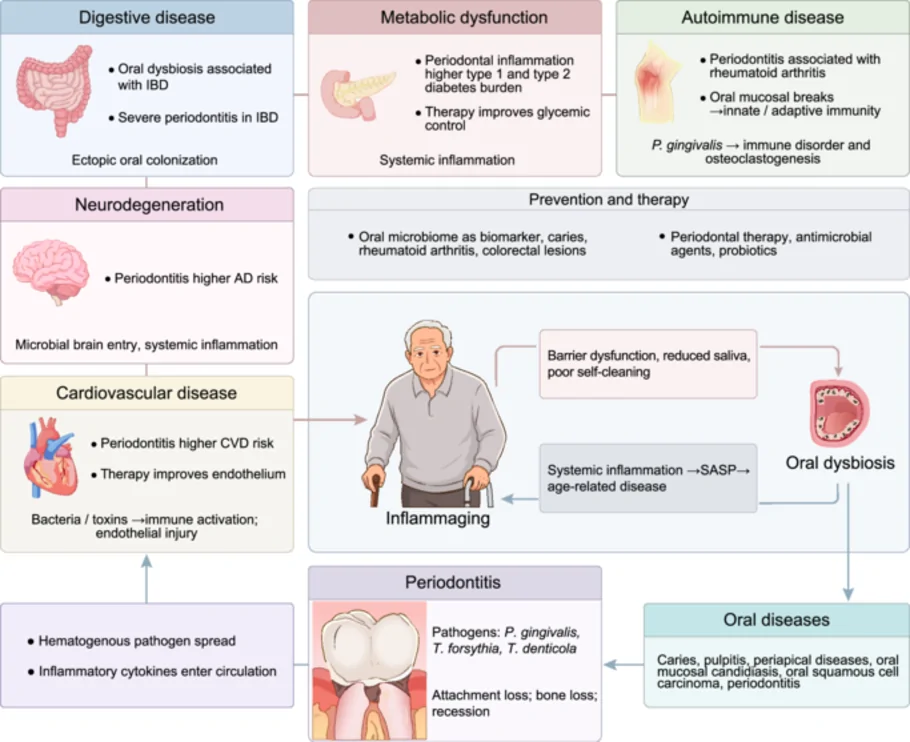
Oral microbiome aging links local craniofacial homeostasis to systemic health. This figure centers on the bidirectional crosstalk between oral microbial dysbiosis and inflammaging: inflammaging disrupts the oral microenvironment (reduced salivary flow, impaired immune surveillance) to drive dysbiosis (commensal loss, pathogen enrichment), while dysbiosis exacerbates systemic inflammation via bacteremia, inflammatory mediator spillover, and senescence associated secretory phenotype (SASP) dissemination, forming a pro‐aging feedback loop. Radiating from this core, five systemic conditions are linked to oral dysbiosis (e.g., periodontal pathogens), including cardiovascular disease (CVD), Alzheimer's disease (AD), diabetes, and non‐age‐related disorders, such as inflammatory bowel disease (IBD), rheumatoid arthritis, with key pathogenic mechanisms detailed for each. Potential applications are highlighted: (1) oral microbiome as biomarkers for risk prediction and early diagnosis (dental caries, rheumatoid arthritis, colorectal diseases); and (2) therapeutic targets for ecological restoration via periodontal therapy, probiotics/prebiotics, and pathogen‐directed interventions to modulate disease progression.

Oral dysbiosis, particularly chronic low‐grade infections exemplified by periodontitis, is far from an isolated local event [197]. In the context of aging, this ecological disruption actively participates in and accelerates systemic functional decline through highly interconnected biological pathways. At the heart of these mechanisms lies a self‐amplifying vicious cycle between the aging host's oral environment and the disorderly community, amplifying destructive signals systemically (Figure 7).

In aging individuals, periodontal tissues afflicted by chronic periodontitis act as a sustained, low‐grade inflammatory nidus [184]. Unlike acute infections, this state is characterized by the chronic overproduction of pro‐inflammatory mediators (e.g., IL‐1β, IL‐6, TNF‐α, PGE_2_) within the lesion [198]. Immunosenescence impairs the clearance of periodontal pathogens and dampens the resolution of inflammation, allowing this state to persist indefinitely [198, 199]. These locally produced cytokines continuously access the systemic circulation via the ulcerated pocket epithelium and rich vascular/lymphatic networks [199]. This pathophysiological process elevates baseline serum levels of acute‐phase reactants (e.g., CRP, fibrinogen) and pro‐inflammatory cytokines [200]. This “systemic low‐grade inflammation,” sustained by the oral focus, is a core hallmark of aging biology. It systematically alters the internal milieu, inducing endothelial dysfunction, promoting insulin resistance, and providing a pathological substrate for the onset and progression of age‐related diseases, including atherosclerosis [201, 202], type 2 diabetes [203, 204], and sarcopenia. In neurodegenerative diseases, oral microbiota may serve as a sentinel. Liu et al. [205] demonstrated that the detection of oral bacteria in the brain increases Alzheimer's disease risk by over tenfold, while *Porphyromonas gingivalis* positivity elevates risk by sixfold. Therefore, *Porphyromonas gingivalis* eradication may be an effective means to delay onset of Alzheimer's disease [206]. Thus, chronic oral inflammation is a direct effector pathway linking local dysbiosis to systemic pathology. Walford first linked immune dysfunction to aging in 1969, coining the term “immunosenescence,” characterized by naive T‐cell depletion, blunted effector functions, and chronic inflammation [207]. Oral dysbiosis, specifically the persistent colonization by periodontal pathogens, acts as a chronic exogenous antigenic stimulus, accelerating this process. Chronic periodontal infection provides relentless stimulation to the innate immune system. Repeated exposure to MAMPs (e.g., LPS) primes monocytes/macrophages towards a pro‐inflammatory phenotype and sustains low‐level cytokine secretion, exhausting immune homeostasis reserves [166]. In adaptive immunity, pathogens interfere with antigen presentation and disrupt balance by inducing Treg dysfunction or promoting pro‐inflammatory Th17 differentiation. Furthermore, through “molecular mimicry” and citrullination (catalyzed by *Porphyromonas gingivalis* peptidylarginine deiminase [208]), immune tolerance is broken, generating autoantibodies. This not only drives rheumatoid arthritis but also reflects systemic immune dysregulation during aging [208, 209]. Moreover, metagenomic shotgun sequencing and a metagenome‐wide association study reveal significant oral‐gut dysbiosis in rheumatoid arthritis (RA) patients [210]. Perricone et al. [208] further identified *Porphyromonas gingivalis* abundance and its peptidylarginine deiminase activity as independent predictors of autoimmune initiation risk. Thus, oral dysbiosis is not merely a consequence but a potent driver of immunosenescence.

Maintaining epithelial barrier integrity is paramount for defense. Age‐related barrier decline is universal, but oral dysbiosis actively accelerates periodontal pathogens (e.g., *Porphyromonas gingivalis*) directly degrade epithelial junctional proteins and extracellular matrix [211]. Concurrently, toxic metabolites (butyrate, hydrogen sulfide) inhibit epithelial proliferation and induce apoptosis [196]. This compromises gingival integrity, creating micro‐ulcers that facilitate microbial and toxin translocation [199]. With aging, reduced gastric acid and motility allow swallowed virulent oral pathogens to overcome gut colonization resistance. These ectopic colonizers further disrupt intestinal tight junctions, increasing gut permeability. This “leaky gut” allows endogenous endotoxins and metabolites to flood the systemic circulation, creating a synergistic “gut‐oral axis” of systemic barrier failure.

Cellular senescence is a cellular hallmark of aging. Oral dysbiosis induces and accelerates this process via multiple routes. The pro‐inflammatory microenvironment (high TNF‐α, IL‐1β, ROS) in periodontitis is a potent inducer of premature senescence in periodontal ligament fibroblasts and gingival epithelial cells [212]. These senescent cells secrete a senescence‐associated secretory phenotype (SASP)—rich in MMPs and inflammatory cytokines—which further degrades surrounding tissues, hinders repair, and amplifies local inflammation. Furthermore, chronic oral infection and the resultant systemic inflammation adversely affect systemic stem cell function. A chronic inflammatory milieu impairs the proliferation and differentiation capacity of mesenchymal stem cells, potentially linking alveolar bone loss to systemic osteoporosis. Systemic inflammatory cytokines also interfere with muscle satellite cell activation, impairing muscle regeneration and potentially connecting oral health to sarcopenia. Accompanying aging and dysbiosis, the oral microbial metabolic profile shifts towards a catabolic state, generating systemically toxic metabolites. The proteolytic breakdown of proteins yields hydrogen sulfide, ammonia, and high concentrations of butyrate [196]. In the context of AD, *Porphyromonas gingivalis* gingipains are not only detectable in patients' brains but can also impair the Aβ clearance rate of microglial cells [205, 213]. This suggests specific bacterial virulence factors possess remote, direct organ toxicity. Concurrently, in the elderly population, high‐titer IgG antibodies against key periodontal pathogens such as *Porphyromonas gingivalis* and periodontal probing depth are positively correlated with all‐cause dementia, suggesting that systemic inflammation induced by chronic oral infections may be a key mediator factor driving the risk of Alzheimer's disease through vascular damage pathways rather than direct neurodegenerative lesions [206].

Given the pivotal role of the oral microbiome in systemic aging, maintaining its ecological equilibrium constitutes a critical component of healthy aging strategies. Periodontal therapy as a cornerstone. Non‐surgical periodontal therapy remains the primary intervention to reverse dysbiosis. Treatment consistently shifts the subgingival microbiome from a disease‐associated profile back to a health‐associated one, concomitant with clinical improvement [201]. Crucially, periodontal therapy extends benefits beyond the oral cavity: it significantly improves endothelial function, reduces systemic inflammatory markers [201]. These findings cement periodontal therapy as a foundational strategy for modulating oral‐MA and systemic health. Anti‐virulence and pathogen‐specific approaches. Moving beyond broad‐spectrum disruption, precision therapies aim at specific pathogens or their virulence factors. Studies demonstrate that scaling and root planing combined with local tetracycline fiber delivery yields superior outcomes compared to monotherapy. Antimicrobial photodynamic therapy (aPDT) may reduce bleeding indices [214]. Most notably, in animal models, inhibitors targeting *Porphyromonas gingivalis* gingipains prevent Aβ deposition and rescue cognitive deficits [213, 215]. This provides compelling proof‐of‐concept for targeting oral pathogens to intervene in neurodegenerative diseases. Probiotics, prebiotics, and nitrate metabolism. Reshaping the local ecology can be achieved through: probiotics promote microbiota via competitive exclusion and immunomodulation [216, 217]. Arginine, as a prebiotic, is metabolized by commensals to ammonia, neutralizing cariogenic acid production [218]. Dietary nitrates selectively enrich nitrate‐reducing bacteria (e.g., *Neisseria*, *Rothia*). This shifts the metabolic profile from “nitrite consumption” to “NO generation,” elevating plasma nitrite, lowering blood pressure, and correlating with improved cognition in the elderly [219, 220, 221]. The effects of mouthwashes are paradoxical. Chlorhexidine, while antiseptic, raises blood pressure [222]. Long‐term use of over‐the‐counter mouthwashes is linked to increased risks of diabetes and hypertension [223]. Senotherapy, by clearing senescent cells and their SASP, may reduce inflammation and promote tissue regeneration [224]. Oral microbiome transplantation is a disruptive concept, though donor screening and standardization remain formidable challenges [225]. Precision interventions based on oral‐MA should follow age‐stratified principles: guiding healthy colonization in children [185], personalizing regimens based on inflammatory phenotypes in adults [226], and implementing multimodal interventions in the elderly while cautiously evaluating the risks of antiseptic mouthwashes.

### The skin microbiome: A cutaneous chronometer and barrier‐immune integrator

As the body's largest organ, the skin constitutes a complex physical, chemical, and immunological barrier against the external environment. This expansive surface hosts a complex and diverse microbial community—the skin microbiome—whose composition and function are highly site‐specific, strictly regulated by local microenvironments (humidity, temperature, sebum, pH) [83]. Traditionally, research focused on correlations with local inflammatory diseases like atopic dermatitis, psoriasis, and acne. However, accumulating evidence links skin microbial homeostasis to systemic aging and age‐related pathologies [227]. This paradigm shift reframes the skin microbiome from a local ecological niche to a dynamic interface ecosystem deeply interacting with systemic host physiology.

#### Age‐dependent ecological succession of the skin microbiome

**Figure 8:**
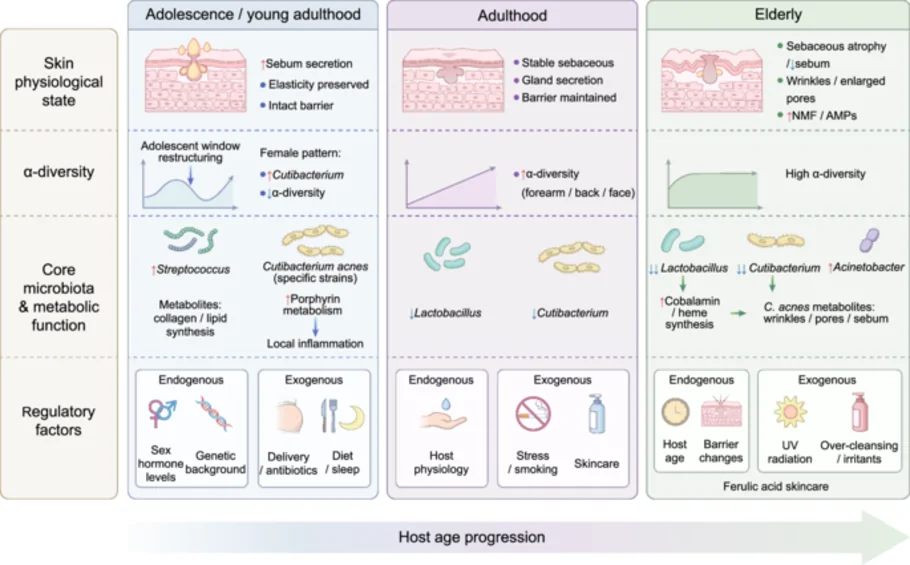
Mechanistic landscape of skin microbiome age dynamics across the host lifespan. This schematic illustrates age‐associated remodeling of the skin microbiome across adolescence/young adulthood, adulthood, and elderly, integrating four key dimensions: skin physiological state, microbial α‐diversity, community structure and metabolic function, and host/environmental regulatory factors. During youth, high sebum production, intact barrier integrity, and hormonally driven ecological shifts shape a distinct microbial landscape dominated by sebaceous‐associated commensals. Adulthood is characterized by relative physiological stability and microbial homeostasis, with balanced community composition and preserved functional resilience. In elderly, intrinsic aging and extrinsic stressors—including barrier deterioration, altered sebaceous function, and cumulative environmental exposure‐drive microbial dysbiosis, metabolic remodeling, and increased susceptibility to skin aging phenotypes. Regulatory influences across life stages include host physiology, sex hormones, lifestyle, skincare practices, environmental exposure, and targeted microbiome‐modulating interventions.

Skin aging is a multifactorial biological process characterized by impaired barrier function, diminished immune surveillance, structural laxity (e.g., wrinkles, reduced elasticity), and declining reparative capacity [83, 228]. This process is driven by intrinsic genetic programs, cell‐autonomous senescence, and extrinsic environmental exposures, primarily ultraviolet radiation [227]. Research indicates that the skin microbiome is not a passive bystander reflecting host age; rather, it exhibits predictable compositional and functional trajectories with aging [229]. These skin‐MA signatures are intricately woven into host skin barrier properties, local and systemic immune states, and visible aging phenotypes, forming a complex bidirectional regulatory network [21]. On one hand, host aging‐related physiological changes (e.g., reduced sebum, immunosenescence) reshape the cutaneous microenvironment, driving microbial succession [230]. On the other hand, microbial dysbiosis and functional impairment can feedback onto the host via mechanisms involving epithelial barrier integrity, innate and adaptive immunity, and bioactive metabolite production, thereby accelerating or modifying local and systemic aging processes [231]. Thus, the skin microbiome functions as a critical interface and regulatory hub connecting intrinsic aging, environmental exposures, and aging phenotypes. The assembly and evolution of the skin microbiome occur throughout the host lifecycle, dynamically shaped by sequential biological transitions, including initial colonization, pubertal development, adult homeostasis, and physiological decline in elderly (Figure 8).

Skin microbial establishment begins at birth. Delivery mode dictates the initial microbial “seeding”: vaginally born neonates acquire microbiota resembling maternal vaginal flora (rich in *Lactobacillus* and *Prevotella*), whereas cesarean‐delivered infants acquire skin‐associated microbes like *Staphylococcus, Corynebacterium*, and *Propionibacterium* [182, 232]. The microbiome is also increased risks of immune‐mediated diseases like atopic dermatitis [233]. During infancy, the skin microbiome is relatively simple, with lower diversity than adults. Diversity gradually increases with growth, converging toward the adult configuration. Puberty marks a critical period of significant skin microbiome restructuring. Surging sex hormones stimulate sebaceous gland activity, creating favorable conditions for the proliferation of lipophilic microbes like *Cutibacterium acnes* (formerly *Propionibacterium acnes*) [234]. This stage witnessed dramatic shifts in diversity and composition, initiating sexual dimorphism [235]. For instance, facial *Cutibacterium acnes* abundance increases markedly in adolescents [236]; over‐proliferation of specific strains and the production of pro‐inflammatory metabolites (e.g., *porphyrins*) form the core microbial basis of acne pathogenesis. Following puberty, the skin microbiome enters a relatively stable adult plateau. The healthy adult skin flora exhibits high individuality and site‐specificity. Notably, *Lactobacillus* and *Cutibacterium* relative abundance decline significantly with age across all skin sites (forearm, buttock, face) [237]. Younger individuals harbor higher abundances of specific streptococci (e.g., *Streptococcus infantis, Streptococcus thermophilus*), whose secreted factors upregulate genes related to skin structure and barrier function, promoting collagen and lipid synthesis, thereby improving elasticity and hydration [85]. Core taxa maintain dynamic equilibrium through competition and cooperation, contributing to the acidic skin surface pH, defense against pathogens, and education of the local immune system.

In old age, the skin undergoes physiological alterations: sebaceous gland atrophy, reducing sebum, decreased stratum corneum hydration, slowed epidermal turnover, and progressive immunosenescence [231]. These changes drive a characteristic ecological succession in the microbiome [83]. Studies suggest that α‐diversity (richness and evenness) often increases in aged skin [237], potentially due to microenvironmental homogenization and weakened dominance of certain commensals. Compositionally, symbiotic microbiota associated with barrier health, such as specific *Lactobacillus* and *Cutibacterium* species, may decline [238], while opportunistic pathogens (e.g., certain *Staphylococcus* species) may increase in detection frequency [231, 239]. Functionally, the metabolic profile shifts. Metagenomic analyses indicate enhanced activity in microbial pathways related to cobalamin (vitamin B12) and heme biosynthesis in aged skin, correlating with the local inflammatory microenvironment and tissue remodeling [240]. Furthermore, the overall microbial metabolism may shift from utilizing sebum components to alternative substrates like desquamated keratinocyte proteins, potentially altering the skin's chemical environment and barrier properties [86].

#### Systemic regulatory networks: The multidimensional roles of the skin microbiome in aging

**Figure 9:**
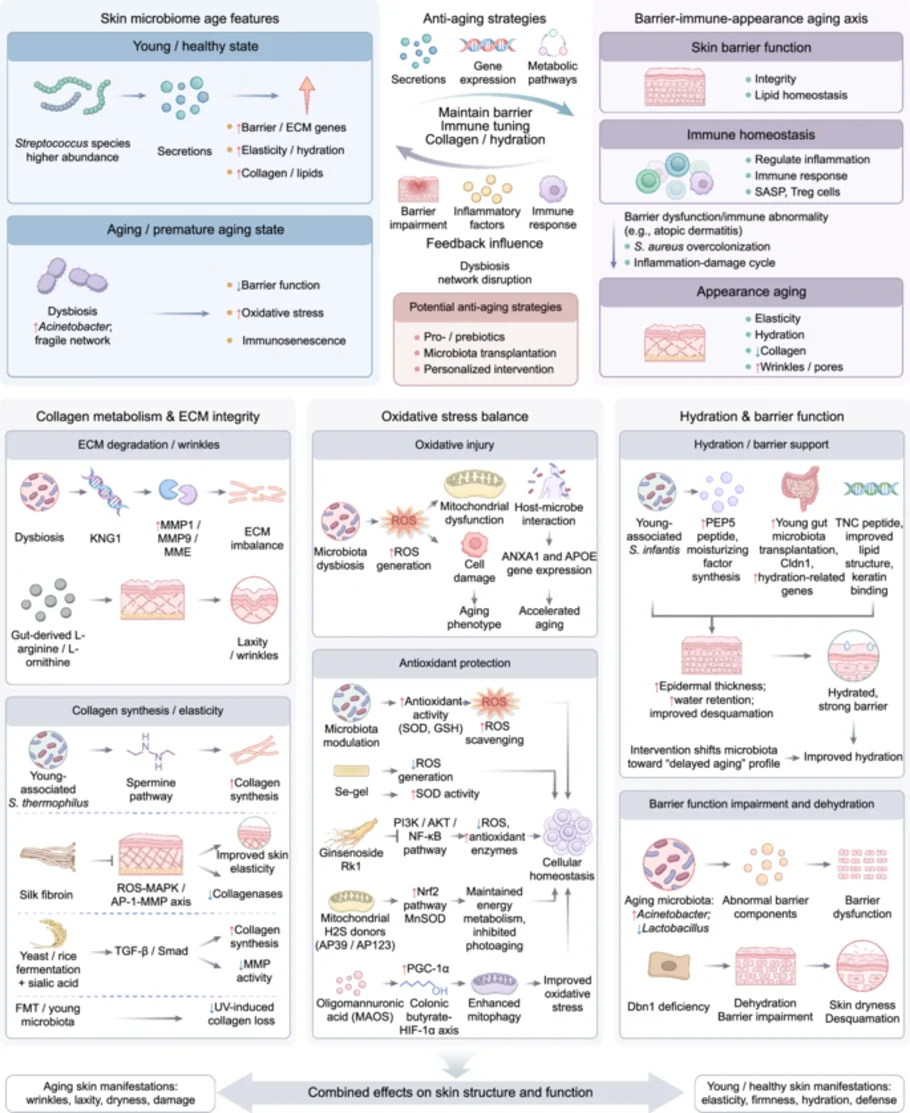
Bidirectional regulation between skin microbiota age and the skin barrier‐immune‐appearance aging axis. In young, healthy skin with high abundance of specific *Streptococcus* species (*Streptococcus infantis*, *S. thermophilus*), microbial metabolites maintain skin barrier integrity, immune homeostasis, and a youthful appearance by modulating the expression of genes governing skin structure and barrier function, thereby promoting collagen and lipid synthesis. Conversely, in aged or prematurely aged skin, impaired barrier function, oxidative stress, and immunosenescence drive skin‐MA dysbiosis, which in turn exacerbates barrier damage and inflammatory responses to establish a pathogenic vicious cycle. This bidirectional host‐microbiome interplay supports the potential of targeted skin microbiome modulation as an emerging strategy for healthy skin aging intervention.

**Figure 10:**
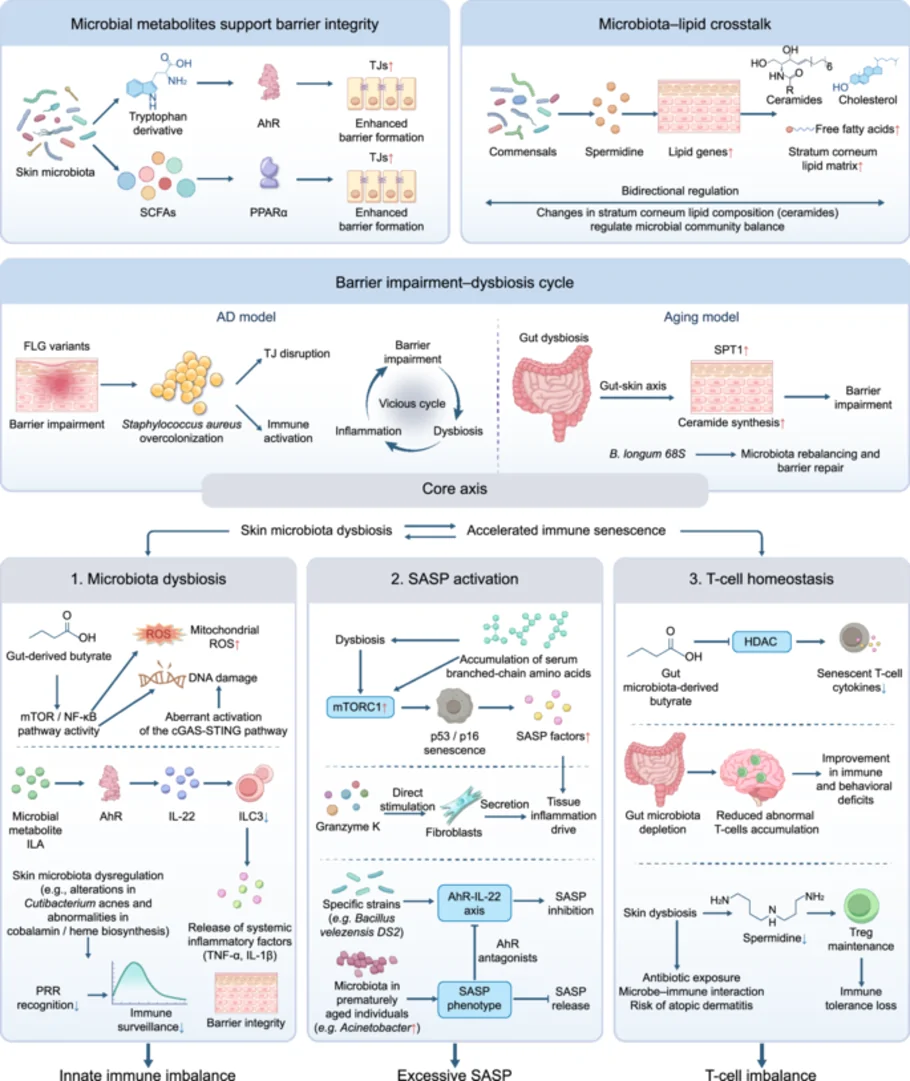
Skin microbiome metabolites regulate epidermal barrier homeostasis and contribute to immune senescence. Top panel: Microbial metabolites (tryptophan catabolites/indoles, SCFAs) activate aryl hydrocarbon receptor (AhR) and PPARα to upregulate tight junction (TJs) proteins. Commensals (e.g., *Streptococcus* spp.) bidirectionally regulate stratum corneum lipid synthesis—enhancing ceramides, cholesterol, and fatty acids via lipid‐related genes—with lipid composition reciprocally shaping microbial balance. Middle panel: Barrier dysfunction (e.g., FLG mutations in atopic dermatitis) drives dysbiosis (*Staphylococcus aureus* overcolonization), exacerbating impairment via immune activation and inflammation. In aging, gut dysbiosis and reduced SPT1 cause ceramide deficiency; *Bifidobacterium longum* 6BS intervention restores homeostasis and barrier function. Bottom panel: A mechanistic network links skin dysbiosis to immune senescence via three pathways: (1) metabolic reprogramming (altered butyrate/ROS/IL‐22 signaling impairs mTOR/NF‐κB/AhR and innate surveillance); (2) SASP induction (mTORC1 activation/DNA damage/AhR‐IL‐22 inhibition drives IL‐6/IL‐8 secretion); and (3) T cell modulation (gut‐derived HDAC inhibitors and skin dysbiosis affect pro‐inflammatory polarization, thymic output, and Treg maintenance). Through these interconnected mechanisms, the skin microbiome emerges as an active regulator of cutaneous immune aging and systemic inflammatory homeostasis.

The skin microbiome participates in cutaneous and systemic aging through a spectrum of direct and indirect mechanisms, primarily modulating barrier function, immune homeostasis, and tissue architecture (Figures 9 and 10).

The intact skin barrier is the primary defense against xenobiotics and water loss. Commensal microbes actively maintain this barrier through metabolic and signaling functions. Symbionts ferment sebum and sweat components to produce SCFAs and ceramide precursors [241]. Commensal skin microbes contribute to maintaining the acidic skin surface pH (~5.5) through the production of SCFAs and lactic acid, which collectively inhibit pathogen colonization and regulate epidermal desquamation [242]. Microbial metabolites act as signaling molecules, activating receptors on keratinocytes—such as the Aryl Hydrocarbon Receptor (AhR) and Peroxisome Proliferator‐Activated Receptors (PPARs)—to upregulate epidermal differentiation markers (e.g., filaggrin, loricrin) and tight junction protein [243, 244]. For instance, *Staphylococcus* epidermidis‑derived SCFAs promote the synthesis of barrier lipids via PPAR activation [244, 245, 246]. Additionally, specific streptococcal secretions upregulate genes related to collagen synthesis and lipid metabolism, indirectly supporting the dermal‐epidermal junction integrity [85]. The disorderly feedback loop: barrier disruption (e.g., filaggrin mutations in atopic dermatitis) alters the microenvironment, promoting *Staphylococcus aureus* over growth [247]. These pathogens secrete proteases and toxins that further degrade the barrier and trigger intense inflammation, establishing a “Barrier Damage‐Dysbiosis‐Inflammation” vicious cycle. During aging, a similar but more insidious cycle progressively erodes the barrier reserve function.

The skin is a critical immune organ. The microbiome acts as a constant educator and regulator of local immunity. Early‐life microbial colonization programs the functional maturation of SALT‐resident immune subsets, as exemplified by monocyte‐dependent tuning of IL‐1 signaling in neonatal skin [248]. Commensals interact with Pattern Recognition Receptors (PRRs) on keratinocytes and Langerhans cells, calibrating the immune response threshold to defend against pathogens while maintaining tolerance to commensals. In aging, a bidirectional detrimental relationship emerges. Immunosenescence (reduced T‐cell reactivity, blunted innate responses) weakens control over the microbiota, permitting the expansion of potentially harmful taxa [249]. Meanwhile, dysbiosis exacerbates immune aging. Altered metabolite profiles can disturb the local cytokine balance (e.g., IL‐1β, IL‐10) [250]. Furthermore, the skin microbiome may influence systemic immune tolerance via modulating regulatory T cell (Treg) function [85, 251]. Thus, cutaneous ecological disruption is both a manifestation and a driver of systemic immunosenescence. Structural aging manifests as the degradation and loss of dermal collagen and elastic fibers, resulting in wrinkles and laxity. While UV radiation is the primary extrinsic driver [227], the microbiome modulates the microenvironment affecting extracellular matrix (ECM) turnover. Microbial activity can influence Matrix Metalloproteinase (MMP) activation. For example, the secretions of *Streptococci* may be related to the structure and barrier function of the aging skin. [85]. In physiological aging, microbial metabolites may alter fibroblast activity or modulate the oxidative stress response, impacting ECM homeostasis [238]. Certain metabolites exhibit protective effects. For instance, silk protein (SF) can suppress ROS‐MAPK‐AP‐1‐MMP signaling, reducing collagenase expression [252]. Yeast/rice fermentation filtrates promote collagen synthesis via the TGF‐β/Smad pathway while downregulating inflammatory cytokines to inhibit MMP activity [253]. Conversely, overabundance of specific pathogens or their metabolites (e.g., porphyrins from *Cutibacterium acnes* generating ROS under UV light) may exacerbate photo‐damage. Although direct evidence is still accruing, the interaction network between the skin microbiome, dermal fibroblasts, and immune cells is undeniably a critical determinant of structural integrity.

Based on the aforementioned mechanisms, modulating the cutaneous micro‐ecosystem has emerged as a frontier in dermatology and anti‐aging therapeutics. These strategies can be categorized into three domains: maintenance, modification, and systemic intervention. The principle of “Do No Harm”. The core objective is preserving the existing exfoliation. It is recommended to use mild cleansers to avoid over‐cleansing and excessive exfoliation, thereby protecting the acid mantle and commensal flora [254, 255, 256]. Skincare formulations should be selected based on pH compatibility and ingredient profiles that support microbial viability. Furthermore, stringent photoprotection remains the cornerstone of preventing photo‐aging and may stabilize the micro‐ecosystem by minimizing UV‐induced damage to both host cells and resident microbes [257]. Active Bio‐Augmentation. Modification strategies aim to actively introduce beneficial components: (1) Prebiotics: utilizing ingredients (e.g., specific oligosaccharides) selectively metabolized by beneficial taxa to promote their proliferation. (2) Probiotics: topical application of viable microorganisms (e.g., specific strains of *Lactobacillus, Staphylococcus epidermidis*) to reinforce colonization resistance. (3) Postbiotics: employing non‐viable bacterial cells, cell fragments, or soluble metabolites (e.g., fermentation filtrates, SCFAs). Their advantages lie in defined mechanisms of action and superior formulation stability. (3) Microbiome transplantation: transferring skin microbiota from healthy donors to affected sites has shown promise in recurrent atopic dermatitis, though standardization and long‐term safety profiles require further evaluation. Considering the gut–skin axis, systemic interventions via diet or oral probiotics can positively modulate skin physiology [228]: (1) Promoting humectant synthesis: high‐abundance *Streptococci* in younger individuals (e.g., *Streptococcus infantis*) upregulate genes associated with skin hydration, improving water retention and desquamation [85]. Similarly, the AAQPR peptide (PEP5) upregulates hydration‐related gene expression in keratinocytes, clinically validating its efficacy in enhancing skin moisture content [258]. (2) Enhancing Barrier Lipid Architecture: functional collagen mimetic peptides, such as TNC, significantly improve epidermal thickness and hydration by optimizing lipid organization, binding to keratin, and modulating hydration dynamics [259]. (3) Regulating hydration gene expression: FMT from young mice upregulates Dbn1 expression in aged mouse dermis, enhancing stratum corneum thickness and hydration capacity; conversely, Dbn1 deficiency leads to barrier dysfunction and dehydration [260]. Furthermore, the vulnerability of the aged skin microbiome can be mitigated by interventions that shift the community toward a “delayed‐aging” profile, thereby improving skin moisture [238]. Cross‐sectional studies corroborate that shifts in the skin microbiome (e.g., reduced *Lactobacillus*) correlate with levels of natural moisturizing factors and lipids, suggesting a direct microbial role in hydration regulation [237].

With deeper insights into individual microbiome signatures, integrating multi‐omics data (genomics, metabolomics) will enable the design of precision dermatology and personalized anti‐aging regimens.

### The genitourinary microbiome: Aging dynamics of a partner‐shared ecosystem

Reproductive aging constitutes a core and complex dimension of organismal senescence, characterized by the progressive decline of gonadal function, diminished gamete quality, and dysregulation of endocrine networks [261, 262]. In females, this process is demarcated by the irreversible depletion of ovarian reserve, culminating in menopause [263, 264]. In males, it manifests as a gradual decline in serum testosterone, impaired spermatogenesis, and recession of associated reproductive parameters [265, 266]. Globally, the trend toward delayed childbearing and the rising incidence of age‐related infertility [267] underscore the profound public health challenge posed by reproductive aging [268, 269]. Furthermore, the prolonged estrogen deficiency following menopause is intimately linked to an increased risk of systemic aging phenotypes, including cardiovascular disease, osteoporosis, and cognitive decline [264, 270]. Classical reproductive biology has traditionally focused on cell‐autonomous aging mechanisms within the gonads, such as the attenuation of the hypothalamic‐pituitary‐gonadal (HPG) axis [271, 272] and accelerated follicular atresia or seminiferous epithelium degeneration [261]. However, this organ‐centric or axis‐centric perspective fails to fully account for the extensive and intimate associations between reproductive aging and systemic health. Recent breakthroughs in microbiome science have catalyzed the emergence of the “microbiome‐reproduction axis” as a novel research paradigm. This framework reveals that symbiotic microbial communities residing in multiple body sites—particularly the gut and reproductive tract—are not passive inhabitants. Instead, they dynamically and profoundly participate in regulating reproductive homeostasis through metabolites, immunomodulation, and epigenetic modifications [273, 274].

This paradigm is well‐established in female reproductive health, where the *Lactobacillus*‐dominated, acidic vaginal environment is a recognized hallmark of health [275]. Contemporary research further reveals the vaginal microbiome as a dynamic ecosystem integrating microbes, local anatomy, endocrine signaling, and the immune system—its stability is paramount for immune tolerance, infection prevention, and favorable pregnancy outcomes [276, 277]. In contrast, research on the male genitourinary microbiome is nascent. Nevertheless, high‐throughput sequencing has confirmed that sites such as the testes, epididymis, and semen are not sterile [278] but host site‐specific microbial communities [279]. Preliminary evidence links these communities to semen quality parameters, though causal mechanisms await elucidation.

Crucially, the male and female reproductive microbiomes do not evolve in isolation. Through close contact, including sexual intercourse, partners engage in continuous microbial exchange, mutually shaping each other's ecosystems [280, 281, 282]. This forms a functionally coupled “partner‐shared micro‐ecosystem” [283]. This systemic view necessitates a shift from traditional “single‐sex” or “single‐individual” frameworks toward an integrated “dyadic” or “ecological pair” analysis. Consequently, reproductive aging can be reinterpreted as the synergistic functional decay of this shared ecosystem.

#### Lifecycle trajectories and partner‐shared dynamics of the genitourinary microbiome

**Figure 11:**
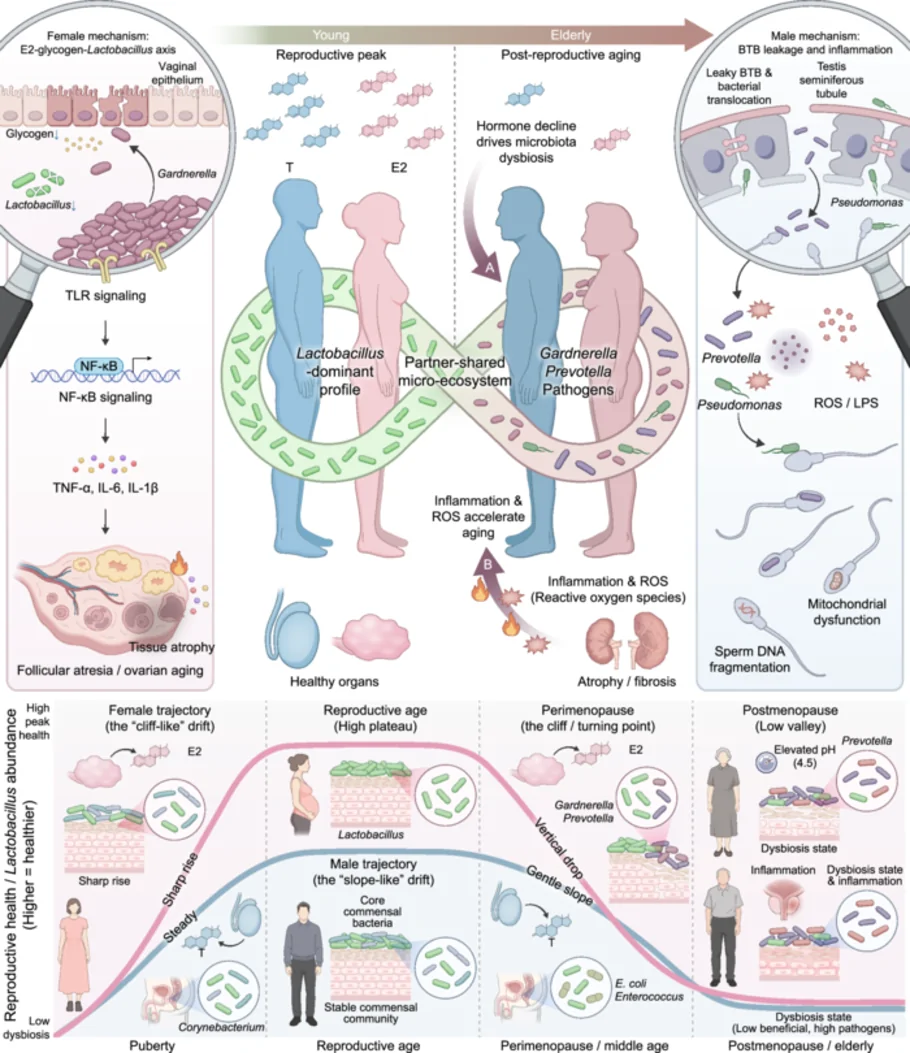
Age‐dependent evolution of the reproductive tract microbiota in both sexes and gonadal aging mechanisms driven by the partner‐shared microecosystem. Top panel: This panel illustrates the vicious hormone–microbiota–inflammation cycle driving gonadal aging via the partner‐shared microecosystem. Declining sex hormones in both sexes shift the reproductive tract microbiome from *Lactobacillus*‐dominated homeostasis to dysbiosis with opportunistic pathogens (*Gardnerella*, *Prevotella*, *Pseudomonas*). In females, estrogen (E2) depletion triggers an “estrogen‐glycogen‐inflammation” cycle that activates TLR/NF‐κB signaling to accelerate ovarian senescence. In males, Testosterone (T) disrupts the blood‐testis barrier (BTB), with LPS inducing ROS to impair spermatogenesis. Reciprocal amplification of dysbiosis, inflammation, and oxidative stress forms a vicious cycle driving reproductive and systemic aging, highlighting reproductive aging as an inter‐partner microbially coupled process rather than a single‐organ event. Bottom panel: This panel maps the sexually dimorphic lifetime trajectory of the reproductive tract microbiome, with the horizontal axis as life stages and the vertical axis as reproductive health abundance. Females exhibit a puberty‐related steep rise, reproductive‐age *Lactobacillus*‐dominated plateau, and perimenopausal (~45 years) cliff‐like decline into persistent post‐menopausal dysbiosis. Males show a gradual puberty‐related rise, reproductive‐age stability, and middle‐age slow drift toward pro‐inflammatory dysbiosis with *E. coli* and *Enterococcus* enrichment. Sex hormones drive these distinct trajectories, which converge on an aging‐related pattern of beneficial bacteria loss and pathogenic enrichment.

The assembly and evolution of the genitourinary microbiome is a dynamic process dominated by sex hormones, exhibiting pronounced sexual dimorphism and continuous interpersonal interaction (Figure 11).

The female reproductive microbiome, particularly the vaginal microbiota, evolves in tight coupling with estrogen fluctuations, displaying distinct life‐history stages. Pre‐puberty, the vaginal environment is neutral, harboring a simple community. Puberty activates the hypothalamic‐pituitary‐ovarian (HPO) axis, elevating estrogen levels, which stimulates vaginal epithelial proliferation and glycogen accumulation. Glycogen release and enzymatic hydrolysis provide substrates for fermentative bacteria like *Lactobacillus* [284]. These *Lactobacilli* produce lactic acid, maintaining a pH < 4.5 that inhibits pathogens, thereby establishing a low‐diversity, high‐stability community dominated by species like *Lactobacillus crispatus* and *Lactobacillus gasseri* [285]. Notably, Gajer et al. [286] demonstrated cyclical fluctuations: microbial diversity peaks during menstruation, with significant increases in *Fusobacteria, Proteobacteria*, and *Actinobacteria*, shifting towards a *Lactobacilli*‐dominant profile in the follicular and luteal phases, driven by cyclical estrogen changes. During reproductive years, this *Lactobacilli*‐dominant profile is maintained and further reinforced during pregnancy due to high estrogen status—a presumed protective adaptation for the fetus [287, 288, 289]. Perimenopause marks the decisive “tipping point.” Ovarian senescence causes an abrupt decline in estrogen, leading to vaginal atrophy and a sharp reduction in glycogen availability. This severely compromises the substrate and niche for *Lactobacilli* [29]. Consequently, *Lactobacilli* abundance plummets, while anaerobic (e.g., *Gardnerella*, *Prevotella, Mobiluncus*) and facultative anaerobe proportions surge [290]. Metagenomic analyses reveal a functional shift from carbohydrate fermentation to amino acid degradation [291]. In post‐menopause, the low‐estrogen state persists, further exacerbating dysbiosis. The proportion of lactobacilli‐dominated communities drops significantly [290, 292], facilitating colonization by opportunistic pathogens [133] and increasing the risk of Bacterial Vaginosis (BV) and Aerobic Vaginitis (AV) [293]. This establishes a vicious cycle of “Dysbiosis‐Inflammation‐Tissue Atrophy,” severely compromising urogenital health and quality of life in older women.

Research on the male genitourinary microbiome is comparatively nascent. Niches include the foreskin, coronal sulcus, urethra, seminal vesicles, prostate, and testes, each exhibiting spatial heterogeneity [294, 295]. Post‐puberty, androgen‐driven maturation of reproductive organs and active secretion from cutaneous glands (e.g., Tyson's glands) establish a relatively stable structure. For instance, the penile skin (coronal sulcus) is often dominated by skin‐associated *Corynebacterium* and *Staphylococcus* [296], while the urethral microbiome may contain *Lactobacillus* and *Streptococcus*. These core commensals help maintain local immune homeostasis [297]. Unlike the abrupt perimenopausal transition in females, male reproductive aging follows a “progressive ecological drift.” Starting in mid‐life (approx. 40–50 years), with a slow, continuous decline in serum total testosterone and systemic metabolic shifts (e.g., increased insulin resistance), the local microenvironment changes [298]. Studies suggest a gradual decline in certain core commensals and a slow rise in taxa associated with inflammation or disease (e.g., *Aerococcus, Prevotella*) [299]. This drift correlates with age‐related immunosenescence and chronic low‐grade inflammation [300, 301]. In elderly, microbial diversity may increase anomalously, stability decreases, and detection rates of potential pathogens (e.g., *Escherichia coli, Enterococcus*) rise. Clinically, this manifests as increased asymptomatic bacteriuria, chronic prostatitis/chronic pelvic pain syndrome (CP/CPPS), and benign prostatic hyperplasia [302, 303, 304]. Dysbiosis‐associated chronic inflammation and oxidative stress exert cumulative damage on the spermatogenic microenvironment, BTB integrity, and sperm function [305, 306, 307, 308] (e.g., DNA fragmentation).

Male and female reproductive microbiomes do not evolve in isolation; sexual intercourse creates a tightly coupled “partner‐shared micro‐ecosystem.” Studies confirm high similarity between stable partners' reproductive organs microbiomes, sharing substantial bacterial sequences. Sexual activity acts as a key medium for bidirectional microbial exchange and transplantation. For example, a woman's *Lactobacilli*‐dominant state may be temporarily altered post‐coitus; conversely, men partnered with BV‐positive women often exhibit altered penile microbiota, potentially serving as reservoirs for pathogens. This coupling has profound clinical implications. Treating recurrent BV in women alone often yields limited success due to “ping‐pong” transmission between partners. Randomized controlled trials demonstrate that synchronous treatment of the male partner significantly reduces female recurrence rates. This provides strong evidence that reproductive health—including age‐related dysbiosis—must be managed as an ecosystemic issue at the “dyadic unit” level. Ultimately, while the trajectories are “etiologically homologous” (both hormonally regulated) yet “phenotypically heterogeneous” (female tipping point and male drift), they converge through continuous interaction towards an “ecological aging” endpoint characterized by altered diversity, attenuated beneficial taxa, and increased potential pathogens.

#### Systemic regulatory networks: The dual‐layer model of microbiome‐driven reproductive aging

**Figure 12:**
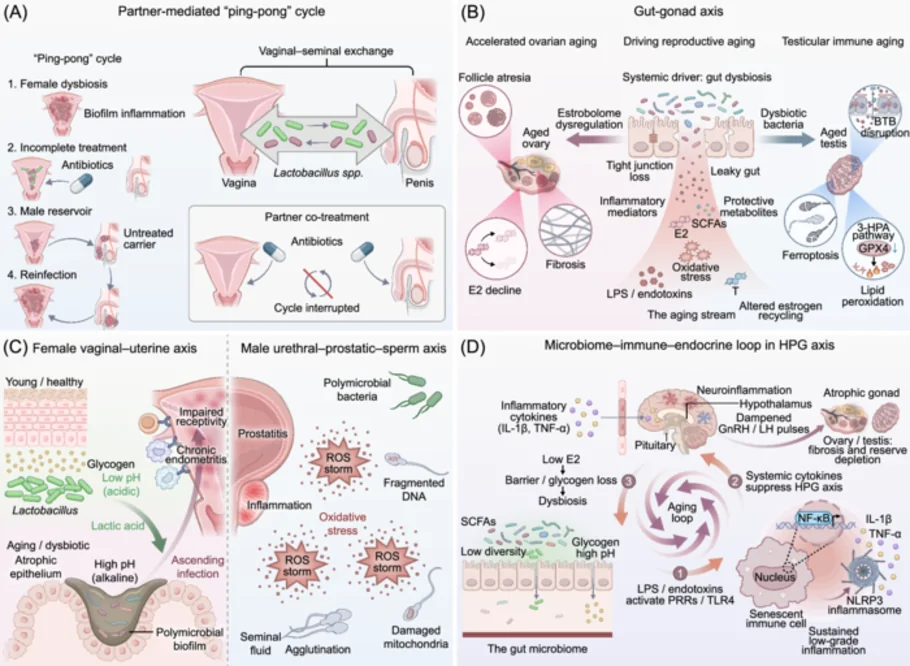
Integrated host–microbiome mechanisms underlying reproductive aging. (A) It illustrates potential microbial exchange between sexual partners within a shared reproductive microbial ecosystem, highlighting how persistent dysbiosis and recurrent bidirectional transmission may contribute to chronic reproductive tract inflammation and impaired reproductive health. (B) It summarizes the enteric‐gonadal axis, whereby gut barrier dysfunction, systemic inflammation, metabolic disturbance, and oxidative stress may influence gonadal aging through endocrine and immune pathways in a sex‐dependent manner. (C) It compares local reproductive tract homeostasis and dysbiosis in females and males, showing how microbial imbalance, barrier disruption, chronic inflammation, and oxidative stress contribute to reproductive tissue dysfunction and age‐associated functional decline. (D) It integrates these mechanisms into a broader immuno‐endocrine host‐microbiome network, in which dysbiosis, inflammatory activation, and hormonal dysregulation form interconnected self‐reinforcing feedback loops that may accelerate reproductive aging.

Genitourinary dysbiosis drives reproductive senescence through two interrelated layers: a systemic‐remote network and a local‐proximal network (Figure 12). The gut microbiome functions as a systemic regulatory hub, remotely influencing gonadal function via the “gut–gonad axis.” The core mechanism involves gut dysbiosis compromising barrier integrity, permitting the translocation of microbial products like LPS into circulation. This induces chronic, low‐grade systemic inflammation (metabolic endotoxemia) and metabolic dysregulation. This pro‐inflammatory, pro‐oxidative systemic milieu constitutes the “hostile soil” for reproductive aging [309, 310].

In Females: Systemic inflammation inflicts oxidative damage on ovarian granulosa cells, disrupting follicular development and steroidogenesis, potentially accelerating the activation and depletion of the primordial follicle pool [311, 312]. At the molecular level, NF‐κB acts as a central transcriptional hub. Upon sensing MAMPs, it drives sustained expression of pro‐inflammatory cytokines, forming a mutually reinforcing loop with inflammasomes (e.g., ASC/NLRP3) [313, 314]. This persistent inflammatory signaling “locks in” the inflammasome pathway, promoting structural abnormalities and functional exhaustion. Ovarian aging is characterized by increased immune cell infiltration and upregulated IL‐1β, TNF‐α, IL‐6, and NLRP3 expression. Critically, pharmacological inhibition of the ASC/NLRP3 inflammasome preserves a larger primordial follicle reserve in aged mice, suggesting a causal link between inflammation and reserve depletion [309]. Clinically, this manifests as chronic endometritis and compromised endometrial receptivity [315]. Furthermore, the gut microbiome modulates the “estrobolome”—the collection of microbial genes involved in estrogen metabolism [316]. Bacteria possessing β‐glucuronidase activity deconjugate estrogens in the gut, facilitating their reabsorption into circulation. Dysbiosis alters this enterohepatic circulation, impacting systemic estrogen levels, HPO axis feedback, and the ovarian microenvironment [317, 318].

In Males: Systemic inflammation and oxidative stress disrupt the blood‐testis barrier (BTB), altering testicular immune privilege and impairing Sertoli and Leydig cell function, thereby compromising spermatogenesis and testosterone synthesis [319]. Conversely, specific gut microbial metabolites (e.g., 3‐hydroxyphenylacetic acid) may exert protective effects on spermatogenesis via antioxidant pathways in aging models [320, 321]. Thus, the gut‐gonad axis establishes a distal regulatory pathway linking gut ecological health to systemic metabolic‐inflammatory status and gonadal maintenance.

Crucially, this inflammation is not confined to peripheral tissues. Systemic low‐grade inflammation can intercept central nervous system pathways, modulating HPG axis activity. Pro‐inflammatory cytokines can alter GnRH pulse frequency and LH secretion rhythms, thereby disturbing sex hormone feedback homeostasis [322, 323]. The local microbiota exerts direct effects within target organ microenvironments [324, 325].

In the Female Vagina: a healthy, *Lactobacilli*‐dominant state maintains the defensive barrier via acid production, bacteriocin secretion, and competitive exclusion [326, 327]. During dysbiosis, the shift towards an anaerobic, diverse community enables opportunistic pathogens to form biofilms and produce cytotoxins (e.g., putrescine) and proteases. These directly disrupt epithelial tight junctions and weaken the physical barrier. Concurrently, MAMPs from dysbiotic communities activate PRRs on epithelial and immune cells, sustaining a local pro‐inflammatory environment. This inflammation can ascend to the endometrium, causing chronic endometritis and impairing receptivity, or affect the fallopian tube microenvironment, compromising gamete function and early embryonic development [328].

In the Male Genitourinary Tract: dysbiosis is closely linked to chronic prostatitis and vesiculitis [329, 330]. Locally colonized pathogens can directly impair sperm motility via adhesion and agglutination [331]. More importantly, the induced chronic inflammation leads to massive ROS production, causing sperm membrane lipid peroxidation, mitochondrial dysfunction, and DNA fragmentation, thereby significantly degrading semen quality [332, 333]. The local “Dysbiosis—Chronic Inflammation—Oxidative Stress—Tissue/Cell Damage” cascade represents the proximal, direct mechanism by which the microbiome impacts reproductive outcomes.

A sophisticated bidirectional regulatory loop exists between sex hormones and the microbiome. Estrogen promotes vaginal epithelial maturation and glycogen deposition, providing substrates for lactobacilli and maintaining the acidic niche. Conversely, the postmenopausal estrogen decline drives the shift towards a diverse, anaerobic community. Similarly, gut microbiome composition shifts reproducibly before and after menopause [334, 335], with some studies reporting a decline in butyrate‐producing taxa and functional gene abundance post‐cessation [336]. This suggests a chain reaction: “Hormone Decline—Microbial Functional Drift—Elevated Inflammatory Threshold.” Local estrogen therapy restores *Lactobacilli*‐dominance [337], confirming the niche‐shaping role of hormones. The reverse pathway is equally critical. Microbial imbalance and the resultant chronic inflammation can desensitize hormone receptor signaling, rendering tissues functionally hypo‐responsive even under normal hormone exposure [338]. Simultaneously, altered gut microbial β‐glucuronidase activity modifies enterohepatic circulation, potentially reducing bioavailable circulating estrogen and amplifying postmenopausal hypo‐estrogenic effects [335]. Thus, sex hormones and the microbiome form a closed‐loop feedback system [339]. Overall, aging is often accompanied by declining microbiome diversity and rising metabolic‐inflammatory risk, making the “Microbiome—Inflammation—Endocrine” network prone to self‐perpetuating closed‐loop states. This locks what might otherwise be reversible niche fluctuations into a trajectory of sustained reproductive functional decline.

To sum up, anti‐aging interventions targeting the microbiome should pivot from “structural correction” to “functional anti‐aging.” The primary translatable targets are the functional outputs of the microbiota and the systemic host milieu they shape, rather than merely altering taxonomic ratios [340]. Guiding principles include: (1) Prioritizing the mitigation of upstream pro‐aging backgrounds to reduce long‐term driving effects [340, 341]; (2) Maintaining local niche stability and immune tolerance to minimize chronic inflammation‐mediated remodeling of the reproductive microenvironment [342]; (3) Adopting functional endpoints—such as inflammatory markers, oxidative stress, barrier permeability, and hormonal responsiveness—as core outcomes [343].

Tier 1: Primary Prevention—Diet and Lifestyle Optimization. The foundation lies in modulating the gut‐gonad axis via diet. Fermentable dietary fiber enhances microbial fermentation, boosting SCFAs—particularly butyrate—production. SCFAs support epithelial energy homeostasis and bolster barrier function through receptor signaling and immune‐metabolic crosstalk, promoting T‐regulatory cell induction and lowering the threshold for chronic low‐grade inflammation. This provides a more stable immuno‐metabolic backdrop for reproductive tissues [342, 344]. Clinical evidence links higher fiber intake to improved SCFAs levels and favorable shifts in inflammatory biomarkers [345, 346]. Nutritional factors intersecting with the microbiome and hormones must also be considered; for instance, the bioavailability of isoflavones depends on specific bacterial conversion capacities [347, 348]. Furthermore, the perimenopausal estrogen decline precipitates lactobacilli‐dominant dysbiosis; local estrogen therapy correlates with niche restoration and pH normalization, suggesting a complementary strategy of “nutritional support plus judicious local hormonal optimization” [337, 349].

Tier 2: Secondary Intervention—Microbial Therapeutics for Niche Restoration. Secondary strategies target individuals exhibiting persistent inflammatory phenotypes, recurrent infections, or overt dysbiosis. Here, microbial therapies (probiotics, prebiotics, synbiotics) must aim for “niche restoration” rather than mere bacterial supplementation. The goal is to re‐establish stable colonization and functional recovery, using the downregulation of chronic inflammation as the clinical endpoint [350, 351, 352, 353]. Live Biotherapeutic Products (LBPs) offer a more controllable path via strain‐specific effects for precise niche reconstruction [354]. Given the heterogeneity of male studies and the challenges of low‐biomass systems, emphasis must be placed on functional endpoints (ROS, SDF, lipid peroxidation, cytokine profiles) rather than taxonomic composition alone. Rigorous absolute quantification and contaminant controls are essential to strengthen causal inference [355].

Tier 3: Tertiary Intervention—Ecosystem Reset and Systemic Reprogramming. Tertiary strategies involve ecosystem‐level resetting for cases of severe dysbiosis, refractory chronic inflammation, or significant metabolic‐inflammatory burden. Such interventions represent “ecosystem‐scale manipulation” and must be governed by strict indications, ethical oversight, and long‐term safety monitoring to prevent unwarranted generalization [356, 357]. The rationale is to reprogram downstream organ microenvironments via wholesale community transfer. Animal models suggest that FMT from young donors ameliorates aging‐associated inflammation and metabolic phenotypes, supporting the plasticity of the aging trajectory via the microbiome [358, 359]. While safety frameworks exist for specific diseases, FMT remains exploratory for reproductive aging. Beyond reducing recurrence risk, its potential value lies in restoring a lactobacilli‐dominant niche to dampen persistent mucosal inflammation and barrier disruption, thereby slowing the time‐accumulated effects of the “inflammation‐remodeling‐decline” cascade. However, this requires validation via randomized controlled trials and biomarker‐stratified analyses to avoid over‐extrapolating niche restoration as a universal anti‐aging panacea [360, 361].

Reproductive aging and recurrent inflammation exhibit strong network properties, influenced by the partner's microbiome. Unilateral intervention while the partner remains dysbiotic risks creating a cycle of re‐exposure, re‐destabilization, and relapse. Therefore, partner‐synced management is essential [362]. Randomized controlled trial evidence confirms that synchronous treatment of male partners significantly reduces female recurrence rates in Bacterial Vaginosis, substantiating the “partner‐synced intervention” strategy [363]. Longitudinal and metagenomic studies further illustrate that sexual activity shapes the shared micro‐ecosystem, influencing imbalance risk [364]. Consequently, in cases of recurrent infection, persistent inflammation, or adverse reproductive outcomes, a “synchronized intervention, joint follow‐up” model is warranted. Integrating micro‐ecological assessment into the dyadic framework is crucial for severing cross‐transmission and re‐exposure chains.

However, current evidence regarding the male reproductive microbiome and aging remains relatively limited and is largely based on observational or correlational studies rather than direct mechanistic validation. Reported associations between microbial alterations and aging‐related reproductive dysfunction may be influenced by multiple confounding factors, including hormonal status, sexual behavior, lifestyle, medication exposure, comorbidities, and sampling variability. Therefore, causal interpretations should be made cautiously, and further longitudinal and mechanistic studies are required to establish definitive biological links.

## “INFLAMMAGING”: THE INTEGRATIVE NETWORK OF MULTI‐NICHE MICROBIOME‐DRIVEN SYSTEMIC INFLAMMATION AND AGING

The biological essence of aging lies in the progressive loss of homeostasis, manifesting through diverse and complex phenotypes. Among the hallmarks of aging, “inflammaging”—a chronic, low‐grade, non‐infectious, systemic inflammatory state—has been established as a core pathophysiological driver of aging processes and numerous age‐related diseases [1, 58]. In the early 21st century, Franceschi et al. [200] formally defined inflammaging as the chronic, low‐level activation of the innate immune system, characterized by persistently elevated circulating levels of pro‐inflammatory cytokines (e.g., IL‐6, TNF‐α) and acute‐phase reactants (e.g., CRP). Its core feature is a sustained, systemic inflammation without overt infection, serving as a key mechanism driving atherosclerosis, neurodegeneration, insulin resistance, and other age‐related pathologies [309].

We have systematically discussed in the previous sections that the age‐related trajectories of the gut, oral, skin, and genitourinary microbiomes and their localized impacts. However, a more integrative scientific question emerges: How do these widely distributed, heterogeneous local microbial dysbioses collectively shape the systemic state of the host? Converging evidence strongly indicates that the induction of inflammaging is their shared terminal effector pathway. Microbial dysbiosis at individual niches does not merely cause isolated local lesions; rather, through a series of highly convergent molecular and cellular mechanisms, it amplifies, converges, and systematizes local disruptive signals, ultimately sculpting the host's systemic inflammatory aging phenotype. Consequently, inflammaging can be conceptualized as the “effector integrator” and “phenotypic amplifier” through which the microbiome exerts its influence on aging.

### The cooperative network mechanism: Multi‐niche microbial dysbiosis driving inflammaging

**Figure 13:**
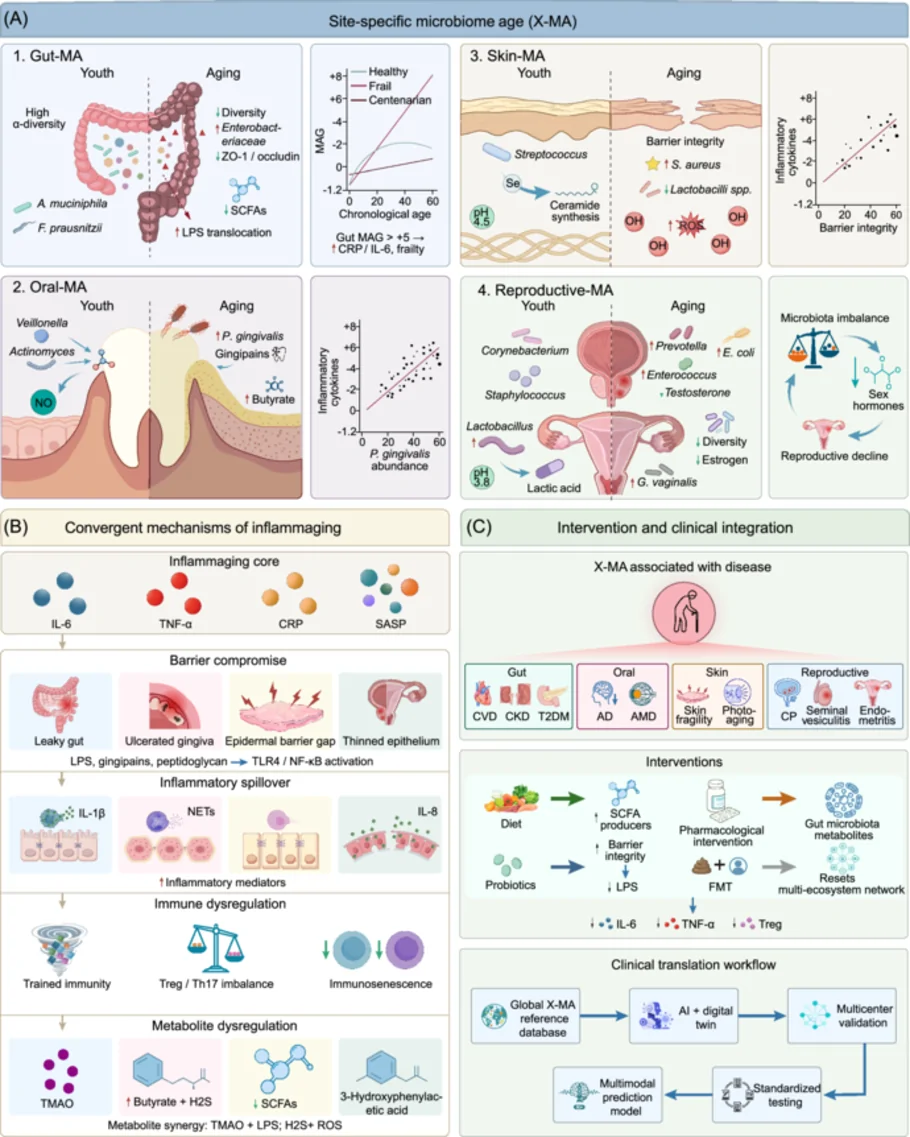
X‑MA as a multidimensional readout of inflammaging. The figure depicts the X‑MA model across four human niches (gut, oral, skin, reproductive tract). (A) Age‑related shifts in microbial composition, barrier integrity, and niche‑specific aging trajectories (gut‐MA, oral‐MA, skin‐MA, reproductive‐MA). A positive X‑MA gap indicates deviation from healthy aging and correlates with elevated inflammatory markers. (B) Four convergent pathways to inflammaging‐barrier destruction, local inflammation spillover, immune dysregulation, and metabolite dysregulation‐collectively driving chronic elevation of IL‑6, TNF‑α, CRP, and SASP. (C) Associations of X‑MA with major age‑related diseases, such as cardiovascular diseases (CVD), chronic kidney disease (CKD), type 2 diabetes mellitus (T2DM), Alzheimer's disease (AD), age‐related macular degeneration (AMD), skin fragility, chronic prostatitis (CP), and potential interventions (diet, probiotics, pharmacological agents, FMT) that reduce systemic inflammation. A clinical translation workflow (global database, AI/digital twins, multi‑center validation, standardized testing, multimodal prediction) is outlined.

Although inhabiting distinct anatomical sites, the gut, oral, skin, and genitourinary microbiomes converge upon shared pathways during aging. Their collective dysbiosis orchestrates inflammaging through four interlocking mechanistic layers, constituting a stereoscopic mechanistic network (Figure 13).

#### Barrier compromise and persistent microbial antigen translocation

Intact biological barriers are the primary safeguard for immune tolerance. Dysbiosis in all four niches directly or indirectly erodes barrier integrity [152]. Dysbiosis disrupts tight junctions, causing “leaky gut” and permitting the translocation of MAMPs, such as LPS, into the portal circulation [152]. Periodontitis creates ulcerated pockets, providing a direct conduit for oral bacteria and virulence factors (e.g., *Gingipains*) to enter the bloodstream [184]. Aged skin exhibits diminished epidermal barrier function, exacerbated by dysbiosis (e.g., *Staphylococcus aureus* growth) [231]. Post‐menopausal atrophy and dysbiosis thin the vaginal epithelium, compromising its defensive capacity [365]. Despite variations in antigen load, these niches collectively expose the host to low‐level, chronic microbial antigens. This persistent antigenemia acts as a chronic stimulant to the innate immune system, initiating and sustaining systemic low‐grade inflammation.

#### Focal inflammatory niches and systemic mediator “spillover”

Microbial dysbiosis establishes chronic inflammatory foci within local tissues. These act as distributed “inflammatory generator,” continuously seeding pro‐inflammatory mediators into the systemic circulation. Dysbiosis activates mucosal immunity, driving cytokine production. Periodontal tissues are highly active inflammatory sites, releasing high levels of IL‐1β, IL‐6, and TNF‐α [198]. Hajishengallis et al. [197] demonstrated that repetitive low‐grade endotoxemia induces “trained myelopoiesis,” epigenetically reprogramming myeloid progenitors to create an “inflammatory memory” that amplifies subsequent responses. Chronic dermatoses (e.g., psoriasis) and genital infections (e.g., BV, endometritis) sustain local inflammatory milieus [86, 328]. The cytokines and chemokines generated are not confined locally; they “spill over” via lymphatics and venules into systemic circulation [152]. The cumulative spillover from multiple foci elevates baseline plasma pro‐inflammatory mediator levels, directly shaping the cytokine profile of inflammaging [152].

#### Chronic immune activation, maladaptive training, and functional exhaustion

Persistent antigenic stimulation and inflammatory mediator exposure induce profound, age‐related immune remodeling. Continuous MAMPs exposure keeps innate immune cells (monocytes/macrophages) in a state of “para‐inflammation,” characterized by heightened basal reactivity and tonic low‐level cytokine secretion [150]. Dysbiosis‐induced depletion of SCFAs producers (e.g., butyrate) impairs T‐regulatory cell function and promotes pro‐inflammatory Th17 skewing—a mechanism likely conserved across mucosal sites [39, 151]. Chronic activation leads to immune cell exhaustion, accelerated replicative senescence, and overall blunted responsiveness. This weakens microbial control, creating a vicious cycle: Dysbiosis—Immune Activation—Immunosenescence—Further Dysbiosis [366]. Mechanistically, *Porphyromonas gingivalis* invasion of dendritic cells can activate the SASP, releasing SASP‐exosomes that transmit senescence signals to bystander cells, crippling antigen presentation and fueling inflammaging.

#### Metabolic dysregulation and accumulation of pro‐aging metabolites

The microbiome serves as an extension of host metabolism. Aging‐associated dysbiosis shifts the metabolic profile from beneficial to detrimental [249]. A shift towards proteolytic fermentation produces cytotoxic metabolites like hydrogen sulfide, indoxyl sulfate, and p‐cresol [196]. Dysbiosis enhances catabolism, generating tissue‐destructive butyrate (in the oral context) and hydrogen sulfide [196]. Altered microbial metabolism affects epidermal lipid composition [244]. These locally produced toxins can enter systemic circulation (e.g., uremic toxins from the gut), directly activating inflammatory pathways, exacerbating oxidative stress, and disrupting cellular metabolism. Furthermore, gut dysbiosis impairs BAs metabolism and promotes TMAO production [157], driving systemic metabolic dysfunction, which itself is both a manifestation and accelerator of inflammaging [161]. These four layers are deeply intertwined and mutually amplifying. Barrier breach facilitates antigen translocation; translocated antigens activate immunity and fuel local inflammation; local inflammation further erodes barriers; and the resulting metabolic toxins damage both. Crosstalk between niches via axes ensures that dysregulation in one site propagates systemically, expanding the inflammatory network across the entire host.

### MA: A novel biomarker network quantifying the drivers of inflammaging

Evolving from a descriptive observation to a potential clinical tool, MA serves as a comprehensive, multi‐niche indicator for disease prediction, health assessment, and monitoring intervention responses. As the primary driver, gut‐MA quantifies the cumulative burden of chronic low‐grade inflammation, metabolic stress, and immune load. Deviations in gut‐MA strongly correlate with risks for obesity, type 2 diabetes, cardiovascular disease, and various geriatric syndromes [26]. Oral‐MA exhibits significant predictive value. Periodontal patients display a pronounced “age‐advanced” profile, correlating with activated systemic inflammatory pathways and elevated cardio‐metabolic risks [367]. Furthermore, the increasing ratio of periodontal pathogens with age shows a directional association with cognitive decline in old age [368], positioning oral‐MA as a “visual window” into systemic health risks. Skin‐MA expands the applicability of MA. The systematic succession of the cutaneous microbiome reflects coordinated changes in barrier function, sebaceous activity, immune homeostasis, and esthetic aging. Models integrating facial imaging with metagenomics can precisely predict aging phenotypes and even quantify “rejuvenation” trends post‐intervention, highlighting skin‐MA's potential in cosmetic dermatology [240]. Reflecting local hormonal status and inflammatory environments, reproductive‐MA provides critical insights into reproductive aging.

Crucially, MA responds sensitively and continuously to anti‐aging interventions, serving as an ideal quantitative yardstick. (1) Dietary Interventions: Adherence to a Mediterranean diet can significantly boost SCFAs producers and lower inflammatory pathways within a year, improving frailty scores [369]. Short‐term dietary shifts (animal vs. plant‐based) rapidly alter microbial metabolic pathways [76]. (2) Microbial Therapeutics: The individualized response to probiotics/prebiotics underscores MA's utility. Since host microbiota structure dictates probiotic engraftment [370], relying solely on taxonomic abundance is unstable; MA captures the unified signal of structural and functional shifts. (3) FMT and Drug‐Microbiome Interactions: FMT studies demonstrate that holistic microbial restoration is key to sustained efficacy [371, 372]. Furthermore, drug‐microbiome interactions (e.g., Metformin altering SCFAs metabolism [91]) illustrate how pharmacological interventions may modulate systemic aging trajectories via the MA pathway [26]. While valuable individually, the four niche‐specific MAs are essentially ADPs reflecting the degree to which a microbial ecosystem deviates from a healthy trajectory. An accelerated gut‐MA indicates systemic inflammatory/metabolic burden [373, 374]; an advanced oral‐MA flags oral focal inflammation [375]; skin‐MA reflects barrier dysfunction [376]; reproductive‐MA mirrors endocrine shifts [292, 377].

More importantly, these individual MAs constitute a biomarker network. An individual may exhibit concurrent acceleration in gut‐MA and oral‐MA, signaling potent inflammatory drivers from both the digestive and oral systems. Therefore, the future lies in constructing a “X‐MA Integrated Score.” By leveraging machine learning to integrate aging signals from the gut, oral, skin, and genitourinary tracts, this integrated score promises to be a powerful tool for systemically assessing an individual's “microbiome‐driven inflammaging” load. It will enable earlier, more sensitive identification of high‐risk individuals and guide precision targeting (e.g., prioritizing oral health and gut restoration), while monitoring the holistic improvement of the microbial ecosystem in response to anti‐inflammatory and anti‐aging interventions.

### The clinical application prospects and future directions of MA

**Figure 14:**
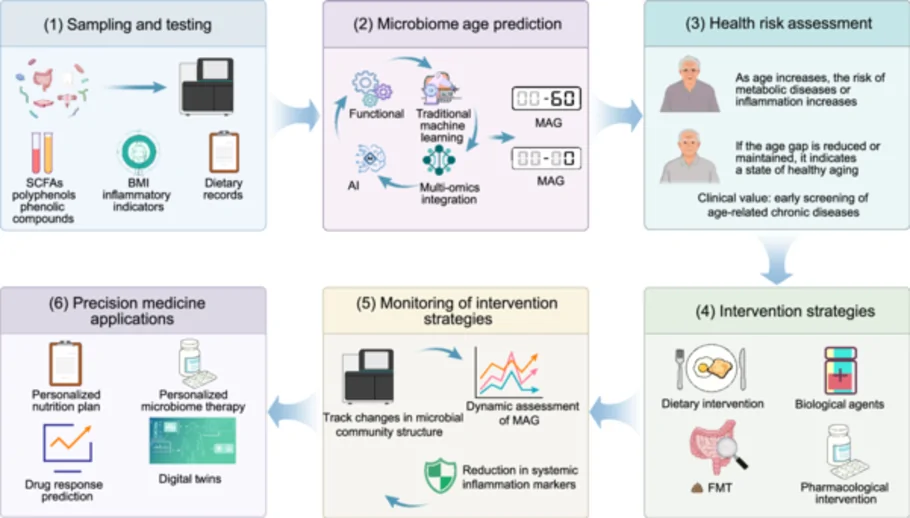
Translational applications and future implementation of microbiome age. The clinical application of microbiota age follows a systematic workflow: detection‐prediction‐assessment‐intervention‐monitoring‐application. First, fecal samples are collected to acquire microbial metabolite profiles (e.g., SCFAs, polyphenols, phenolic compounds), which are integrated with clinical and lifestyle data (including body mass index [BMI], inflammatory indicators, dietary records) to establish a comprehensive baseline dataset. MA is subsequently predicted via diverse computational approaches (traditional machine learning, functional models, multi‐omics integration models, artificial intelligence models), with the MAG derived to indicate deviation from chronological aging. Based on microbiota age and MAG, health risk assessments are performed to identify individuals at elevated risk of metabolic and inflammation‐related disorders, enabling early screening for aging‐related chronic diseases. In the intervention phase, strategies including dietary intervention, biological agents, fecal microbiota transplantation, and pharmacological intervention are applied to modulate microbial structure and function. Post‐intervention dynamic monitoring and reassessment are critical to track changes in microbial composition, MAG reduction, and improvements in metabolic parameters and systemic inflammation. Ultimately, microbiota age holds promise for integration into precision medicine applications, including personalized nutritional regimens, tailored microbiome‐based therapies, drug response prediction, and digital twin model construction, to support aging risk management and individualized health interventions.

The concept of MA, which represents the degree of matching between the structure and function of the intestinal microbiota and the biological age of the host, is closely related to health longevity in multiple dimensions such as immunity, metabolism, neurology, and oncology. It demonstrates clinical potential as an indicator for health assessment, disease prediction, and evaluation of the efficacy of anti‐aging interventions. Based on this, we propose a precise health assessment and intervention strategy based on MA, aiming to provide a new direction for future multi‐omics integrated research (Figure 14).

#### Technical and methodological challenges

For MA to be translatable, it must achieve cross‐platform compatibility, cross‐cohort robustness, and biological interpretability. (1) Multi‐Platform Data Integration. Integrating data across platforms is a critical hurdle [378]. 16S rRNA amplicon sequencing provides ASVs and taxonomic profiles [379], while shotgun metagenomics extends to genes and pathways [378]. Long‐read sequencing and hybrid assembly further resolve strains and structural variants [380], placing “age signals” on unequal feature scales. Directly concatenating these can cause models to learn platform‐specific artifacts rather than biological aging, leading to cross‐platform generalization failure. A more robust approach is to use functional pathways as a relatively unified feature space [381], incorporating covariates like diet and medication. For historical 16S cohorts, functional inference can approximate alignment [382], but such inferences should serve as a supplement, not a replacement, for measured functional data. (2) Batch Effects and Geographic Disparities. Cross‐cohort failure often stems from distribution shift. Batch effects originate from sampling, storage, library prep, sequencing, and bioinformatics pipelines; geographic disparities reflect true differences in diet, lifestyle, and environment. Both can confound age signals. Given the compositional nature and zero‐inflation of microbiome data, simple linear corrections are insufficient or may over‐correct [383]. Best practices involve regression or empirical Bayesian adjustments within the Centered Log‐Ratio or Isometric Log‐Ratio spaces, employing two‐part models to handle zeros and non‐zero distributions separately when necessary [384]. Geographic differences should not be dismissed as noise; instead, they require external validation and metadata stratification to assess transferability, preventing models from degenerating into mere “cohort identifiers” [383, 385]. (3) Rare Taxa and the “Dark Matter” of the Microbiome. The interpretability of MA clocks is limited by the microbiome's “dark matter”—rare taxa, strain variants, mobile elements, and unannotated genes [380, 386, 387, 388]. Short‐read technologies and incomplete reference databases systematically omit these critical nodes, a problem exacerbated in low‐biomass samples (skin, oral) [380, 386]. Solutions require parallel efforts: expanding reference catalogs and annotation resources [386, 387]; adopting spectral imaging and functional‐layer representations for cross‐study comparability [389]; combining long‐read and metagenome‐assembled genomes (MAGs) to improve strain and structural resolution [380]; and utilizing culturomics and single‐cell methods to transform key low‐abundance taxa into manipulable objects, thereby building a falsifiable mechanistic chain linking features → pathways → host phenotypes [390, 391]. (4) Lack of Unified Validation Frameworks for X‐MA. Currently, X‐MA studies across different niches are conducted independently, with inconsistent cohorts, age ranges, data types, feature engineering, and evaluation metrics. This precludes direct comparison of the strength, synchrony, or complementarity of different site‐specific MAs. There is an urgent need to collect synchronized gut, skin, oral, and genitourinary samples within the same cohort, employing unified pipelines to construct X‐MAs and systematically test their correlations and complementarity.

#### Deepening mechanistic research

Research must move beyond “whether MA correlates” to dissecting causality and distinguishing between “driver” and “passenger” roles in aging. (1) Causality between MA and Host Epigenetic Age. Studies are shifting from correlation to causation [392, 393]. Population‐level approaches utilize mQTLs and Mendelian Randomization (MR) [393, 394] alongside longitudinal cohorts to infer directionality [395, 396], though limited by weak instruments and environmental confounders [392, 393, 394]. The gold standard remains functional validation: introducing candidate signals (microbiota, functional modules, metabolites) [397, 398] into germ‐free models or colonization systems to test if they dose‐dependently alter DNA methylation clocks and pathways (DNMT/TET) [399, 400, 401]. (2) Distinguishing “Driver” from “Passenger” Status. Cross‐sectional data cannot determine if MA is a “driver” or a “reflector” of host aging; this requires intervention and counterfactual evidence chains [392]. If microbial changes merely reflect the host milieu, interventions may not alter core aging phenotypes. If causal drivers exist, precision interventions should yield reproducible improvements with reversible windows [397]. Critical distinction lies in whether the intervention merely changes the clock reading or alters the pathological cascade [146], utilizing longitudinal tracking and causal mediation analysis to link metabolites → host receptors → immune/metabolic signaling axis [402, 403]. (3) Functional Validation of Key Age‐Related Microbiota. Model importance features may be proxies for confounders (diet, drugs, hygiene) [119]; we must move from feature importance to experimental validation [392]. Candidate features must be “operationalized” (strains, gene clusters, metabolic reactions, metabolites) [397] and tested via addition/deletion/reintroduction, gene knockout, metabolite supplementation, or receptor blockade in defined gnotobiotic systems to see if they recapitulate age‐related phenotypes or alter clock readings [398]. (4) Causal Partitioning of Host‐Microbe Interactions. Bidirectional influences (immune, metabolic, tissue microenvironment) shape both the microbiota and aging phenotypes; correlation alone (especially stool‐only data) risks misattribution [392]. Experimentally, decouple variables using symbiotic bacteria and tissue‐specific manipulations combined with host multi‐omics to pinpoint pathways [398]. In humans, use multi‐omics linkage and causal mediation to lock onto “microbe‐metabolite‐host phenotype” links, then return to experimental systems for receptor/pathway dependency verification and intervention back‐validation [404, 405, 406].

#### Clinical translation pathways

Clinical translation hinges on three pillars: reference standards, multicenter validation, and standardized protocols. (1) Establishing a Global MA Reference Database. A global database is prerequisite [407]: providing reference distributions for specific age brackets across geographies, diets, and platforms, allowing MAG to be interpreted as deviations from normal ranges [408]. Construction should leverage re‐analyzable public data as a base, unifying feature spaces and minimum metadata standards with versioned iterations [409]. It must integrate multi‐niche data (gut/skin/oral/reproductive) and incorporate key exposure metadata (diet, meds, skincare, oral hygiene, menstrual cycle/contraception/hormonal status) to support clinical interpretation. (2) Multicenter, Cross‐Regional Validation Cohorts. Multicenter validation is the entry barrier [410]; single‐center high performance does not equate to real‐world reproducibility [411]. Validation must upgrade from prediction error to outcome‐oriented metrics [411]: testing reading stability, independent contribution to hard outcomes (mortality, AD, CVD), complementarity with epigenetic/immune ages, and trackability of interventions [411]. Cohorts require prespecified stratification by age, region, and key exposures [412], with standardized protocols and reporting to ensure multi‐scenario utility [411]. (3) Standardized Clinical Testing Pipelines. Standardization dictates inspectability, traceability, and regulatory compliance. Pre‐analytical: unify sampling time windows, preservation media, and transport conditions [413]. Analytical: implement SOPs with positive/negative controls and batch monitoring. Bioinformatics: fix versioned pipelines and QC thresholds [413]. Clinical Reports: embed sample handling, sequencing, and computational pipelines plus key metadata fields to guarantee interpretability and comparability [411, 413].

#### Deep integration with AI and digital twins

AI and digital twins can integrate multi‐dimensional data to build interpretable, predictive, and interventional microbe‐host aging models, evolving MA from static assessment to dynamic health management. (1) Multi‐Modal Aging Prediction Models. Fusing microbial functional signals with host multi‐omics and clinical phenotypes enhances generalizability and outcome prediction. This requires explainable and transferable fusion mechanisms [93], explicitly controlling for batch effects and population structure to prevent the model from learning modality or cohort labels [414]. (2) Individualized Intervention Simulation. Models should predict dynamic trajectories of MA and related phenotypes under intervention, not just output a static gap [415]. Digital twins must be falsifiable: validated against prospective intervention cohorts and counterfactual controls [415], calibrating key parameters using in vitro dynamic models, colonized animals, or organoids [414]. (3) Dynamically Adjusted MA Management Systems. A major limitation of current microbiome age models is their predominant reliance on cross‐sectional datasets, which provide only static snapshots of microbial ecology. Consequently, these models often struggle to distinguish physiological aging trajectories from pathological microbiome deviations driven by frailty, chronic disease, medication exposure, malnutrition, or environmental factors. Emerging longitudinal evidence has begun to address this limitation. Larson et al. [21] conducted three repeated samplings over a 3‐month period across the skin, oral cavity, and gut microbiomes of older adults, demonstrating that microbial alterations correlated more strongly with frailty and infection risk than with chronological age, suggesting the influence of pathological deviations. Haran et al. [416] reported in long‐term nursing home follow‐up studies that microbial communities underwent time‐dependent dysbiosis associated with frailty, malnutrition, medication exposure, and institutional environment. Jeffery et al. [417] further distinguished physiological aging trajectories from environment‐ or care‐related pathological deviations by comparing microbial stability between community‐dwelling and long‐term care elderly individuals at 3‐month intervals. In addition, Zhou et al. and Stennett et al. provided multi‐ecosystem longitudinal evidence supporting both individualized microbial stability and dynamic disturbances associated with metabolic abnormalities [418, 419]. Therefore, longitudinal sampling and time‐aware modeling are essential to disentangle transient perturbations from true biological aging signals. Individual microbiomes are continuously perturbed by diet, medications, and illness, resulting in high‐frequency fluctuations. Static models struggle to distinguish transient noise from long‐term trends, leading to drift and false alarms [93]. Implement time‐aware learning and longitudinal trajectory modeling, utilizing repeated sampling to calibrate individual baselines, coupled with drift detection and versioned model updates to distinguish model degradation from true biological transitions [93]. Further, bind reading changes to actionable interventions, creating an “Assess‐Intervene‐Reassess” closed loop [415]. (4) Online Prediction Platform for Microbiome Biological Age. Online platforms should provide clinical‐grade, one‐stop services. Define input formats and QC thresholds, provide stratified reference intervals and uncertainty estimates, output key driving features and confounding alerts, and enable versioned recalculations [414]. Link with the reference system, multicenter validations, and standardized pipelines to establish an audit trail, privacy governance, and drift monitoring [93].

## CONCLUSION

In summary, MA has evolved from an early descriptive concept into a comprehensive multi‐niche biological metric that reflects systemic aging. It captures both common trajectories of microbial succession (e.g., reduced diversity, core microbiota drift, metabolic reprogramming, and inflammatory network activation) and niche‐specific physiological couplings, including links between the oral microbiome and systemic inflammation, the skin microbiome and barrier homeostasis, the reproductive microbiome and hormonal cycles, as well as remote regulatory axes such as the gut–skin, gut–gonad, and gut–brain pathways. These lines of evidence establish the microbiome not as a passive bystander but as a key regulatory hub shaping immunity, metabolism, barrier function, and neurological health. Consequently, MA holds unique advantages in predicting health risks, monitoring disease progression, and evaluating intervention efficacy, with broad translational potential across precision nutrition, pharmaco‐microbiomics, probiotics/postbiotics, engineered bacterial therapeutics, fecal microbiota transplantation, dermatology, and reproductive health.

Of note, MA exhibits significant site‐specificity: gut‐MA primarily reflects metabolic‐inflammatory status, skin‐MA and oral‐MA index barrier function and environmental exposure, and reproductive‐MA indicates hormonally driven life stages. Thus, the MAG should not be dismissed as a technical error but interpreted within its specific ecological and physiological context. Converging evidence strongly suggests that “inflammaging” is the common terminal pathway driving MA convergence across niches. Dysbiosis at a single niche does not merely cause isolated local lesions; through highly convergent molecular and cellular mechanisms, it amplifies and systematizes disruptive signals, ultimately shaping a systemic inflammaging phenotype. Hence, inflammaging can be viewed as the “effector integrator” and “phenotypic amplifier” through which the microbiome exerts its influence on aging.

Different niche‐specific MAs together form a biomarker network. An individual may exhibit accelerated gut‐MA and oral‐MA simultaneously, indicating compounded inflammatory burden. The future lies in constructing an “X‐MA integrated score”—leveraging machine learning to integrate aging signals from the gut, oral cavity, skin, and genitourinary tract. This integrated score would systematically assess an individual's “microbiome‐driven inflammaging load,” enabling earlier identification of high‐risk individuals, guiding precision interventions (e.g., prioritizing oral health and gut restoration), and monitoring holistic improvements in the microbial ecosystem following anti‐inflammatory and anti‐aging interventions.

Looking ahead, MA is poised to become a systemic health metric at the “holobiont” level, capable of characterizing individual physiological age, immune homeostasis, metabolic resilience, and tissue functional reserve. As high‐resolution technologies, multi‐niche integration, mechanistic dissection, AI modeling, and engineered bacterial therapeutics mature, we stand at the threshold of a new era in which “microbial ecosystem stewardship” shapes host healthspan.

## AUTHOR CONTRIBUTIONS

**Zhexin Ni**: Conceptualization; methodology; writing—original draft. **Jiaojiao Xie**: investigation. **Zhichun Zhang**: Writing—original draft. **Keming Zhang**: Methodology. **Jie Ding**: Writing—original draft. **Mingyang Chang**: Writing—original draft; methodology. **Ying Su**: Visualization. **Yunan Zhang**: Visualization; writing—original draft. **Mingfei Yao**: Visualization; investigation. **Chengyuan Wang**: Writing—original draft; visualization. **Yan Yan**: Writing—original draft; investigation. **Wenjie Fang**: Investigation. **Hainv Gao**: Investigation. **Xuecong Tian**: Methodology. **Yangshuo Li**: Methodology. **Xinlin Zhu**: Investigation. **Wen Zhang**: Methodology. **Wanqing Liao**: Investigation. **Gaoyuan Xu**: Methodology. **Guojing Chen**: Investigation. **Xiaoyi Lv**: Project administration; writing—review and editing. **Chaoqin Yu**: Project administration; writing—review and editing. **Weihua Pan**: Project administration; writing—review and editing. **Vadim Molodtsov**: Project administration; writing—review and editing. **Lanjuan Li**: Project administration; writing—review and editing. **Yue Gao**: Project administration; funding acquisition; methodology. **Wei Zhou**: Writing—review and editing; writing—original draft; project administration; conceptualization; methodology; funding acquisition.

## CONFLICT OF INTEREST STATEMENT

The authors declare no conflicts of interest.

## ETHICS STATEMENT

No animals or humans were involved in this study.

## Data Availability

No new data and scripts were used for this review. Supplementary materials (graphical abstract, slides, videos, Chinese translated version, and updated materials) may be found in the online DOI or *iMeta* Science http://www.imeta.science/. Data sharing is not applicable to this article as no datasets were generated or analyzed during the current study.

## References

[1] López‐Otín, Carlos , Maria A. Blasco , Linda Partridge , Manuel Serrano , and Guido Kroemer . 2023. “Hallmarks of Aging: An Expanding Universe.” Cell 186: 243–278. 10.1016/j.cell.2022.11.001 36599349

[2] Zhu, Ziwei , Jingjing Lyu , Xingjie Hao , Huan Guo , Xiaomin Zhang , Meian He , Xiang Cheng , Shanshan Cheng , and Chaolong Wang . 2024. “Estimation of Physiological Aging Based on Routine Clinical Biomarkers: A Prospective Cohort Study in Elderly Chinese and the UK Biobank.” BMC Medicine 22: 552. 10.1186/s12916-024-03769-2 39578829 PMC11583456

[3] Apsley, Abner T. , Laura Etzel , Qiaofeng Ye , and Idan Shalev . 2025. “From Population Science to the Clinic? Limits of Epigenetic Clocks as Personal Biomarkers.” Epigenomics 17: 1447–1461. 10.1080/17501911.2025.2603880 41403206 PMC12714307

[4] Alpert, Ayelet , Yishai Pickman , Michael Leipold , Yael Rosenberg‐Hasson , Xuhuai Ji , Renaud Gaujoux , Hadas Rabani , et al. 2019. “A Clinically Meaningful Metric of Immune Age Derived From High‐Dimensional Longitudinal Monitoring.” Nature Medicine 25: 487–495. 10.1038/s41591-019-0381-y PMC6686855 30842675

[5] Donati Zeppa, Sabrina , Deborah Agostini , Fabio Ferrini , Marco Gervasi , Elena Barbieri , Alessia Bartolacci , Giovanni Piccoli , Roberta Saltarelli, Piero Sestili, and Vilberto Stocchi. 2022. “Interventions on Gut Microbiota for Healthy Aging.” Cells 12: 34. 10.3390/cells12010034 36611827 PMC9818603

[6] Min, Mildred , Caitlin Egli , and Raja K. Sivamani . 2024. “The Gut and Skin Microbiome and Its Association With Aging Clocks.” International Journal of Molecular Sciences 25: 7471. 10.3390/ijms25137471 39000578 PMC11242811

[7] Myers, Tyler , Se Jin Song , Yang Chen , Britta De Pessemier , Lora Khatib , Daniel McDonald , Shi Huang , et al. 2025. “Chronological Age Estimation From Human Microbiomes With Transformer‐Based Robust Principal Component Analysis.” Communications Biology 8: 1159. 10.1038/s42003-025-08590-y 40770074 PMC12328700

[8] Nie, Chao , Yan Li , Rui Li , Yizhen Yan , Detao Zhang , Tao Li , Zhiming Li , et al. 2022. “Distinct Biological Ages of Organs and Systems Identified From a Multi‐Omics Study.” Cell Reports 38: 110459. 10.1016/j.celrep.2022.110459 35263580

[9] Galkin, Fedor , Polina Mamoshina , Alex Aliper , Eugene Lane , Vladimir Moskalev , Vadim N. Gladyshev , and Alex Zhavoronkov . 2020. “Human Gut Microbiome Aging Clock Based on Taxonomic Profiling and Deep Learning.” iScience 23: 101199. 10.1016/j.isci.2020.101199 32534441 PMC7298543

[10] Martino, Cameron , Amanda Hazel Dilmore , Zachary M. Burcham , Jessica L. Metcalf , Dilip Jeste , and Rob Knight . 2022. “Microbiota Succession Throughout Life From the Cradle to the Grave.” Nature Reviews Microbiology 20: 707–720. 10.1038/s41579-022-00768-z 35906422 PMC12875531

[11] Badal, Varsha D. , Eleonora D. Vaccariello , Emily R. Murray , Kasey E. Yu , Rob Knight , Dilip V. Jeste , and Tanya T. Nguyen . 2020. “The Gut Microbiome, Aging, and Longevity: A Systematic Review.” Nutrients 12: 3759. 10.3390/nu12123759 33297486 PMC7762384

[12] Biagi, Elena , Claudio Franceschi , Simone Rampelli , Marco Severgnini , Rita Ostan , Silvia Turroni , Clarissa Consolandi , et al. 2016. “Gut Microbiota and Extreme Longevity.” Current Biology 26: 1480–1485. 10.1016/j.cub.2016.04.016 27185560

[13] Belkaid, Yasmine , and Timothy W. Hand . 2014. “Role of the Microbiota in Immunity and Inflammation.” Cell 157: 121–141. 10.1016/j.cell.2014.03.011 24679531 PMC4056765

[14] Ratiner, Karina , Suhaib K. Abdeen , Kim Goldenberg , and Eran Elinav . 2022. “Utilization of Host and Microbiome Features in Determination of Biological Aging.” Microorganisms 10: 668. 10.3390/microorganisms10030668 35336242 PMC8950177

[15] Popkes, Miriam , and Dario Riccardo Valenzano . 2020. “Microbiota‐Host Interactions Shape Ageing Dynamics.” Philosophical Transactions of the Royal Society B: Biological Sciences 375: 20190596. 10.1098/rstb.2019.0596 PMC7435156 32772667

[16] Honda, Kenya , and Dan R. Littman . 2016. “The Microbiota in Adaptive Immune Homeostasis and Disease.” Nature 535: 75–84. 10.1038/nature18848 27383982

[17] Simpson, Daniel J. , and Tamir Chandra . 2021. “Epigenetic Age Prediction.” Aging Cell 20: e13452. 10.1111/acel.13452 34415665 PMC8441394

[18] Li, Wenchao , Zhenhua Zhang , Saumya Kumar , Javier Botey‐Bataller , Martijn Zoodsma , Ali Ehsani , Qiuyao Zhan , et al. 2025. “Single‐Cell Immune Aging Clocks Reveal Inter‐Individual Heterogeneity During Infection and Vaccination.” Nature Aging 5: 607–621. 10.1038/s43587-025-00819-z 40044970 PMC12003178

[19] Best, Lena , Thomas Dost , Daniela Esser , Stefano Flor , Andy Mercado Gamarra , Madlen Haase , A Samer Kadibalban , et al. 2025. “Metabolic Modelling Reveals the Aging‐Associated Decline of Host‐microbiome Metabolic Interactions in Mice.” Nature Microbiology 10: 973–991. 10.1038/s41564-025-01959-z PMC11964932 40140706

[20] Huang, Shi , Niina Haiminen , Anna‐Paola Carrieri , Rebecca Hu , Lingjing Jiang , Laxmi Parida , Baylee Russell , et al. 2020. “Human Skin, Oral, and Gut Microbiomes Predict Chronological Age.” mSystems 5: e00630–19. 10.1128/mSystems.00630-19 32047061 PMC7018528

[21] Larson, Peter J. , Wei Zhou , Alba Santiago , Sarah Driscoll , Elizabeth Fleming , Anita Y. Voigt , Ock K. Chun , et al. 2022. “Associations of the Skin, Oral and Gut Microbiome With Aging, Frailty and Infection Risk Reservoirs in Older Adults.” Nature Aging 2: 941–955. 10.1038/s43587-022-00287-9 36398033 PMC9667708

[22] DeClercq, Vanessa , Robyn J. Wright , Jacob T. Nearing , and Morgan G. I. Langille . 2024. “Oral Microbial Signatures Associated With Age and Frailty in Canadian Adults.” Scientific Reports 14: 9685. 10.1038/s41598-024-60409-8 38678061 PMC11055859

[23] Gopu, Vishakh , Francine R. Camacho , Ryan Toma , Pedro J. Torres , Ying Cai , Subha Krishnan , Sathyapriya Rajagopal , et al. 2024. “An Accurate Aging Clock Developed From Large‐scale Gut Microbiome and Human Gene Expression Data.” iScience 27: 108538. 10.1016/j.isci.2023.108538 38230258 PMC10790003

[24] Łaniewski, Paweł , and Melissa M. Herbst‐Kralovetz . 2022. “Connecting Microbiome and Menopause for Healthy Ageing.” Nature Microbiology 7: 354–358. 10.1038/s41564-022-01071-6 PMC9977513 35246661

[25] Wehnes, Tim , Weilan Wang , Jian Hua Tay , Lihuan Guan , Lei Feng , Chun Man Roger Ho , Tze Pin Ng , et al. 2025. “Oral Short‐chain Fatty Acid‐producing Bacteria May Be Associated With Biological Age and Cognition Among the Oldest Old.” Communications Medicine 6: 37. 10.1038/s43856-025-01288-6 41422165 PMC12819410

[26] Wilmanski, Tomasz , Christian Diener , Noa Rappaport , Sushmita Patwardhan , Jack Wiedrick , Jodi Lapidus , John C. Earls , et al. 2021. “Gut Microbiome Pattern Reflects Healthy Ageing and Predicts Survival in Humans.” Nature Metabolism 3: 274–286. 10.1038/s42255-021-00348-0 PMC8169080 33619379

[27] Xu, Tiansong , Yuting Niu , Chenyu Deng , Yoo Cheung , Yuman Li , Zhewen Hu , Shiyu Sun , et al. 2025. “Saliva MicroAge: A SAalivary Microbiome Based Machine Learning Model for Noninvasive Aging Assessment and Health State Prediction.” iMetaOmics 2: e70040. 10.1002/imo2.70040 41676445 PMC12806003

[28] Kim, Sukyung , Hoonhee Seo , Md Abdur Rahim , Saebim Lee , Yun‐Sook Kim , and Ho‐Yeon Song . 2021. “Changes in the Microbiome of Vaginal Fluid After Menopause in Korean Women.” Journal of Microbiology and Biotechnology 31: 1490–1500. 10.4014/jmb.2106.06022 34489372 PMC9705842

[29] Yoshikata, Remi , Michiko Yamaguchi , Yuri Mase , Ayano Tatsuzuki , Khin Zay Yar Myint , and Hiroaki Ohta . 2022. “Age‐Related Changes, Influencing Factors, and Crosstalk Between Vaginal and Gut Microbiota: A Cross‐sectional Comparative Study of Pre‐ and Postmenopausal Women.” Journal of Women's Health 31: 1763–1772. 10.1089/jwh.2022.0114 36374244

[30] Zhou, Yi‐Hui , and Paul Gallins . 2019. “A Review and Tutorial of Machine Learning Methods for Microbiome Host Trait Prediction.” Frontiers in Genetics 10: 579. 10.3389/fgene.2019.00579 31293616 PMC6603228

[31] Subramanian, Sathish , Sayeeda Huq , Tanya Yatsunenko , Rashidul Haque , Mustafa Mahfuz , Mohammed A. Alam , Amber Benezra , et al. 2014. “Persistent Gut Microbiota Immaturity in Malnourished Bangladeshi Children.” Nature 510: 417–421. 10.1038/nature13421 24896187 PMC4189846

[32] Li, Shao‐Hua , Ming Song , Peng Wang , Tian‐Shun Kou , Xuan‐Xian Peng , Hua Ye , and Hui Li . 2025. “XGBoost‐ and Mass Spectrometry‐based Feature Selection for Identifying Metabolic Biomarkers Associated With HBV‐Related Liver Disease Progression and Hepatocellular Carcinoma Treatment.” Journal of Proteome Research 24: 5803–5817. 10.1021/acs.jproteome.5c00540 41088963

[33] Liu, Yinuo , and Ziwei Chen . 2025. “LightGBM‐Based Human Action Recognition Using Sensors.” Sensors 25: 3704. 10.3390/s25123704 40573590 PMC12196585

[34] Li, Peishun , Hao Luo , Boyang Ji , and Jens Nielsen . 2022. “Machine Learning for Data Integration in Human Gut Microbiome.” Microbial Cell Factories 21: 241. 10.1186/s12934-022-01973-4 36419034 PMC9685977

[35] Marcos‐Zambrano, Laura Judith , Kanita Karaduzovic‐Hadziabdic , Tatjana Loncar Turukalo , Piotr Przymus , Vladimir Trajkovik , Oliver Aasmets , Magali Berland , et al. 2021. “Applications of Machine Learning in Human Microbiome Studies: A Review on Feature Selection, Biomarker Identification, Disease Prediction, and Treatment.” Frontiers in Microbiology 12: 634511. 10.3389/fmicb.2021.634511 33737920 PMC7962872

[36] Odamaki, Toshitaka , Kumiko Kato , Hirosuke Sugahara , Nanami Hashikura , Sachiko Takahashi , Jin‐Zhong Xiao , Fumiaki Abe , and Ro Osawa . 2016. “Age‐Related Changes in Gut Microbiota Composition From Newborn to Centenarian: A Cross‐Sectional Study.” BMC Microbiology 16: 90. 10.1186/s12866-016-0708-5 27220822 PMC4879732

[37] Plovier, Hubert , Amandine Everard , Céline Druart , Clara Depommier , Matthias Van Hul , Lucie Geurts , Julien Chilloux , et al. 2017. “A Purified Membrane Protein From *Akkermansia muciniphila* or the Pasteurized Bacterium Improves Metabolism in Obese and Diabetic Mice.” Nature Medicine 23: 107–113. 10.1038/nm.4236 27892954

[38] Ghosh, Tarini Shankar , Fergus Shanahan , and Paul W. O'Toole . 2022. “The Gut Microbiome as a Modulator of Healthy Ageing.” Nature Reviews Gastroenterology & Hepatology 19: 565–584. 10.1038/s41575-022-00605-x 35468952 PMC9035980

[39] He, Yan , Wei Wu , Hui‐Min Zheng , Pan Li , Daniel McDonald , Hua‐Fang Sheng , Mu‐Xuan Chen , et al. 2018. “Regional Variation Limits Applications of Healthy Gut Microbiome Reference Ranges and Disease Models.” Nature Medicine 24: 1532–1535. 10.1038/s41591-018-0164-x 30150716

[40] De Filippis, Francesca , Nicoletta Pellegrini , Lucia Vannini , Ian B. Jeffery , Antonietta La Storia , Luca Laghi , Diana I. Serrazanetti , et al. 2016. “High‐Level Adherence to a Mediterranean Diet Beneficially Impacts the Gut Microbiota and Associated Metabolome.” Gut 65: 1812–1821. 10.1136/gutjnl-2015-309957 26416813

[41] Visconti, Alessia , Caroline I. Le Roy , Fabio Rosa , Niccolò Rossi , Tiphaine C. Martin , Robert P. Mohney , Weizhong Li , et al. 2019. “Interplay Between the Human Gut Microbiome and Host Metabolism.” Nature Communications 10: 4505. 10.1038/s41467-019-12476-z PMC6776654 31582752

[42] Tierney, Braden T. , Zhen Yang , Jacob M. Luber , Marc Beaudin , Marsha C. Wibowo , Christina Baek , E. Mehlenbacher , et al. 2019. “The Landscape of Genetic Content in the Gut and Oral Human Microbiome.” Cell Host & Microbe 26: 283–295. 10.1016/j.chom.2019.07.008 31415755 PMC6716383

[43] Louca, Stilianos , Laura Wegener Parfrey , and Michael Doebeli . 2016. “Decoupling Function and Taxonomy in the Global Ocean Microbiome.” Science 353: 1272–1277. 10.1126/science.aaf4507 27634532

[44] Kanehisa, M. , and Susumu Goto. 2000. “KEGG: Kyoto Encyclopedia of Genes and Genomes.” Nucleic Acids Research 28: 27–30. 10.1093/nar/28.1.27 10592173 PMC102409

[45] Caspi, Ron , Richard Billington , Luciana Ferrer , Hartmut Foerster , Carol A. Fulcher , Ingrid M. Keseler , Anamika Kothari , et al. 2016. “The MetaCyc Database of Metabolic Pathways and Enzymes and the BioCyc Collection of Pathway/genome Databases.” Nucleic Acids Research 44: D471–D480. 10.1093/nar/gkv1164 26527732 PMC4702838

[46] Rampelli, Simone , Marco Candela , Silvia Turroni , Elena Biagi , Sebastiano Collino , Claudio Franceschi , Paul W. O'Toole , and Patrizia Brigidi . 2013. “Functional Metagenomic Profiling of Intestinal Microbiome in Extreme Ageing.” Aging 5(12): 902–912. 10.18632/aging.100623 24334635 PMC3883706

[47] Wang, Jun , and Huijue Jia . 2016. “Metagenome‐Wide Association Studies: Fine‐mining the Microbiome.” Nature Reviews Microbiology 14: 508–522. 10.1038/nrmicro.2016.83 27396567

[48] Sato, Yuko , Koji Atarashi , Damian R. Plichta , Yasumichi Arai , Satoshi Sasajima , Sean M. Kearney , Wataru Suda , et al. 2021. “Novel Bile Acid Biosynthetic Pathways Are Enriched in the Microbiome of Centenarians.” Nature 599: 458–464. 10.1038/s41586-021-03832-5 34325466

[49] Vital, Marius , Adina Chuang Howe , and James M. Tiedje . 2014. “Revealing the Bacterial Butyrate Synthesis Pathways by Analyzing (meta)genomic Data.” mBio 5: e00889. 10.1128/mBio.00889-14 24757212 PMC3994512

[50] McNabney, Sean , and Tara Henagan . 2017. “Short Chain Fatty Acids In the Colon and Peripheral Tissues: A Focus on Butyrate, Colon Cancer, Obesity and Insulin Resistance.” Nutrients 9: 1348. 10.3390/nu9121348 29231905 PMC5748798

[51] Li, Min , Baohong Wang , Menghui Zhang , Mattias Rantalainen , Shengyue Wang , Haokui Zhou , Yan Zhang , et al. 2008. “Symbiotic Gut Microbes Modulate Human Metabolic Phenotypes.” Proceedings of the National Academy of Sciences 105: 2117–2122. 10.1073/pnas.0712038105 PMC2538887 18252821

[52] Dalile, Boushra , Lukas Van Oudenhove , Bram Vervliet , and Kristin Verbeke . 2019. “The Role of Short‐Chain Fatty Acids in Microbiota‐Gut‐Brain Communication.” Nature Reviews Gastroenterology & Hepatology 16: 461–478. 10.1038/s41575-019-0157-3 31123355

[53] Nakamura, Atsuo , Takushi Ooga , and Mitsuharu Matsumoto . 2019. “Intestinal Luminal Putrescine is Produced by Collective Biosynthetic Pathways of the Commensal Microbiome.” Gut Microbes 10: 159–171. 10.1080/19490976.2018.1494466 30183487 PMC6546329

[54] Madeo, Frank , Tobias Eisenberg , Federico Pietrocola , and Guido Kroemer . 2018. “Spermidine in Health and Disease.” Science 359: eaan2788. 10.1126/science.aan2788 29371440

[55] Wang, Zeneng , Elizabeth Klipfell , Brian J. Bennett , Robert Koeth , Bruce S. Levison , Brandon Dugar , Ariel E. Feldstein , et al. 2011. “Gut Flora Metabolism of Phosphatidylcholine Promotes Cardiovascular Disease.” Nature 472: 57–63. 10.1038/nature09922 21475195 PMC3086762

[56] Tang, W H Wilson , Zeneng Wang , Bruce S. Levison , Robert A. Koeth , Earl B. Britt , Xiaoming Fu , Yuping Wu , and Stanley L. Hazen . 2013. “Intestinal Microbial Metabolism of Phosphatidylcholine and Cardiovascular Risk.” New England Journal of Medicine 368: 1575–1584. 10.1056/NEJMoa1109400 23614584 PMC3701945

[57] Zhu, Weifei , Jill C. Gregory , Elin Org , Jennifer A. Buffa , Nilaksh Gupta , Zeneng Wang , Lin Li , et al. 2016. “Gut Microbial Metabolite TMAO Enhances Platelet Hyperreactivity and Thrombosis Risk.” Cell 165: 111–124. 10.1016/j.cell.2016.02.011 26972052 PMC4862743

[58] Qiu, Xiaorou , Chao Mu , Jie Hu , Jiaxin Yu , Wenbo Tang , Yueli Liu , Yongmei Huang , et al. 2025. “Serum Metabolic and Microbial Profiling Yields Insights Into Promoting Effect of Tryptophan‐Related Metabolites for Health Longevity in Centenarians.” iMeta 4: e70025. 10.1002/imt2.70025 40469513 PMC12130558

[59] Galkin, Fedor , Polina Mamoshina , Alex Aliper , João Pedro de Magalhães , Vadim N. Gladyshev , and Alex Zhavoronkov . 2020. “Biohorology and Biomarkers of Aging: Current State‐of‐the‐Art, Challenges and Opportunities.” Ageing Research Reviews 60: 101050. 10.1016/j.arr.2020.101050 32272169

[60] Carrieri, Anna Paola , Niina Haiminen , Sean Maudsley‐Barton , Laura‐Jayne Gardiner , Barry Murphy , Andrew E. Mayes , Sarah Paterson , et al. 2021. “Explainable AI Reveals Changes in Skin Microbiome Composition Linked to Phenotypic Differences.” Scientific Reports 11: 4565. 10.1038/s41598-021-83922-6 33633172 PMC7907326

[61] Jackson, Matthew A. , Serena Verdi , Maria‐Emanuela Maxan , Cheol Min Shin , Jonas Zierer , Ruth C. E. Bowyer , Tiphaine Martin , et al. 2018. “Gut Microbiota Associations With Common Diseases and Prescription Medications in a Population‐Based Cohort.” Nature Communications 9: 2655. 10.1038/s41467-018-05184-7 PMC6037668 29985401

[62] Rosenberg, Eugene , and Ilana Zilber‐Rosenberg . 2019. “The Hologenome Concept of Evolution: Medical Implications.” Rambam Maimonides Medical Journal 10: e0005. 10.5041/RMMJ.10359 30720424 PMC6363370

[63] Nicholson, J. K. , J. C. Lindon , and E. Holmes . 1999. “Metabonomics’: Understanding the Metabolic Responses of Living Systems to Pathophysiological Stimuli via Multivariate Statistical Analysis of Biological NMR Spectroscopic Data.” Xenobiotica 29: 1181–1189. 10.1080/004982599238047 10598751

[64] Davis, Mark M. , Cristina M. Tato , and David Furman . 2017. “Systems Immunology: Just Getting Started.” Nature Immunology 18: 725–732. 10.1038/ni.3768 28632713 PMC5790187

[65] Zhou, Wenyu , M Reza Sailani , Kévin Contrepois , Yanjiao Zhou , Sara Ahadi , Shana R. Leopold , Martin J. Zhang , et al. 2019. “Longitudinal Multi‐omics of Host‐microbe Dynamics in Prediabetes.” Nature 569: 663–671. 10.1038/s41586-019-1236-x 31142858 PMC6666404

[66] Ahadi, Sara , Wenyu Zhou , Sophia Miryam Schüssler‐Fiorenza Rose , M Reza Sailani , Kévin Contrepois , Monika Avina , Melanie Ashland , Anne Brunet , and Michael Snyder . 2020. “Personal Aging Markers and Ageotypes Revealed by Deep Longitudinal Profiling.” Nature Medicine 26: 83–90. 10.1038/s41591-019-0719-5 PMC7301912 31932806

[67] Sayed, Nazish , Yingxiang Huang , Khiem Nguyen , Zuzana Krejciova‐Rajaniemi , Anissa P. Grawe , Tianxiang Gao , Robert Tibshirani , et al. 2021. “An Inflammatory Aging Clock (iAge) Based on Deep Learning Tracks Multimorbidity, Immunosenescence, Frailty and Cardiovascular Aging.” Nature Aging 1: 598–615. 10.1038/s43587-021-00082-y 34888528 PMC8654267

[68] Laird, Nan M. , and James H. Ware . 1982. “Random‐Effects Models for Longitudinal Data.” Biometrics 38: 963–974. 10.2307/2529876 7168798

[69] Bokulich, Nicholas A. , Matthew R. Dillon , Yilong Zhang , Jai Ram Rideout , Evan Bolyen , Huilin Li , Paul S. Albert , and J. Gregory Caporaso . 2018. “q2‐Longitudinal: Longitudinal and Paired‐Sample Analyses of Microbiome Data.” mSystems 3: e00219–18. 10.1128/mSystems.00219-18 PMC6247016 30505944

[70] Lloyd‐Price, Jason , Cesar Arze , Ashwin N. Ananthakrishnan , Melanie Schirmer , Julian Avila‐Pacheco , Tiffany W. Poon , Elizabeth Andrews , et al. 2019. “Multi‐Omics of the Gut Microbial Ecosystem in Inflammatory Bowel Diseases.” Nature 569: 655–662. 10.1038/s41586-019-1237-9 31142855 PMC6650278

[71] Chen, Lianmin , Daoming Wang , Sanzhima Garmaeva , Alexander Kurilshikov , Arnau Vich Vila , Ranko Gacesa , Trishla Sinha , et al. 2021. “The Long‐term Genetic Stability and Individual Specificity of the Human Gut Microbiome.” Cell 184: 2302–2315.e12. 10.1016/j.cell.2021.03.024 33838112

[72] Trapnell, Cole , Davide Cacchiarelli , Jonna Grimsby , Prapti Pokharel , Shuqiang Li , Michael Morse , Niall J. Lennon , et al. 2014. “The Dynamics and Regulators of Cell Fate Decisions Are Revealed by Pseudotemporal Ordering of Single Cells.” Nature Biotechnology 32: 381–386. 10.1038/nbt.2859 PMC4122333 24658644

[73] Dai, Yifan , Yunzhi Qian , Yixiang Qu , Wyliena Guan , Jialiu Xie , Duan Wang , Catherine Butler , et al. 2025. “Decoding Longitudinal Microbiome Trajectories: An Interpretable Machine Learning Approach for Biomarker Discovery and Prediction.” Briefings in Bioinformatics 26: bbaf374. 10.1093/bib/bbaf374 40794955 PMC12342957

[74] Cao, Junyue , Malte Spielmann , Xiaojie Qiu , Xingfan Huang , Daniel M. Ibrahim , Andrew J. Hill , Fan Zhang , et al. 2019. “The Single‐cell Transcriptional Landscape of Mammalian OrganogenesiS.” Nature 566: 496–502. 10.1038/s41586-019-0969-x 30787437 PMC6434952

[75] Faith, Jeremiah J. , Janaki L. Guruge , Mark Charbonneau , Sathish Subramanian , Henning Seedorf , Andrew L. Goodman , Jose C. Clemente , et al. 2013. “The Long‐term Stability of the Human Gut Microbiota.” Science 341: 1237439. 10.1126/science.1237439 23828941 PMC3791589

[76] Scheffer, Marten , Jordi Bascompte , William A. Brock , Victor Brovkin , Stephen R. Carpenter , Vasilis Dakos , Hermann Held , et al. 2009. “Early‐Warning Signals for Critical Transitions.” Nature 461: 53–59. 10.1038/nature08227 19727193

[77] Yatsunenko, Tanya , Federico E. Rey , Mark J. Manary , Indi Trehan , Maria Gloria Dominguez‐Bello , Monica Contreras , Magda Magris , et al. 2012. “Human Gut Microbiome Viewed Across Age and Geography.” Nature 486: 222–227. 10.1038/nature11053 22699611 PMC3376388

[78] West, James , Ginestra Bianconi , Simone Severini , and Andrew E. Teschendorff . 2012. “Differential Network Entropy Reveals Cancer System Hallmarks.” Scientific Reports 2: 802. 10.1038/srep00802. 23150773 PMC3496163

[79] Faust, Karoline , and Jeroen Raes . 2012. “Microbial Interactions: From Networks to Models.” Nature Reviews Microbiology 10: 538–550. 10.1038/nrmicro2832 22796884

[80] Mallick, Himel , Eric A. Franzosa , Lauren J. McLver , Soumya Banerjee , Alexandra Sirota‐Madi , Aleksandar D. Kostic , Clary B. Clish , et al. 2019. “Predictive Metabolomic Profiling of Microbial Communities Using Amplicon or Metagenomic Sequences.” Nature Communications 10: 3136. 10.1038/s41467-019-10927-1 PMC6637180 31316056

[81] Segata, Nicola , Jacques Izard , Levi Waldron , Dirk Gevers , Larisa Miropolsky , and Curtis Huttenhower . 2011. “Metagenomic Biomarker Discovery and Explanation.” Genome Biology 12: R60. 10.1186/gb-2011-12-6-r60 21702898 PMC3218848

[82] Bolyen, Evan , Jai Ram Rideout , Matthew R. Dillon , Nicholas A. Bokulich , Christian C. Abnet , Gabriel A. Al‐Ghalith , Harriet Alexander , et al. 2019. “Reproducible, Interactive, Scalable and Extensible Microbiome Data Science Using QIIME 2.” Nature Biotechnology 37: 852–857. 10.1038/s41587-019-0209-9 PMC7015180 31341288

[83] Sun, Chuqing , Guoru Hu , Liwen Yi , Wei Ge , Qingyu Yang , Xiangliang Yang , Yifan He , Zhi Liu , and Wei‐Hua Chen . 2024. “Integrated Analysis of Facial Microbiome and Skin Physio‐Optical Properties Unveils Cutotype‐Dependent Aging Effects.” Microbiome 12: 163. 10.1186/s40168-024-01891-0 39232827 PMC11376020

[84] Grice, Elizabeth A. , Heidi H. Kong , Sean Conlan , Clayton B. Deming , Joie Davis , Alice C. Young , NISC Comparative Sequencing Program , Alice C. Young , Gerard G. Bouffard , et al. 2009. “Topographical and Temporal Diversity of the Human Skin Microbiome.” Science 324: 1190–1192. 10.1126/science.1171700 19478181 PMC2805064

[85] Kim, Gihyeon , Misun Kim , Minji Kim , Changho Park , Youngmin Yoon , Doo‐Hyeon Lim , Hyeonju Yeo , et al. 2021. “Spermidine‐Induced Recovery of Human Dermal Structure and Barrier Function by Skin Microbiome.” Communications Biology 4: 231. 10.1038/s42003-020-01619-4 33608630 PMC7895926

[86] Huang, Shi , Tao He , Feng Yue , Xiujun Xu , Lijiang Wang , Pengfei Zhu , Fei Teng , et al. 2021. “Longitudinal Multi‐Omics and Microbiome Meta‐analysis Identify an Asymptomatic Gingival State that Links Gingivitis, Periodontitis, and Aging.” mBio 12: e03281‐20. 10.1128/mBio.03281-20 33688007 PMC8092283

[87] Hernández Medina, Ricardo , Svetlana Kutuzova , Knud Nor Nielsen , Joachim Johansen , Lars Hestbjerg Hansen , Mads Nielsen , and Simon Rasmussen . 2022. “Machine Learning and Deep Learning Applications in Microbiome Research.” ISME Communications 2: 98. 10.1038/s43705-022-00182-9 37938690 PMC9723725

[88] Oh, Min , and Liqing Zhang . 2020. “DeepMicro: Deep Representation Learning for Disease Prediction Based on Microbiome Data.” Scientific Reports 10: 6026. 10.1038/s41598-020-63159-5 32265477 PMC7138789

[89] Liang, Qiaoxing , Paul W. Bible , Yu Liu , Bin Zou , and Lai Wei . 2020. “DeepMicrobes: Taxonomic Classification for Metagenomics With Deep Learning.” NAR Genomics and Bioinformatics 2: lqaa009. 10.1093/nargab/lqaa009 33575556 PMC7671387

[90] Reiman, Derek , Ahmed A. Metwally , Jun Sun , and Yang Dai . 2020. “PopPhy‐CNN: a Phylogenetic Tree Embedded Architecture For Convolutional Neural Networks to Predict Host Phenotype From Metagenomic Data.” IEEE Journal of Biomedical and Health Informatics 24: 2993–3001. 10.1109/JBHI.2020.2993761 32396115

[91] Wimmer, Moritz I. , Hendrik Bartolomaeus , Harithaa Anandakumar , Chia‐Yu Chen , Valentin Vecera , Sarah Kedziora , Sakshi Kamboj , et al. 2024. “Metformin Modulates Microbiota and Improves Blood Pressure and Cardiac Remodeling in a Rat Model of Hypertension.” Acta Physiologica 240: e14226. 10.1111/apha.14226 39253815

[92] Li, Yikuan , Ramsey M. Wehbe , Faraz S. Ahmad , Hanyin Wang , and Yuan Luo . 2023. “A Comparative Study of Pretrained Language Models for Long Clinical Text.” Journal of the American Medical Informatics Association 30: 340–347. 10.1093/jamia/ocac225 36451266 PMC9846675

[93] Przymus, Piotr , Krzysztof Rykaczewski , Adrián Martín‐Segura , Jaak Truu , Enrique Carrillo De Santa Pau , Mikhail Kolev , Irina Naskinova , et al. 2024. “Deep Learning in Microbiome Analysis: A Comprehensive Review of Neural Network Models.” Frontiers in Microbiology 15: 1516667. 10.3389/fmicb.2024.1516667 39911715 PMC11794229

[94] Chetty, Ashwin , and Ran Blekhman . 2024. “Multi‐Omic Approaches for Host‐microbiome Data Integration.” Gut Microbes 16: 2297860. 10.1080/19490976.2023.2297860 38166610 PMC10766395

[95] Wang, Tongxin , Wei Shao , Zhi Huang , Haixu Tang , Jie Zhang , Zhengming Ding , and Kun Huang . 2021. “Mogonet Integrates Multi‐omics Data Using Graph Convolutional Networks Allowing Patient Classification and Biomarker Identification.” Nature Communications 12: 3445. 10.1038/s41467-021-23774-w PMC8187432 34103512

[96] Aminian‐Dehkordi, Javad , Mohammad Parsa , Andrew Dickson , and Mohammad R. K. Mofrad . 2025. “SIMBA‐GNN: Mechanistic Graph Learning for Microbiome Prediction.” npj Systems Biology and Applications 12: 8. 10.1038/s41540-025-00631-w 41387745 PMC12796443

[97] Goetz, Skye L. , Amy K. Glen , and Gwênlyn Glusman . 2025. “MicrobiomeKG: Bridging Microbiome Research and Host Health Through Knowledge Graphs.” Frontiers in Systems Biology 5: 1544432. 10.3389/fsysb.2025.1544432 40951793 PMC12425944

[98] Chandak, Payal , Kexin Huang , and Marinka Zitnik . 2023. “Building a Knowledge Graph to Enable Precision Medicine.” Scientific Data 10: 67. 10.1038/s41597-023-01960-3 36732524 PMC9893183

[99] Sizemore, Nicholas , Kaitlyn Oliphant , Ruolin Zheng , Camilia R. Martin , Erika C. Claud , and Ishanu Chattopadhyay . 2024. “A Digital Twin of the Infant Microbiome to Predict Neurodevelopmental Deficits.” Science Advances 10: eadj0400. 10.1126/sciadv.adj0400 38598636 PMC11006218

[100] Liu, Ting , K Anton Feenstra , Zhisheng Huang , and Jaap Heringa . 2024. “Mining Literature and Pathway Data to Explore the Relations of Ketamine With Neurotransmitters and Gut Microbiota Using a Knowledge‐Graph.” Bioinformatics 40: btad771. 10.1093/bioinformatics/btad771 38147362 PMC10769815

[101] Knight, Rob , Alison Vrbanac , Bryn C. Taylor , Alexander Aksenov , Chris Callewaert , Justine Debelius , Antonio Gonzalez , et al. 2018. “Best Practices for Analysing Microbiomes.” Nature Reviews Microbiology 16: 410–422. 10.1038/s41579-018-0029-9 29795328

[102] McLaren, Michael R. , Amy D. Willis , and Benjamin J. Callahan . 2019. “Consistent and Correctable Bias in Metagenomic Sequencing Experiments.” eLife 8: e46923. 10.7554/eLife.46923 31502536 PMC6739870

[103] Jaula Cunanan, Dianne , Timothy Hudson David Culasino Carandang , John David Pilapil , Donmig Jaula Cunanan , Andrea Gail Mollasgo , Gerald Neil S. Manalo , and Gail S. Co , et al. 2026. “Nanopore Sequencing for Microbiological Diagnosis of Bacterial Pneumonia: A Systematic Review and Meta‐Analysis.” European Journal of Clinical Microbiology & Infectious Diseases 45: 1077–1091. 10.1007/s10096-025-05387-z 41499025

[104] Nearing, Jacob T. , Gavin M. Douglas , André M Comeau , and Morgan G. I. Langille . 2018. “Denoising the Denoisers: An Independent Evaluation of Microbiome Sequence Error‐Correction Approaches.” PeerJ 6: e5364. 10.7717/peerj.5364 30123705 PMC6087418

[105] Gevers, Dirk , Rob Knight , Joseph F. Petrosino , Katherine Huang , Amy L. McGuire , Bruce W. Birren , and Karen E. Nelson , et al. 2012. “The Human Microbiome Project: A Community Resource for The Healthy Human Microbiome.” Public Library of Science Biology 10: e1001377. 10.1371/journal.pbio.1001377 PMC3419203 22904687

[106] Gupta, Vinod K. , Sandip Paul , and Chitra Dutta . 2017. “Geography, Ethnicity or Subsistence‐Specific Variations in Human Microbiome Composition and Diversity.” Frontiers in Microbiology 8: 1162. 10.3389/fmicb.2017.01162 28690602 PMC5481955

[107] Zhernakova, Alexandra , Alexander Kurilshikov , Marc Jan Bonder , Ettje F. Tigchelaar , Melanie Schirmer , Tommi Vatanen , Zlatan Mujagic , et al. 2016. “Population‐Based Metagenomics Analysis Reveals Markers for Gut Microbiome Composition and Diversity.” Science 352: 565–569. 10.1126/science.aad3369 27126040 PMC5240844

[108] Forslund, Kristoffer , Falk Hildebrand , Trine Nielsen , Gwen Falony , Emmanuelle Le Chatelier , Shinichi Sunagawa , Edi Prifti , et al. 2015. “Disentangling Type 2 Diabetes and Metformin Treatment Signatures in the Human Gut Microbiota.” Nature 528: 262–266. 10.1038/nature15766 26633628 PMC4681099

[109] Maier, Lisa , Mihaela Pruteanu , Michael Kuhn , Georg Zeller , Anja Telzerow , Exene Erin Anderson , Ana Rita Brochado , et al. 2018. “Extensive Impact of Non‐antibiotic Drugs on Human Gut Bacteria.” Nature 555: 623–628. 10.1038/nature25979 29555994 PMC6108420

[110] Weersma, Rinse K. , Alexandra Zhernakova , and Jingyuan Fu . 2020. “Interaction Between Drugs and the Gut Microbiome.” Gut 69: 1510–1519. 10.1136/gutjnl-2019-320204 32409589 PMC7398478

[111] Poyet, M. , M. Groussin , S. M. Gibbons , J. Avila‐Pacheco , X. Jiang , S. M. Kearney , A. R. Perrotta , et al. 2019. “A Library of Human Gut Bacterial Isolates Paired With Longitudinal Multiomics Data Enables Mechanistic Microbiome Research.” Nature Medicine 25: 1442–1452. 10.1038/s41591-019-0559-3 31477907

[112] Integrative HMP (iHMP) Research Network Consortium . 2014. “The Integrative Human Microbiome Project: Dynamic Analysis of Microbiome‐Host Omics Profiles During Periods of Human Health And Disease.” Cell Host & Microbe 16: 276–289. 10.1016/j.chom.2014.08.014 25211071 PMC5109542

[113] Almeida, Alexandre , Alex L. Mitchell , Miguel Boland , Samuel C. Forster , Gregory B. Gloor , Aleksandra Tarkowska , Trevor D. Lawley , and Robert D. Finn . 2019. “A New Genomic Blueprint of the Human Gut Microbiota.” Nature 568: 499–504. 10.1038/s41586-019-0965-1 30745586 PMC6784870

[114] Abdill, Richard J. , Samantha P. Graham , Vincent Rubinetti , Mansooreh Ahmadian , Parker Hicks , Ashwin Chetty , Daniel McDonald , et al. 2025. “Integration of 168,000 Samples Reveals Global Patterns of the Human Gut Microbiome.” Cell 188: 1100–1118.e17. 10.1016/j.cell.2024.12.017 39848248 PMC11848717

[115] Willis, Amy D. , and David S. Clausen . 2025. “Planning and Describing a Microbiome Data Analysis.” Nature Microbiology 10: 604–607. 10.1038/s41564-025-01944-6 PMC12051388 39984724

[116] Fierer, Noah , Pok Man Leung , Rachael Lappan , Raphael Eisenhofer , Francesco Ricci , Sophie I. Holland , Nicholas Dragone , et al. 2025. “Guidelines for Preventing and Reporting Contamination in Low‐Biomass Microbiome Studies.” Nature Microbiology 10: 1570–1580. 10.1038/s41564-025-02035-2 40542287

[117] Austin, George I. , Aya Brown Kav , Shahd ElNaggar , Heekuk Park , Jana Biermann , Anne‐Catrin Uhlemann , Itsik Pe'er , and Tal Korem . 2025. “Processing‐Bias Correction With DEBIAS‐M Improves Cross‐Study Generalization of Microbiome‐Based Prediction Models.” Nature Microbiology 10: 897–911. 10.1038/s41564-025-01954-4 PMC12087262 40148567

[118] Tseng, Ching‐Hung , and Chun‐Ying Wu . 2025. “From Dysbiosis to Longevity: a Narrative Review into the Gut Microbiome's Impact on Aging.” Journal of Biomedical Science 32: 93. 10.1186/s12929-025-01179-x 41076537 PMC12515389

[119] Zhang, Feng , Rong Li , Yasong Liu , Jinliang Liang , Yihang Gong , Cuicui Xiao , Jianye Cai , et al. 2025. “Integrative Cross‐Tissue Analysis Unveils Complement‐Immunoglobulin Augmentation and Dysbiosis‐Related Fatty Acid Metabolic Remodeling During Mammalian Aging.” iMeta 4: e70027. 10.1002/imt2.70027 40469517 PMC12130579

[120] Nuzum, Nathan D. , Clara Deady , Sarah Kittel‐Schneider , John F. Cryan , Siobhain M. O'Mahony , and Gerard Clarke . 2024. “More Than Just a Number: The Gut Microbiota and Brain Function Across the Extremes of Life.” Gut Microbes 16: 2418988. 10.1080/19490976.2024.2418988 39567371 PMC11583591

[121] Ghosh, Tarini S. , Mrinmoy Das , Ian B. Jeffery , and Paul W. O'Toole . 2020. “Adjusting for Age Improves Identification of Gut Microbiome Alterations in Multiple Diseases.” eLife 9: e50240. 10.7554/eLife.50240 32159510 PMC7065848

[122] Chaudhari, Diptaraj S. , Shalini Jain , Vinod K. Yata , Sidharth P. Mishra , Ambuj Kumar , Amoy Fraser , Judyta Kociolek , et al. 2023. “Unique Trans‐Kingdom Microbiome Structural and Functional Signatures Predict Cognitive Decline in Older Adults.” Geroscience 45: 2819–2834. 10.1007/s11357-023-00799-1 37213047 PMC10643725

[123] Varesi, Angelica , Elisa Pierella , Marcello Romeo , Gaia Bavestrello Piccini , Claudia Alfano , Geir Bjørklund , Abigail Oppong , et al. 2022. “The Potential Role of Gut Microbiota in Alzheimer's Disease: From Diagnosis to Treatment.” Nutrients 14: 668. 10.3390/nu14030668 35277027 PMC8840394

[124] Son, Sang Joon , Dong Yun Lee , Hyun Woong Roh , Maria Ly , Antonija Kolobaric , Howard Aizenstein , Carmen Andreescu , et al. 2025. “Brain Age Mediates Gut Microbiome Dysbiosis‐Related Cognition in Older Adults.” Alzheimer's Research & Therapy 17: 52. 10.1186/s13195-025-01697-8 PMC11866832 40016766

[125] Laske, Christoph , Stephan Müller , Matthias H. J. Munk , Iris Honold , Matthias Willmann , Silke Peter , and Ulrich Schoppmeier . 2024. “Prognostic Value of Gut Microbiome for Conversion From Mild Cognitive Impairment to Alzheimer's Disease Dementia Within 4 Years: Results From the AlzBiom Study.” International Journal of Molecular Sciences 25: 1906. 10.3390/ijms25031906 38339197 PMC10855790

[126] Smith, Patrick , David Willemsen , Miriam Popkes , Franziska Metge , Edson Gandiwa , Martin Reichard , and Dario Riccardo Valenzano . 2017. “Regulation of Life Span by the Gut Microbiota in the Short‐Lived African Turquoise Killifish.” eLife 6: e27014. 10.7554/eLife.27014 28826469 PMC5566455

[127] Yu, Run , Haimeng Zhang , Rui Chen , Yangzhuo Lin , Jingxuan Xu , Ziyang Fang , Yuehang Ru , Chenhan Fan , and Guoqing Wu . 2025. “Fecal Microbiota Transplantation from Methionine‐Restricted Diet Mouse Donors Improves Alzheimer's Learning and Memory Abilities Through Short‐chain Fatty Acids.” Foods 14: 101. 10.3390/foods14010101 39796390 PMC11720665

[128] Olejnik, Piotr , Aleksandra Golenia , and Jolanta Małyszko . 2025. “The Potential Role of Microbiota in Aage‐Related Cognitive Decline: A Narrative Review of the Underlying Molecular Mechanisms.” International Journal of Molecular Sciences 26: 1590. 10.3390/ijms26041590 40004055 PMC11855389

[129] Zhai, Wanfang , Yong Huang , Xuelei Zhang , Wenmin Fei , Yuling Chang , Shasha Cheng , Yi Zhou , et al. 2018. “Profile of the Skin Microbiota in a Healthy Chinese Population.” The Journal of Dermatology 45: 1289–1300. 10.1111/1346-8138.14594 30183092

[130] Li, Xi , Chao Yuan , Licong Xing , and Philippe Humbert . 2017. “Topographical Diversity of Common Skin Microflora and Its Association With Skin Environment Type: An Observational Study in Chinese women.” Scientific Reports 7: 18046. 10.1038/s41598-017-18181-5 29273721 PMC5741767

[131] Chiu, Chung‐Jung , Emily Shiloh Chiu , and Chang Min‐Lee . 2025. “Interaction Between Serum Levels of Porphyromonas Gingivalis Immunoglobulin G and Lutein/zeaxanthin Is Associated With Risk for Age‐Related Macular Degeneration.” Scientific Reports 15: 39400. 10.1038/s41598-025-14144-3 41219247 PMC12606309

[132] Magnusson, Anna , Rongrong Wu , and Isak Demirel . 2024. “Porphyromonas Gingivalis Triggers Microglia Activation and Neurodegenerative Processes Through NOX4.” Frontiers in Cellular and Infection Microbiology 14: 1451683. 10.3389/fcimb.2024.1451683 39469453 PMC11513391

[133] Qin, Lang , Tianyong Sun , Xiao Li , Shigang Zhao , Zheng Liu , Changlong Zhang , Congcong Jin , et al. 2025. “Population‐Level Analyses Identify Host and Environmental Variables Influencing the Vaginal Microbiome.” Signal Transduction and Targeted Therapy 10: 64. 10.1038/s41392-025-02152-8 39966341 PMC11836416

[134] Vivekanandan, Vijitha , Zaiba Hasan Khan , Giriprasad Venugopal , Bhavana Musunuru , Priyanka Mishra , Shalini Srivastava , Balamurugan Ramadass , and Bobban Subhadra . 2024. “VagiBIOM *Lactobacillus* Suppository Improves Vaginal Health Index in Perimenopausal Women With Bacterial Vaginosis: A Randomized Control Trial.” Scientific Reports 14: 3317. 10.1038/s41598-024-53770-1 38336815 PMC10858244

[135] Dwijayanti, Ira , Farah Nuriannisa , Laura Navika Yamani , Catur Wulandari , Fasty Arum Utami , and Trias Mahmudiono . 2025. “Changes in Gut Microbiota Diversity and Composition During Feeding Transitions in Infants: A Scoping Review.” Nutrition 138: 112814. 10.1016/j.nut.2025.112814 40472594

[136] Hollister, Emily B. , Kevin Riehle , Ruth Ann Luna , Erica M. Weidler , Michelle Rubio‐Gonzales , Toni‐Ann Mistretta , Sabeen Raza , et al. 2015. “Structure and Function of the Healthy Pre‐Adolescent Pediatric Gut Microbiome.” Microbiome 3: 36. 10.1186/s40168-015-0101-x 26306392 PMC4550057

[137] Roswall, Josefine , Lisa M. Olsson , Petia Kovatcheva‐Datchary , Staffan Nilsson , Valentina Tremaroli , Marie‐Christine Simon , Pia Kiilerich , et al. 2021. “Developmental Trajectory of the Healthy Human Gut Microbiota During the First 5 Years of Life.” Cell Host & Microbe 29: 765–776. 10.1016/j.chom.2021.02.021 33794185

[138] Zhang, Yusheng , Hong Wang , Yiwei Sang , Mei Liu , Qing Wang , Hongjun Yang , and Xianyu Li . 2024. “Gut Microbiota in Health and Disease: Advances and Future Prospects.” MedComm (2020) 5: e70012. 10.1002/mco2.70012 39568773 PMC11577303

[139] Markle, Janet G. M. , Daniel N. Frank , Steven Mortin‐Toth , Charles E. Robertson , Leah M. Feazel , Ulrike Rolle‐Kampczyk , Martin von Bergen , et al. 2013. “Sex Differences in the Gut Microbiome Drive Hormone‐Dependent Regulation of Autoimmunity.” Science 339: 1084–1088. 10.1126/science.1233521 23328391

[140] Schirmer, Melanie , Eric A. Franzosa , Jason Lloyd‐Price , Lauren J. McIver , Randall Schwager , Tiffany W. Poon , Ashwin N. Ananthakrishnan , et al. 2018. “Dynamics of Metatranscription in the Inflammatory Bowel Disease Gut Microbiome.” Nature Microbiology 3: 337–346. 10.1038/s41564-017-0089-z PMC6131705 29311644

[141] Turnbaugh, Peter J. , Ruth E. Ley , Michael A. Mahowald , Vincent Magrini , Elaine R. Mardis , and Jeffrey I. Gordon . 2006. “An Obesity‐Associated Gut Microbiome With Increased Capacity for Energy Harvest.” Nature 444: 1027–1031. 10.1038/nature05414 17183312

[142] Jiang, Haiyin , Zongxin Ling , Yonghua Zhang , Hongjin Mao , Zhanping Ma , Yan Yin , Weihong Wang , et al. 2015. “Altered Fecal Microbiota Composition in Patients With Major Depressive Disorder.” Brain, Behavior, and Immunity 48: 186–194. 10.1016/j.bbi.2015.03.016 25882912

[143] Clarke, Siobhan F. , Eileen F. Murphy , Orla O'Sullivan , Alice J. Lucey , Margaret Humphreys , Aileen Hogan , Paula Hayes , et al. 2014. “Exercise and Associated Dietary Extremes Impact on Gut Microbial Diversity.” Gut 63: 1913–1920. 10.1136/gutjnl-2013-306541 25021423

[144] Lin, Wei‐Chuan , Cui Zhang , He‐Hua Lei , Zheng Cao , Xin Gao , Wen‐Kai Yu , Xin‐Zhi Li , et al. 2026. “Microbial Keystone Taxa and Metabolic Signatures in Centenarians Regulate Intestinal Homeostasis During Aging.” iMeta 5: e70134. 10.1002/imt2.70134 42491419 PMC13377416

[145] Claesson, Marcus J. , Ian B. Jeffery , Susana Conde , Susan E. Power , Eibhlís M O'Connor , Siobhán Cusack , Hugh M. B. Harris , et al. 2012. “Gut Microbiota Composition Correlates With Diet and Health in the Elderly.” Nature 488: 178–184. 10.1038/nature11319 22797518

[146] Xie, Jiaojiao , Taewan Kim , Zhongmao Liu , Hunter Panier , Suresh Bokoliya , Ming Xu , and Yanjiao Zhou . 2025. “Young Gut Microbiota Transplantation Improves the Metabolic Health of Old Mice.” mSystems 10: e0160124. 10.1128/msystems.01601-24 40444969 PMC12172422

[147] Vich Vila, Arnau , Valerie Collij , Serena Sanna , Trishla Sinha , Floris Imhann , Arno R. Bourgonje , Zlatan Mujagic , et al. 2020. “Impact of Commonly Used Drugs on the Composition and Metabolic Function of the Gut Microbiota.” Nature Communications 11: 362. 10.1038/s41467-019-14177-z PMC6969170 31953381

[148] Bosco, Nabil , and Mario Noti . 2021. “The Aging Gut Microbiome and its Impact on Host Immunity.” Genes & Immunity 22: 289–303. 10.1038/s41435-021-00126-8 33875817 PMC8054695

[149] Górski, Paweł , Adam J. Białas , and Wojciech J. Piotrowski . 2024. “Aging Lung: Molecular Drivers and Impact on Respiratory Diseases‐A Narrative Clinical Review.” Antioxidants 13: 1480. 10.3390/antiox13121480 39765809 PMC11673154

[150] Saeed, Sadia , Jessica Quintin , Hindrik H. D. Kerstens , Nagesha A. Rao , Ali Aghajanirefah , Filomena Matarese , Shih‐Chin Cheng , et al. 2014. “Epigenetic Programming of Monocyte‐to‐Macrophage Differentiation and Trained Innate Immunity.” Science 345: 1251086. 10.1126/science.1251086 25258085 PMC4242194

[151] Shin, Jongoh , Jung‐Ran Noh , Donghui Choe , Namil Lee , Yoseb Song , Suhyung Cho , Eun‐Jung Kang , et al. 2021. “Ageing and Rejuvenation Models Reveal Changes in Key Microbial Communities Associated With Healthy Ageing.” Microbiome 9: 240. 10.1186/s40168-021-01189-5 34906228 PMC8672520

[152] Xia, Mengyu , Junmei Lu , Jiabin Lan , Teng Teng , Rani Shiao , Hongbin Sun , Zheyu Jin , et al. 2025. “Elevated IL‐22 as a Result Of Stress‐induced Gut Leakage Suppresses Septal Neuron Activation to Ameliorate Anxiety‐Like Behavior.” Immunity 58: 218–231.e12. 10.1016/j.immuni.2024.11.008 39644894

[153] Mou, Yi , Yu Du , Lixing Zhou , Jirong Yue , Xianliang Hu , Yixin Liu , Sao Chen , et al. 2022. “Gut Microbiota Interact With the Brain Through Systemic Chronic Inflammation: Implications on Neuroinflammation, Neurodegeneration, and Aging.” Frontiers in Immunology 13: 796288. 10.3389/fimmu.2022.796288 35464431 PMC9021448

[154] Dodiya, Hemraj B. , Christopher B. Forsyth , Robin M. Voigt , Phillip A. Engen , Jinal Patel , Maliha Shaikh , Stefan J. Green , et al. 2020. “Chronic Stress‐Induced Gut Dysfunction Exacerbates Parkinson's Disease Phenotype and Pathology in a Rotenone‐Induced Mouse Model of Parkinson's Disease.” Neurobiology of Disease 135: 104352. 10.1016/j.nbd.2018.12.012 30579705

[155] Needham, Brittany D. , Masanori Funabashi , Mark D. Adame , Zhuo Wang , Joseph C. Boktor , Jillian Haney , Wei‐Li Wu , et al. 2022. “A Gut‐derived Metabolite Alters Brain Activity and Anxiety Behaviour in Mice.” Nature 602: 647–653. 10.1038/s41586-022-04396-8 35165440 PMC9170029

[156] Koizumi, Masayuki , Junko Tatebe , Ippei Watanabe , Junichi Yamazaki , Takanori Ikeda , and Toshisuke Morita . 2014. “Aryl Hydrocarbon Receptor Mediates Indoxyl Sulfate‐induced Cellular Senescence in Human Umbilical Vein Endothelial Cells.” Journal of Atherosclerosis and Thrombosis 21: 904–916. 10.5551/jat.23663 24727683

[157] Ma, Junli , Mingxiao Li , Yiyang Bao , Wenjin Huang , Xiaofang He , Ying Hong , Wenjing Wei , et al. 2024. “Gut Microbiota‐Brain Bile Acid Axis Orchestrates Aging‐related Neuroinflammation and Behavior Impairment in Mice.” Pharmacological Research 208: 107361. 10.1016/j.phrs.2024.107361 39159729

[158] Yano, Jessica M. , Kristie Yu , Gregory P. Donaldson , Gauri G. Shastri , Phoebe Ann , Liang Ma , Cathryn R. Nagler , et al. 2015. “Indigenous Bacteria From the Gut Microbiota Regulate Host Serotonin Biosynthesis.” Cell 161: 264–276. 10.1016/j.cell.2015.02.047 25860609 PMC4393509

[159] Perino, Alessia , Hadrien Demagny , Laura Velazquez‐Villegas , and Kristina Schoonjans . 2021. “Molecular Physiology of Bile Acid Signaling in Health, Disease, and Aging.” Physiological Reviews 101: 683–731. 10.1152/physrev.00049.2019 32790577

[160] Pathak, Preeti , Cen Xie , Robert G. Nichols , Jessica M. Ferrell , Shannon Boehme , Kristopher W. Krausz , Andrew D. Patterson , Frank J. Gonzalez , and John Y. L. Chiang . 2018. “Intestine Farnesoid X Receptor Agonist and the Gut Microbiota Activate G‐Protein Bile Acid Receptor‐1 Signaling to Improve Metabolism.” Hepatology 68: 1574–1588. 10.1002/hep.29857 29486523 PMC6111007

[161] Lu, Jing , Hongyan Wang , Haiyu Zhang , Jiahao Li , Hanqi Li , Qiuyu Chen , Dongyu Han , et al. 2025. “Gut Metabolite Indole‐3‐Propionic Acid Regulates Macrophage Autophagy Through PPT1 Inhibiting Aging‐Related Myocardial Fibrosis.” Advanced Science 12: e01070. 10.1002/advs.202501070 40539882 PMC12442707

[162] Zhang, Yun , Ying Wei , Xuejie Han , Ling Shi , Hui Yu , Xuanrui Ji , Yunlong Gao , et al. 2025. “ *Faecalibacterium prausnitzii* Prevents Age‐Related Heart Failure by Suppressing Ferroptosis in Cardiomyocytes Through Butyrate‐Mediated LCN2 Regulation.” Gut Microbes 17: 2505119. 10.1080/19490976.2025.2505119 40364435 PMC12080280

[163] Chang, Mingyang , Yongqiang Zhou , Tiantian Xia , Pan Shen , Ningning Wang , Chaoji Huangfu , Zhijie Bai , et al. 2025. “Gut Microbiota and OMVs: Unveiling Novel Regulatory Mechanisms in High‐Altitude Myocardial Injury.” iMetaOmics 2: e70001. 10.1002/imo2.70001 41675708 PMC12806435

[164] Chang, Mingyang , Tiantian Xia , Nan Zhang , Qianqian Zhao , Pan Shen , Ningning Wang , Chaoji Huangfu , et al. 2025. “Gut Microbiota and Bacterial Extracellular Vesicles: Emerging Roles in Myocardial Remodelling and Cardiac Health.” Journal of Extracellular Biology 4: e70079. 10.1002/jex2.70079 40791567 PMC12339046

[165] Li, Zhen , Junyue Xing , Xiaohan Ma , Wanjun Zhang , Chuan Wang , Yingying Wang , Xinkun Qi , et al. 2024. “An Orally Administered Bacterial Membrane Protein Nanodrug Ameliorates Doxorubicin Cardiotoxicity Through Alleviating Impaired Intestinal Barrier.” Bioactive Materials 37: 517–532. 10.1016/j.bioactmat.2024.03.027 38698916 PMC11063951

[166] Giron, Muriel , Muriel Thomas , Dominique Dardevet , Christophe Chassard , and Isabelle Savary‐Auzeloux . 2022. “Gut Microbes and Muscle Function: Can Probiotics Make Our Muscles Stronger?” Journal of Cachexia, Sarcopenia and Muscle 13: 1460–1476. 10.1002/jcsm.12964 35278043 PMC9178375

[167] Xie, Xiaoting , and Cong Huang . 2024. “Role of the Gut‐Muscle Axis in Mitochondrial Function of Ageing Muscle Under Different Exercise Modes.” Ageing Research Reviews 98: 102316. 10.1016/j.arr.2024.102316 38703951

[168] Lahiri, Shawon , Hyejin Kim , Isabel Garcia‐Perez , Musarrat Maisha Reza , Katherine A. Martin , Parag Kundu , Laura M. Cox , et al. 2019. “The Gut Microbiota Influences Skeletal Muscle Mass and Function in Mice.” Science Translational Medicine 11: eaan5662. 10.1126/scitranslmed.aan5662 31341063 PMC7501733

[169] Sjögren, Klara , Cecilia Engdahl , Petra Henning , Ulf H. Lerner , Valentina Tremaroli , Marie K. Lagerquist , Fredrik Bäckhed , and Claes Ohlsson . 2012. “The Gut Microbiota Regulates Bone Mass in Mice.” Journal of Bone and Mineral Research 27: 1357–1367. 10.1002/jbmr.1588 22407806 PMC3415623

[170] Henao‐Mejia, Jorge , Eran Elinav , Chengcheng Jin , Liming Hao , Wajahat Z. Mehal , Till Strowig , Christoph A. Thaiss , et al. 2012. “Inflammasome‐Mediated Dysbiosis Regulates Progression of NAFLD and Obesity.” Nature 482: 179–185. 10.1038/nature10809 22297845 PMC3276682

[171] Ananya, Fariha N. , Md Ripon Ahammed , Michael M. Fahem , Sunam Kafle , Mahima Viswanathan , Darshi Desai , Radhika Akku , et al. 2021. “Association of Intestinal Microbial Dysbiosis With Chronic Obstructive Pulmonary Disease.” Cureus 13: e19343. 10.7759/cureus.19343 34909304 PMC8653930

[172] Deng, Xuehui , Bingfeng Lin , Fang Wang , Pingcui Xu , and Nani Wang . 2023. “Specnuezhenide Ameliorates Age‐related Hepatic Lipid Accumulation Via Modulating Bile Acid Homeostasis and Gut Microbiota In D‐galactose‐induced Mice.” Metabolites 13: 960. 10.3390/metabo13080960 37623903 PMC10456809

[173] Ito, Shunsuke , and Masayuki Yoshida . 2014. “Protein‐Bound Uremic Toxins: New Culprits of Cardiovascular Events in Chronic Kidney Disease Patients.” Toxins 6: 665–678. 10.3390/toxins6020665 24561478 PMC3942758

[174] Gao, Ting , Yixuan Li , Xiaoyu Wang , Ran Tao , and Fazheng Ren . 2023. “ *Bifidobacterium longum 68S* Mediated Gut‐Skin Axis Homeostasis Improved Skin Barrier Damage in Aging Mice.” Phytomedicine 120: 155051. 10.1016/j.phymed.2023.155051 37678055

[175] Everard, Amandine , Clara Belzer , Lucie Geurts , Janneke P. Ouwerkerk , Céline Druart , Laure B. Bindels , Yves Guiot , et al. 2013. “Cross‐Talk Between Akkermansia Muciniphila and Intestinal Epithelium Controls Diet‐Induced Obesity.” Proceedings of the National Academy of Sciences 110: 9066–9071. 10.1073/pnas.1219451110 PMC3670398 23671105

[176] Deehan, Edward C. , Chen Yang , Maria Elisa Perez‐Muñoz , K. Nguyen K., Christopher C. Cheng , Lucila Triador , and Zhengxiao Zhang , et al. 2020. “Precision Microbiome Modulation With Discrete Dietary Fiber Structures Directs Short‐Chain Fatty Acid Production.” Cell Host & Microbe 27: 389–404.e6. 10.1016/j.chom.2020.01.006 32004499

[177] Shi, Peng , Simin Xu , Ziling Yang , Liying Wang , Yalun Wu , Ying Li , and Zuobin Zhu . 2024. “Harnessing Gut Microbiota for Longevity: Insights Into Mechanisms and Genetic Manipulation.” iMetaOmics 1: e36. 10.1002/imo2.36 41676117 PMC12806315

[178] Cheng, Hao , Dandan Zhang , Jing Wu , Juan Liu , Yaochuan Zhou , Yuzhu Tan , Wuwen Feng , and Cheng Peng . 2023. “Interactions Between Gut Microbiota and Polyphenols: A Mechanistic and Metabolomic Review.” Phytomedicine 119: 154979. 10.1016/j.phymed.2023.154979 37552899

[179] Cani, Patrice D. , Clara Depommier , Muriel Derrien , Amandine Everard , and Willem M. de Vos . 2022. “ *Akkermansia muciniphila*: Paradigm for Next‐generation Beneficial Microorganisms.” Nature Reviews Gastroenterology & Hepatology 19: 625–637. 10.1038/s41575-022-00631-9 35641786

[180] Yin, Deyi , Li Zhao , Sijing Deng , Yaqi Xie , Kum‐Song Ro , Zeyong Yang , Lei Du , Jingli Xie , and Dongzhi Wei . 2025. “ *Lactiplantibacillus plantarum* X7022 Plays Roles on Aging Mice With Memory Impairment Induced by D‐galactose Through Restoring Neuronal Damage, Relieving Inflammation and Oxidative Stress.” Probiotics and Antimicrobial Proteins 17: 1–14. 10.1007/s12602-023-10208-w 38183568

[181] Liu, Chaoran , Pui Yan Wong , Nilakshi Barua , Baoqi Li , Hei Yuet Wong , Ning Zhang , Simon Kwoon Ho Chow , et al. 2025. “From Clinical to Benchside: *Lacticaseibacillus* and *Faecalibacterium* are Positively Associated With Muscle Health and Alleviate Age‐Related Muscle Disorder.” Aging Cell 24: e14485. 10.1111/acel.14485 39829204 PMC12073917

[182] Dominguez‐Bello, Maria G. , Elizabeth K. Costello , Monica Contreras , Magda Magris , Glida Hidalgo , Noah Fierer , and Rob Knight . 2010. “Delivery Mode Shapes the Acquisition and Structure of the Initial Microbiota Across Multiple Body Habitats in Newborns.” Proceedings of the National Academy of Sciences 107: 11971–11975. 10.1073/pnas.1002601107 PMC2900693 20566857

[183] Holgerson, Pernilla L. , Nelly R. Vestman , Rolf Claesson , Carina Öhman , Magnus Domellöf , Anne C. R. Tanner , Olle Hernell , and Ingegerd Johansson . 2013. “Oral Microbial Profile Discriminates Breast‐Fed From Formula‐Fed Infants.” Journal of Pediatric Gastroenterology and Nutrition 56: 127–136. 10.1097/MPG.0b013e31826f2bc6 22955450 PMC3548038

[184] Socransky, S. S. , A. D. Haffajee , M. A. Cugini , C. Smith , and R. L. Kent Jr. 1998. “Microbial Complexes in Subgingival Plaque.” Journal of Clinical Periodontology 25: 134–144. 10.1111/j.1600-051x.1998.tb02419.x 9495612

[185] Yama, K. , S. Morishima , K. Tsutsumi , R. Jo , Y. Aita , T. Inokuchi , T. Okuda , et al. 2024. “Oral Microbiota Development in the First 60 Months: A Longitudinal Study.” Journal of Dental Research 103: 1249–1257. 10.1177/00220345241272011 39394772 PMC11562288

[186] Crielaard, Wim , Egija Zaura , Annemarie A. Schuller , Susan M. Huse , Roy C. Montijn , and Bart J. F. Keijser . 2011. “Exploring the Oral Microbiota Of Children at Various Developmental Stages of Their Dentition in the Relation to Their Oral Health.” BMC Medical Genomics 4: 22. 10.1186/1755-8794-4-22 21371338 PMC3058002

[187] Mason, Matthew R. , Stephanie Chambers , Shareef M. Dabdoub , Sarat Thikkurissy , and Purnima S. Kumar . 2018. “Characterizing Oral Microbial Communities Across Dentition States and Colonization Niches.” Microbiome 6: 67. 10.1186/s40168-018-0443-2 29631628 PMC5891995

[188] Willis, Jesse R. , Ester Saus , Susana Iraola‐Guzmán , Ewa Ksiezopolska , Luca Cozzuto , Luis A. Bejarano , Nuria Andreu‐Somavilla , et al. 2022. “Citizen‐Science Reveals Changes in the Oral Microbiome in Spain Through Age and Lifestyle Factors.” npj Biofilms Microbiomes 8: 38. 10.1038/s41522-022-00279-y 35585074 PMC9117221

[189] Burcham, Zachary M. , Nicole L. Garneau , Sarah S. Comstock , Robin M. Tucker , Rob Knight , Jessica L. Metcalf , Brian Reinhart , et al.(Genetics of Taste Lab Citizen Scientists) . 2020. “Patterns of Oral Microbiota Diversity in Adults And Children: A Crowdsourced Population Study.” Scientific Reports 10: 2133. 10.1038/s41598-020-59016-0 32034250 PMC7005749

[190] Lloyd‐Price, Jason , Anup Mahurkar , Gholamali Rahnavard , Jonathan Crabtree , Joshua Orvis , A Brantley Hall , Arthur Brady , et al. 2017. “Strains, Functions and Dynamics in the Expanded Human Microbiome Project.” Nature 550: 61–66. 10.1038/nature23889 28953883 PMC5831082

[191] Rosier, B. T. , N. Takahashi , E. Zaura , B. P. Krom , R. M. MartÍnez‐Espinosa , S. G. J. van Breda , P. D. Marsh , and A. Mira . 2022. “The Importance of Nitrate Reduction for Oral Health.” Journal of Dental Research 101: 887–897. 10.1177/00220345221080982 35196931

[192] Marsh, Philip D. 2006. “Dental Plaque as a Biofilm and a Microbial Community: Implications for Health and Disease.” BMC Oral Health 6: S14. 10.1186/1472-6831-6-S1-S14 16934115 PMC2147593

[193] Zawadzki, Paweł J , Konrad Perkowski , Marcin Padzik , Elżbieta Mierzwińska‐Nastalska , Jacek P. Szaflik , David Bruce Conn , and Lidia Chomicz . 2017. “Examination of Oral Microbiota Diversity in Adults and Older Adults as an Approach to Prevent Spread of Risk Factors for Human Infections.” BioMed Research International 2017: 8106491. 10.1155/2017/8106491 29082256 PMC5610830

[194] Flores, Gilberto E. , J. Gregory Caporaso , Jessica B. Henley , Jai Ram Rideout , Daniel Domogala , John Chase , Jonathan W. Leff , et al. 2014. “Temporal Variability Is a Personalized Feature of the Human Microbiome.” Genome Biology 15: 531. 10.1186/s13059-014-0531-y 25517225 PMC4252997

[195] Sekundo, Caroline , Eva Langowski , Diana Wolff , Sébastien Boutin , and Cornelia Frese . 2022. “Maintaining Oral Health for a Hundred Years and More? ‐ An Analysis of Microbial and Salivary Factors in a Cohort of Centenarians.” Journal of Oral Microbiology 14: 2059891. 10.1080/20002297.2022.2059891 35401946 PMC8986295

[196] Shi, Baochen , Michaela Chang , John Martin , Makedonka Mitreva , Renate Lux , Perry Klokkevold , Erica Sodergren , et al. 2015. “Dynamic Changes in the Subgingival Microbiome and Their Potential for Diagnosis and Prognosis of Periodontitis.” mBio 6: e01926–14. 10.1128/mBio.01926-14 25691586 PMC4337560

[197] Hajishengallis, G. , and R. J. Lamont . 2012. “Beyond the Red Complex and into More Complexity: The Polymicrobial Synergy and Dysbiosis (PSD) Model of Periodontal Disease Etiology.” Molecular Oral Microbiology 27: 409–419. 10.1111/j.2041-1014.2012.00663.x 23134607 PMC3653317

[198] D'Aiuto, F. , M. Parkar , G. Andreou , J. Suvan , P. M. Brett , D. Ready , and M. S. Tonetti . 2004. “Periodontitis and Systemic Inflammation: Control of the Local Infection Is Associated With a Reduction in Serum Inflammatory Markers.” Journal of Dental Research 83: 156–160. 10.1177/154405910408300214 14742655

[199] Schenkein, Harvey A. , Panos N. Papapanou , Robert Genco , and Mariano Sanz . 2020. “Mechanisms Underlying the Association Between Periodontitis and Atherosclerotic Disease.” Periodontology 2000 83: 90–106. 10.1111/prd.12304 32385879

[200] Franceschi, Claudio , Massimiliano Bonafè , Silvana Valensin , Fabiola Olivieri , Maria De Luca , Enzo Ottaviani , and Giovanna De Benedictis . 2000. “Inflamm‐Aging. An Evolutionary Perspective on Immunosenescence.” Annals of the New York Academy of Sciences 908: 244–254. 10.1111/j.1749-6632.2000.tb06651.x 10911963

[201] Tran, Andrew H. , Abbas H. Zaidi , Ann F. Bolger , Oscar H. Del Brutto , Rashmi Hegde , Lauren L. Patton , Jamie Rausch , et al. 2025. “Periodontal Disease and Atherosclerotic Cardiovascular Disease: A Scientific Statement From the American Heart Association.” Circulation 153: e73–e88. 10.1161/CIR.0000000000001390 41399933

[202] El‐Awady, Ahmed R. , Mahmoud Elashiry , Ana C. Morandini , Mohamed M. Meghil , and Christopher W. Cutler . 2022. “Dendritic Cells a Critical Link to Alveolar Bone Loss and Systemic Disease Risk in Periodontitis: Immunotherapeutic Implications.” Periodontology 2000 89: 41–50. 10.1111/prd.12428 35244951 PMC9018591

[203] Stöhr, Julia , Janett Barbaresko , Manuela Neuenschwander , and Sabrina Schlesinger . 2021. “Bidirectional Association Between Periodontal Disease and Diabetes Mellitus: A Systematic Review and Meta‐Analysis of Cohort Studies.” Scientific Reports 11: 13686. 10.1038/s41598-021-93062-6 34211029 PMC8249442

[204] Huang, Mingming , Xinbi Zhang , Leiming, Yi , and Zheng Di . 2025. “Advances in the Study of Oral Microbiota in Association With T2DM: A Systematic Review.” Frontiers in Cellular and Infection Microbiology 15: 1629304. 10.3389/fcimb.2025.1629304 41104128 PMC12521273

[205] Liu, Sixin , Stuart G. Dashper , and Rui Zhao . 2023. “Association Between Oral Bacteria and Alzheimer's Disease: A Systematic Review and Meta‐analysis.” Journal of Alzheimer's Disease 91: 129–150. 10.3233/JAD-220627 36404545

[206] Beydoun, May A. , Hind A. Beydoun , Sharmin Hossain , Ziad W. El‐Hajj , Jordan Weiss , and Alan B. Zonderman . 2020. “Clinical and Bacterial Markers of Periodontitis and Their Association With Incident All‐Cause and Alzheimer's Disease Dementia in a Large National Survey.” Journal of Alzheimer's Disease 75: 157–172. 10.3233/JAD-200064 PMC11008556 32280099

[207] Walford, R. L. 1964. “The Immunologic Theory of Aging.” The Gerontologist 4: 195–197. 10.1093/geront/4.4.195 14289265

[208] Perricone, Carlo , Fulvia Ceccarelli , Saccucci Matteo , Gabriele Di Carlo , Dimitrios P. Bogdanos , Ramona Lucchetti , Andrea Pilloni , et al. 2019. “ *Porphyromonas gingivalis* and Rheumatoid Arthritis.” Current Opinion in Rheumatology 31: 517–524. 10.1097/bor.0000000000000638 31268867

[209] Zhao, Yanfang , Zhaofei Li , Lingkai Su , Andre Ballesteros‐Tato , Jannet Katz , Suzanne M. Michalek , Xu Feng , and Ping Zhang . 2020. “Frontline Science: Characterization and Regulation of Osteoclast Precursors Following Chronic Porphyromonas Gingivalis Infection.” Journal of Leukocyte Biology 108: 1037–1050. 10.1002/jlb.1hi0620-230r 33463750

[210] Zhang, Xuan , Dongya Zhang , Huijue Jia , Qiang Feng , Donghui Wang , Di Liang , Xiangni Wu , et al. 2015. “The Oral and Gut Microbiomes Are Perturbed in Rheumatoid Arthritis and Partly Normalized After Treatment.” Nature Medicine 21: 895–905. 10.1038/nm.3914 26214836

[211] Mieszkowska, Anna , Magdalena Marcinkowska , Magdalena Widziolek , Jan Potempa , and Magdalena Chadzinska . 2025. “ *Porphromonas gingivalis* Infection Induces Gingipain‐Dependent Changes in the Brain Vasculature of Zebrafish Larvae.” Cell Communication and Signaling 23: 552. 10.1186/s12964-025-02557-6 41310669 PMC12751225

[212] Hao, Chunbo , Rui Chen , Zhen Fan , and Shan Wang . 2026. “Corrigendum to “AIM2‐Mediated Senescence of Gingival Fibroblasts Exacerbates Inflammaging in Periodontitis.” Free Radical Biology and Medicine 251: 544. 10.1016/j.freeradbiomed.2026.04.137 41456811

[213] Xie, M. , X. Huang , Q. Tang , S. Yu , K. Zhang , X. Lu , Y. Zhang , et al. 2025. “Porphyromonas Gingivalis Impairs Microglial Aβ Clearance in a Mouse Model.” Journal of Dental Research 104: 408–418. 10.1177/00220345241294009 39953680

[214] de Freitas, Laura , Giovana Calixto , Marlus Chorilli , Juçaíra Giusti , Vanderlei Bagnato , Nikolaos Soukos , Mansoor Amiji , and Carla Fontana . 2016. “Polymeric Nanoparticle‐Based Photodynamic Therapy For Chronic Periodontitis *In Vivo* .” International Journal of Molecular Sciences 17: 769. 10.3390/ijms17050769 27213356 PMC4881588

[215] Dominy, Stephen S. , Casey Lynch , Florian Ermini , Malgorzata Benedyk , Agata Marczyk , Andrei Konradi , Mai Nguyen , et al. 2019. “ *Porphyromonas gingivalis* in Alzheimer's Disease Brains: Evidence for Disease Causation and Treatment With Small‐Molecule Inhibitors.” Science Advances 5: eaau3333. 10.1126/sciadv.aau3333 30746447 PMC6357742

[216] Minić, Ivan , Ana Pejčić , and Marija Bradić‐Vasić . 2022. “Effect of the Local Probiotics in the Therapy of Periodontitis a Randomized Prospective Study.” International Journal of Dental Hygiene 20: 401–407. 10.1111/idh.12509 33964104

[217] Golletz, Patrick , Sissel Damsbo Jensen , Madeline Collignon , Charles Hall , Amanda Batoul Khamas , Andreas Møllebjerg , Sebastian Schlafer , Rikke Louise Meyer , and Karolina Tykwinska . 2025. “Coaggregation of Oral Pathogens by Postbiotic Lactobacilli.” Journal of Oral Microbiology 17: 2508483. 10.1080/20002297.2025.2508483 40453788 PMC12123943

[218] Eick, Sigrun , and Adrian Lussi . 2021. “Arginine: A Weapon Against Cariogenic Biofilm?” Monographs in Oral Science 29: 80–90. 10.1159/000510203 33427222

[219] Rosier, B. T. , E. Buetas , E. M. Moya‐Gonzalvez , A. Artacho , and Alex Mira . 2020. “Nitrate as a Potential Prebiotic for the Oral Microbiome.” Scientific Reports 10: 12895. 10.1038/s41598-020-69931-x 32732931 PMC7393384

[220] Vanhatalo, Anni , Joanna E. L'Heureux , James Kelly , Jamie R. Blackwell , Lee J. Wylie , Jonathan Fulford , Paul G. Winyard , et al. 2021. “Network Analysis of Nitrate‐Sensitive Oral Microbiome Reveals Interactions With Cognitive Function and Cardiovascular Health Across Dietary Interventions.” Redox Biology 41: 101933. 10.1016/j.redox.2021.101933 33721836 PMC7970425

[221] Vanhatalo, Anni , Jamie R. Blackwell , Joanna E. L'Heureux , David W. Williams , Ann Smith , Mark van der Giezen , Paul G. Winyard , James Kelly , and Andrew M. Jones . 2018. “Nitrate‐Responsive Oral Microbiome Modulates Nitric Oxide Homeostasis and Blood Pressure in Humans.” Free Radical Biology and Medicine 124: 21–30. 10.1016/j.freeradbiomed.2018.05.078 29807159 PMC6191927

[222] Katz, Joseph , and Isabel Garcia . 2025. “The Use of Chlorhexidine Mouthwash and Diagnosis of Primary Hypertension in a Large Hospital Cohort.” Quintessence International 56: 138–142. 10.3290/j.qi.b5872795 39639848

[223] Joshipura, Kaumudi , Francisco Muñoz‐Torres , Jeanpaul Fernández‐Santiago , Rakesh P. Patel , and Angel Lopez‐Candales . 2020. “Over‐the‐Counter Mouthwash Use, Nitric Oxide and Hypertension Risk.” Blood Pressure 29: 103–112. 10.1080/08037051.2019.1680270 31709856 PMC7125030

[224] Sikora, Ewa , Anna Bielak‐Zmijewska , and Grazyna Mosieniak . 2019. “Targeting Normal and Cancer Senescent Cells as a Strategy of Senotherapy.” Ageing Research Reviews 55: 100941. 10.1016/j.arr.2019.100941 31408714

[225] Nath, Sonia , Peter Zilm , Lisa Jamieson , Kostas Kapellas , Nirmal Goswami , Kevin Ketagoda , and Laura S. Weyrich . 2021. “Development and Characterization of an Oral Microbiome Transplant Among Australians for the Treatment of Dental Caries and Periodontal Disease: A Study Protocol.” PLoS One 16: e0260433. 10.1371/journal.pone.0260433 34843568 PMC8629173

[226] Bamashmous, Shatha , Georgios A. Kotsakis , Kristopher A. Kerns , Brian G. Leroux , Camille Zenobia , Dandan Chen , Harsh M. Trivedi , Jeffrey S. McLean , and Richard P. Darveau . 2021. “Human Variation in Gingival Inflammation.” Proceedings of the National Academy of Sciences of the United States of America 118: e2012578118. 10.1073/pnas.2012578118 34193520 PMC8271746

[227] Wang, Zhiyan , Fengming Yuan , Xuewei Zhong , Shi Feng , and Tao Song . 2025. “Skin Microbiome and Skin Aging: Emerging Strategies for Manipulation.” Microbiological Research 300: 128285. 10.1016/j.micres.2025.128285 40716140

[228] Duarte, Marco , Sílvia Santos Pedrosa , P Raaj Khusial , and Ana Raquel Madureira . 2024. “Exploring the Interplay Between Stress Mediators and Skin Microbiota in Shaping Age‐Related Hallmarks: A Review.” Mechanisms of Ageing and Development 220: 111956. 10.1016/j.mad.2024.111956 38906383

[229] Swaney, Mary Hannah , Duncan J. Newman , Junhong Mao , Anthony C. Hilton , Tony Worthington , and Min Li . 2025. “Aging‐Dependent Skin Microbiome Alterations Across Body Sites in a United Kingdom Cohort.” Frontiers in Aging 6: 1644012. 10.3389/fragi.2025.1644012 41048701 PMC12491275

[230] Suri, Hitakshika , Harshika Suri , Nachiket Nagda , Toshika Misra , and Suneetha Vuppu . 2025. “Current Perspectives on the Human Skin Microbiome: Functional Insights and Strategies for Therapeutic Modulation.” Biomedicine & Pharmacotherapy 193: 118655. 10.1016/j.biopha.2025.118655 41172954

[231] Hong, Ji Yeon , Doyeon Kwon , and Kui Young Park . 2025. “Microbiome‐Based Interventions for Skin Aging and Barrier Function: A Comprehensive Review.” Annals of Dermatology 37: 259–268. 10.5021/ad.25.009 41044805 PMC12505367

[232] Mueller, Noel T. , Moira K. Differding , Haipeng Sun , Jincheng Wang , Shira Levy , Varsha Deopujari , Lawrence J. Appel , et al. 2023. “Maternal Bacterial Engraftment in Multiple Body Sites of Cesarean Section Born Neonates After Vaginal Seeding‐A Randomized Controlled Trial.” mBio 14: e0049123. 10.1128/mbio.00491-23 37074174 PMC10294643

[233] Rapin, Alexis , Eva Maria Rehbinder , Matthew Macowan , Céline Pattaroni , Karin C. Lødrup Carlsen , Nicola L. Harris , Christine M. Jonassen , et al. 2023. “The Skin Microbiome in the First Year of Life and Its Association With Atopic Dermatitis.” Allergy 78: 1949–1963. 10.1111/all.15671 36779606

[234] Baker, Jacob S. , Evan Qu , Christopher P. Mancuso , A Delphine Tripp , Arolyn Conwill , and Tami D. Lieberman . 2025. “Intraspecies Dynamics Underlie the Apparent Stability of Two Important Skin Microbiome Species.” Cell Host & Microbe 3: 643–656.e7. 10.1016/j.chom.2025.04.010 PMC12084124 40315837

[235] Park, Jin , Nicole H. Schwardt , Jay‐Hyun Jo , Zhiwei Zhang , Valentina Pillai , Sheila Phang , Sheila M. Brady , et al. 2022. “Shifts in the Skin Bacterial and Fungal Communities of Healthy Children Transitioning Through Puberty.” Journal of Investigative Dermatology 142: 212–219. 10.1016/j.jid.2021.04.034 34252398 PMC8688298

[236] Schneider, Andrea M. , Zachary T. Nolan , Kalins Banerjee , Allison R. Paine , Zhaoyuan Cong , Samantha L. Gettle , Amy L. Longenecker , et al. 2023. “Evolution of the Facial Skin Microbiome During Puberty in Normal and Acne Skin.” Journal of the European Academy of Dermatology and Venereology 37: 166–175. 10.1111/jdv.18616 36165604 PMC11134479

[237] Howard, Brian , Charles C. Bascom , Ping Hu , Robert L. Binder , Gina Fadayel , Tom G. Huggins , Bradley B. Jarrold , et al. 2022. “Aging‐Associated Changes in the Adult Human Skin Microbiome and the Host Factors that Affect Skin Microbiome Composition.” Journal of Investigative Dermatology 142: 1934–1946.e21. 10.1016/j.jid.2021.11.029 34890626

[238] Challa, Varun , Santosh Kumar Prajapati , Surabhi Gangani , Dhananjay Yadav , Lalitha Lekkala , Shalini Jain , and Hariom Yadav . 2025. “Microbiome‐Aging‐Wrinkles Axis of Skin: Molecular Insights and Microbial Interventions.” International Journal of Molecular Sciences 26: 10022. 10.3390/ijms262010022 41155313 PMC12564825

[239] Garlet, Allison , Valerie Andre‐Frei , Nicolas Del Bene , Hunter James Cameron , Anita Samuga , Vimal Rawat , Philipp Ternes , and Sabrina Leoty‐Okombi . 2024. “Facial Skin Microbiome Composition and Functional Shift With Aging.” Microorganisms 12: 1021. 10.3390/microorganisms12051021 38792850 PMC11124346

[240] Cheng, Mingyue , Hong Zhou , Haobo Zhang , Xinchao Zhang , Shuting Zhang , Hong Bai , Yugo Zha , et al. 2024. “Hidden Links Between Skin Microbiome and Skin Imaging Phenome.” Genomics Proteomics Bioinformatics 22: qzae040. 10.1093/gpbjnl/qzae040 39436239 PMC11849492

[241] He, Huaming , Mingren Xiao , Liya Song , Yan Tian , and Yan Jia . 2026. “Lipidome‐Microbiome Crosstalk as an Outer Niche in the Skin: Regulatory Networks in Health and Disease.” British Journal of Dermatology 194: 195–204. 10.1093/bjd/ljaf353 41078307

[242] Wojciechowska, Katarzyna , and Katarzyna Dos Santos Szewczyk . 2025. “The Skin Microbiome and Bioactive Compounds: Mechanisms of Modulation, Dysbiosis, and Dermatological Implications.” Molecules 30: 4363. 10.3390/molecules30224363 41302420 PMC12655691

[243] Alwan, Wisam , and Paola Di Meglio . 2021. “Guardians of the Barrier: Microbiota Engage AHR in Keratinocytes to Maintain Skin Homeostasis.” Cell Host & Microbe 29: 1213–1216. 10.1016/j.chom.2021.07.007 34384523

[244] Almoughrabie, Samia , Laura Cau , Kellen Cavagnero , Alan M. O'Neill , Fengwu Li , Andrea Roso‐Mares , Carine Mainzer , et al. 2023. “Commensal *Cutibacterium acnes* Induce Epidermal Lipid Synthesis Important for Skin Barrier Function.” Science Advances 9: eadg6262. 10.1126/sciadv.adg6262 37595033 PMC10438445

[245] Zheng, Yue , Rachelle L. Hunt , Amer E. Villaruz , Emilie L. Fisher , Ryan Liu , Qian Liu , Gordon Y. C. Cheung , Min Li , and Michael Otto . 2022. “Commensal *Staphylococcus* Epidermidis Contributes to Skin Barrier Homeostasis by Generating Protective Ceramides.” Cell Host & Microbe 30: 301–313. 10.1016/j.chom.2022.01.004 35123653 PMC8917079

[246] Khalil, Sylvia B. , and Caitlin H. Kowalski . 2026. “The Cutaneous Microbiome: Microbial Remodeling of the Skin Lipid Landscape.” mSphere 10: e0064825. 10.1128/msphere.00648-25 PMC13317201 42267822

[247] Kim, Han Bi , Helen Alexander , Ji Young Um , Bo Young Chung , Chun Wook Park , Carsten Flohr , and Hye One Kim . 2025. “Skin Microbiome Dynamics in Atopic Dermatitis: Understanding Host‐Microbiome Interactions.” Allergy, Asthma & Immunology Research 17: 165–180. 10.4168/aair.2025.17.2.165 PMC11982640 40204503

[248] Dhariwala, Miqdad O. , Ricardo O. Carale , Andrea M. DeRogatis , Henry Rodriguez‐Valbuena , Joy N. Okoro , Christina A. Ekstrand , Antonin Weckel , et al. 2026. “Commensal‐Myeloid Crosstalk in Neonatal Skin Regulates Interleukin‐1 Signaling and Cutaneous Type 17 Inflammation.” Immunity 59: 403–418.e7. 10.1016/j.immuni.2026.01.005 41616767 PMC12908917

[249] Liu, Siqi , Flávio Silva Costa , and Dario Riccardo Valenzano . 2026. “Immune Surveillance and Microbial Escape in the Aging Host: Why Does the Microbiome Lose Its Balance?” PLoS Biology 24: e3003815. 10.1371/journal.pbio.3003815 42160397 PMC13211257

[250] Rees, Nia Paddison , Jessica Conway , Ben Dugan , Sayeda S. Amir , Aimee Parker , Simon R. Carding , and Niharika A. Duggal . 2025. “Defining Microbiota‐Derived Metabolite Butyrate as a Senomorphic: Therapeutic Potential in the Age‐Related T Cell Senescence.” Aging Cell 24: e70257. 10.1111/acel.70257 41201238 PMC12686592

[251] Gribonika, Inta , Victor I. Band , Liang Chi , Paula Juliana Perez‐Chaparro , Verena M. Link , Eduard Ansaldo , Cihan Oguz , et al. 2025. “Publisher Correction: Skin Autonomous Antibody Production Regulates Host–microbiota Interactions.” Nature 639: E6. 10.1038/s41586-025-08776-8 39972149 PMC11882448

[252] Cheng, Jialing , Guo Bao , Demin Lin , Hongliang Wang , Yanfang Yang , Youbai Chen , Meiying Ning , Jun Ye , and Yuling Liu . 2026. “Silk Fibroin Counteracts Fibroblast Senescence to Restore Ecm Homeostasis in Aged Skin.” Bioactive Materials 58: 666–684. 10.1016/j.bioactmat.2025.12.006 41551197 PMC12808896

[253] Yang, Fan , Mingxuan Li , Yao Zuo , Lei Zhang , Jinyong Wu , Zhi Liu , and Hua Wang . 2025. “ *In Vitro* Anti‐Aging Effects of Yeast/Rice Fermentation Filtrate Combined With Sialic Acid in Cosmetic Applications.” Antioxidants 14: 1184. 10.3390/antiox14101184 41154493 PMC12561143

[254] Cheviti, Bharat , Andrew Cawley , Shruti Malviya , Barry Murphy , Gouri Malhotra , Thanumalayan K. Sivaram , Simone Sethna , et al. 2025. “Mild Skin Cleansers Strengthen Microbiome Networks Without Affecting the Skin Microbiome.” British Journal of Dermatology 193(Suppl 2): ii40–ii50. 10.1093/bjd/ljaf282 41118317

[255] Brooks, Sarah G. , Rami H. Mahmoud , Rachel R. Lin , Joachim W. Fluhr , and Gil Yosipovitch . 2025. “The Skin Acid Mantle: An Update on Skin pH.” Journal of Investigative Dermatology 145: 509–521. 10.1016/j.jid.2024.07.009 39243251

[256] Fatima, Munazza , Hiba Omer , Nadia Nadeem , and Saira Imtiaz . 2026. “Cosmetics and Their Role in Modulating Skin Microbiome Health. A Narrative Review.” Journal of the Pakistan Medical Association 76: 417–423. 10.47391/JPMA.30352 42017723

[257] Mercer, Steven D. , Andrew J. McBain , and Catherine O'Neill . 2025. “The Skin Microbiome, Microbial Metabolites and the Epidermal Response to Ultraviolet Radiation‐Towards Next Generation Suncare.” Experimental Dermatology 34: e70142. 10.1111/exd.70142 40704429 PMC12287992

[258] Oh, Sae Woong , Eunbi Yu , Kitae Kwon , Hye Ja Lee , Hyun Sook Yeom , Kyung Man Hahm , Jin Oh Park , Jae Youl Cho , and Jongsung Lee . 2025. “An AAQPR Peptide From Aspergillus oryzae‐Fermented Wheat Peptone Promotes The Regenerative Potential of Dermal and Epidermal Layers of the Skin in *In Vitro* Assays and Clinical Trials.” Biomaterials Science 13: 4264–4282. 10.1039/d5bm00571j 40576611

[259] Zhang, Jingting , Guangyu Liu , Yi Yang , Yi Xie , Linyan Yao , and Jianxi Xiao . 2025. “Functionalized Transdermal Collagen Mimetic Peptides for Efficient Repair of Subacute Aged Skin.” Biomaterials Science 13: 5495–5511. 10.1039/d5bm00940e 40828418

[260] Kim, Kwang H. , Yusook Chung , Ji‐Won Huh , Dong Jin Park , Yejin Cho , Yeseul Oh , Haengdueng Jeong , et al. 2022. “Gut Microbiota of the Young Ameliorates Physical Fitness of the Aged in Mice.” Microbiome 10: 238. 10.1186/s40168-022-01386-w 36567320 PMC9791737

[261] Wei, Simin , Weicheng Tang , Dan Chen , Jiaqiang Xiong , Liru Xue , Yun Dai , Yican Guo , et al. 2024. “Multiomics Insights into the Female Reproductive Aging.” Ageing Research Reviews 95: 102245. 10.1016/j.arr.2024.102245 38401570

[262] Liu, Yuanyuan , and Jinmin Gao . 2023. “Reproductive Aging: Biological Pathways and Potential Interventive Strategies.” Journal of Genetics and Genomics 50: 141–150. 10.1016/j.jgg.2022.07.002 35840100

[263] Dong, Lu , Daniel Boon Loong Teh , Brian Keith Kennedy , and Zhongwei Huang . 2023. “Unraveling Female Reproductive Senescence to Enhance Healthy Longevity.” Cell Research 33: 11–29. 10.1038/s41422-022-00718-7 36588114 PMC9810745

[264] Liu, Xingyu , Yuanqu Zhao , Yanzhi Feng , Shixuan Wang , and Jinjin Zhang . 2025. “Ovarian Aging: Mechanisms, Age‐Related Disorders, and Therapeutic Interventions.” MedComm (2020) 6: e70481. 10.1002/mco2.70481 41256733 PMC12620568

[265] Zhang, Jia‐Le , Meng Lv , Chao‐Fan Yang , Ying‐Xi Zhu , and Chao‐Jun Li . 2023. “Mevalonate Pathway and Male Reproductive Aging.” Molecular Reproduction and Development 90: 774–781. 10.1002/mrd.23705 37733694

[266] Stone, Bronte A. , Allyse Alex , Lawrence B. Werlin , and Richard P. Marrs . 2013. “Age Thresholds for Changes in Semen Parameters in Men.” Fertility and Sterility 100: 952–958. 10.1016/j.fertnstert.2013.05.046 23809502

[267] Cox, C. M. , M. E. Thoma , N. Tchangalova , G. Mburu , M. J. Bornstein , C. L. Johnson , and J. Kiarie . 2022. “Infertility Prevalence and the Methods of Estimation From 1990 to 2021: A Systematic Review and Meta‐Analysis.” Human Reproduction Open 2022: hoac051. 10.1093/hropen/hoac051 36483694 PMC9725182

[268] Driscoll, Anne K. , and Brady E. Hamilton . 2025. “Effects of Age‐Specific Fertility Trends on Overall Fertility Trends: United States, 1990–2023.” National Vital Statistics Reports 6: 1. 10.15620/cdc/174576 PMC12434873 40911793

[269] Beaujouan, Eva . 2020. “Latest‐Late Fertility? Decline and Resurgence of Late Parenthood Across the Low‐Fertility Countries.” Population and Development Review 46: 219–247. 10.1111/padr.12334 32733116 PMC7384131

[270] Khadilkar, Jharna Mahajan Bhanushali , P Mahto Anuradha , and Satish Khadilkar . 2026. “Cognition in Menopausal Women.” International Journal of Gynaecology and Obstetrics 27. 10.1002/ijgo.70944 PMC13505283 41902393

[271] Veldhuis, Johannes D. 2008. “Aging and Hormones of the Hypothalamo‐Pituitary Axis: Gonadotropic Axis in Men and Somatotropic Axes in Men and Women.” Ageing Research Reviews 7: 189–208. 10.1016/j.arr.2007.12.005 18343203 PMC2583117

[272] Dai, Ruoxi , Wen Xu , Wei Chen , Liyuan Cui , Lisha Li , Jing Zhou , Xueling Jin , et al. 2022. “Epigenetic Modification of Kiss1 Gene Expression in the AVPV is Essential for Female Reproductive Aging.” BioScience Trends 16: 346–358. 10.5582/bst.2022.01358 36273897

[273] Liao, Chung‐Chih , I Lee Chun , and Miao Li Jung . 2025. “Exploring the Genetically Predicted Gut Microbiota–reproductive Axis: Impact on Anti‐Müllerian Hormone Levels.” International Journal of Gynecology & Obstetrics 172: 336–342. 10.1002/ijgo.70380 40679133

[274] Xiong, Yan , Xiaoxue Lu , Bohao Li , Shiyao Xu , Beibei Fu , Zhou Sha , Rong Tian , et al. 2025. “Bacteroides Fragilis Transplantation Reverses Reproductive Senescence by Transporting Extracellular Vesicles Through the Gut‐Ovary Axis.” Advanced Science 12: e2409740. 10.1002/advs.202409740 39805029 PMC11884595

[275] Consortium, Human Microbiome Project . 2012. “Structure, Function and Diversity of the Healthy Human Microbiome.” Nature 486: 207–214. 10.1038/nature11234 22699609 PMC3564958

[276] Cao, Wenli , Xiayan Fu , Jing Zhou , Qing Qi , Feijun Ye , Lisha Li , and Ling Wang . 2023. “The Effect of the Female Genital Tract and Gut Microbiome on Reproductive Dysfunction.” BioScience Trends 17: 458–474. 10.5582/bst.2023.01133 38104979

[277] Zhu, Bin , Zhi Tao , Laahirie Edupuganti , Myrna G. Serrano , and Gregory A. Buck . 2022. “Roles of the Microbiota of the Female Reproductive Tract in Gynecological and Reproductive Health.” Microbiology and Molecular Biology Reviews 86: e0018121. 10.1128/mmbr.00181-21 36222685 PMC9769908

[278] Alfano, Massimo , Roberto Ferrarese , Irene Locatelli , Eugenio Ventimiglia , Silvia Ippolito , Pierangela Gallina , Daniela Cesana , et al. 2018. “Testicular Microbiome in Azoospermic Men‐First Evidence of the Impact of an Altered Microenvironment.” Human Reproduction 33(7): 1212–1217. 10.1093/humrep/dey116 29850857 PMC6012977

[279] Lundy, Scott D. , Naseer Sangwan , Neel V. Parekh , Manesh Kumar Panner Selvam , Sajal Gupta , Peter McCaffrey , Kovi Bessoff , et al. 2021. “Functional and Taxonomic Dysbiosis of the Gut, Urine, and Semen Microbiomes in Male Infertility.” European Urology 79: 826–836. 10.1016/j.eururo.2021.01.014 33573862

[280] Koedooder, Rivka , Shari Mackens , Andries Budding , Damiat Fares , Christophe Blockeel , Joop Laven , and Sam Schoenmakers . 2018. “Identification and Evaluation of the Microbiome in the Female and Male Reproductive Tracts.” Human Reproduction Update 25: 298–325. 10.1093/humupd/dmy048 30938752

[281] Tuddenham, Susan , Jacques Ravel , and Jeanne M. Marrazzo . 2021. “Protection and Risk: Male and Female Genital Microbiota and Sexually Transmitted Infections.” The Journal of Infectious Diseases 223: S222–S235. 10.1093/infdis/jiaa762 33576776 PMC8206802

[282] Zozaya, Marcela , Michael J. Ferris , Julia D. Siren , Rebecca Lillis , Leann Myers , M Jacques Nsuami , A Murat Eren , et al. 2016. “Bacterial Communities in Penile Skin, Male Urethra, and Vaginas of Heterosexual Couples With and Without Bacterial Vaginosis.” Microbiome 4: 16. 10.1186/s40168-016-0161-6 27090518 PMC4835890

[283] Mändar, Reet , Margus Punab , Natalja Borovkova , Eleri Lapp , Riinu Kiiker , Paul Korrovits , Andres Metspalu , et al. 2015. “Complementary Seminovaginal Microbiome in Couples.” Research in Microbiology 166: 440–447. 10.1016/j.resmic.2015.03.009 25869222

[284] Jenkins, Dominick J. , Benjamin M. Woolston , M Indriati Hood‐Pishchany , Paula Pelayo , Alyssa N. Konopaski , M. Quinn Peters , Michael T. France , et al. 2023. “Bacterial Amylases Enable Glycogen Degradation by the Vaginal Microbiome.” Nature Microbiology 8: 1641–1652. 10.1038/s41564-023-01447-2 PMC10465358 37563289

[285] Yamamoto, Ted , Xia Zhou , Chris J. Williams , Anne Hochwalt , and Larry J. Forney . 2009. “Bacterial Populations in the Vaginas of Healthy Adolescent Women.” Journal of Pediatric & Adolescent Gynecology 22: 11–18. 10.1016/j.jpag.2008.01.073 19232297

[286] Gajer, Pawel , Rebecca M. Brotman , Guoyun Bai , Joyce Sakamoto , Ursel M. E. Schütte , Xue Zhong , Sara S. K. Koenig , et al. 2012. “Temporal Dynamics of the Human Vaginal Microbiota.” Science Translational Medicine 4: 132ra52. 10.1126/scitranslmed.3003605 PMC3722878 22553250

[287] Sparvoli, Luiz G. , Ramon V. Cortez , Silvia Daher , Marina Padilha , Sue Y. Sun , Mary U. Nakamura , and Carla R. Taddei . 2020. “Women's Multisite Microbial Modulation During Pregnancy.” Microbial Pathogenesis 147: 104230. 10.1016/j.micpath.2020.104230 32428665

[288] Alonzo Martínez, Melanie C. , Eduardo Cazorla , Esther Cánovas , Juan F. Martínez‐Blanch , Empar Chenoll , Eric Climent , and Vicente Navarro‐López . 2021. “Study of the Vaginal Microbiota in Healthy Women of Reproductive Age.” Microorganisms 9: 1069. 10.3390/microorganisms9051069 34063526 PMC8156707

[289] Santacroce, Luigi , Raffaele Palmirotta , Lucrezia Bottalico , Ioannis Alexandros Charitos , Marica Colella , Skender Topi , and Emilio Jirillo . 2023. “Crosstalk Between the Resident Microbiota and the Immune Cells Regulates Female Genital Tract Health.” Life 13: 1531. 10.3390/life13071531 37511906 PMC10381428

[290] Lebeer, Sarah , Sarah Ahannach , Thies Gehrmann , Stijn Wittouck , Tom Eilers , Eline Oerlemans , Sandra Condori , et al. 2023. “A Citizen‐Science‐Enabled Catalogue of the Vaginal Microbiome and Associated Factors.” Nature Microbiology 8: 2183–2195. 10.1038/s41564-023-01500-0 PMC10627828 37884815

[291] Lee, Christina Y. , Lillian R. Dillard , Jason A. Papin , and Kelly B. Arnold . 2023. “New Perspectives Into the Vaginal Microbiome With Systems Biology.” Trends in Microbiology 31: 356–368. 10.1016/j.tim.2022.09.011 36272885

[292] Brotman, Rebecca M. , Michelle D. Shardell , Pawel Gajer , Doug Fadrosh , Kathryn Chang , Michelle I. Silver , Raphael P. Viscidi , et al. 2018. “Association Between the Vaginal Microbiota, Menopause Status, and Signs of Vulvovaginal Atrophy.” Menopause 25: 1321–1330. 10.1097/gme.0000000000001236 30358729

[293] Lin, Wenyu , Liying Wang , Binhua Dong , Yuhang Zhang , Yan Zhang , Liang Wang , Jun Shen , et al. 2025. “Age‐Related Vaginal Microecology and Infection Epidemiology Among Premenopausal and Postmenopausal Gynecologic Outpatients: A Cross‐Sectional Study.” Frontiers in Cellular and Infection Microbiology 15: 1569667. 10.3389/fcimb.2025.1569667 40831702 PMC12358402

[294] Chatzokou, Dimitra , Ermioni Tsarna , Efstathia Davouti , Charalampos S. Siristatidis , Smaragdi Christopoulou , Nikolaos Spanakis , Athanasios Tsakris , and Panagiotis Christopoulos . 2025. “Semen Microbiome, Male Infertility, and Reproductive Health.” International Journal of Molecular Sciences 26: 1446. 10.3390/ijms26041446 40003912 PMC11854939

[295] Jendraszak, Magdalena , Izabela Skibińska , Małgorzata Kotwicka , and Mirosław Andrusiewicz . 2024. “The Elusive Male Microbiome: Revealing the Link Between the Genital Microbiota and Fertility. Critical Review and Future Perspectives.” Critical Reviews in Clinical Laboratory Sciences 61: 559–587. 10.1080/10408363.2024.2331489 38523477

[296] Nelson, David E. , Qunfeng Dong , Barbara Van der Pol , Evelyn Toh , Baochang Fan , Barry P. Katz , Deming Mi , et al. 2012. “Bacterial Communities of the Coronal Sulcus and Distal Urethra of Adolescent Males.” PLoS One 7: e36298. 10.1371/journal.pone.0036298 22606251 PMC3350528

[297] Mehta, Supriya D. , Debarghya Nandi , Walter Agingu , Stefan J. Green , Fredrick O. Otieno , Dulal K. Bhaumik , and Robert C. Bailey . 2022. “Longitudinal Changes in the Composition of the Penile Microbiome are Associated With Circumcision Status, HIV and HSV‐2 Status, Sexual Practices, and Female Partner Microbiome Composition.” Frontiers in Cellular and Infection Microbiology 12: 916437. 10.3389/fcimb.2022.916437 35865819 PMC9294230

[298] Kaufman, Jean‐Marc , Bruno Lapauw , Ahmed Mahmoud , Guy T'Sjoen , and Ilpo Tapani Huhtaniemi . 2019. “Aging and the Male Reproductive System.” Endocrine Reviews 40: 906–972. 10.1210/er.2018-00178 30888401

[299] Qin, Junjie , Xulian Shi , Junming Xu , Simin Yuan , Bo Zheng , Enpu Zhang , Guixiao Huang , et al. 2021. “Characterization of the Genitourinary Microbiome of 1,165 Middle‐Aged and Elderly Healthy Individuals.” Frontiers in Microbiology 12: 673969. 10.3389/fmicb.2021.673969 34489882 PMC8417382

[300] Bao, Binghao , Haolang Wen , Fei Wang , Daishu Han , and Baoxing Liu . 2025. “Inflammation of the Male Reproductive System: Clinical Aspects and Mechanisms.” Frontiers in Endocrinology 16: 1547020. 10.3389/fendo.2025.1547020 40607213 PMC12213417

[301] Martinez, Maria Sol , and Ruben Dario Motrich . 2025. “Macrophages in the Male Genital Tract.” Biomedical Journal 25: 100910. 10.1016/j.bj.2025.100910 40865908

[302] Schaeffer, Anthony J. , and Lindsay E. Nicolle . 2016. “Urinary Tract Infections in Older Men.” New England Journal of Medicine 374: 562–571. 10.1056/NEJMcp1503950 27248641

[303] Mason, C. Y. , A. Sobti , and A. L. Goodman . 2023. “ *Staphylococcus aureus* Bacteriuria: Implications and Management.” Journal of Antimicrobial Chemotherapy: Antimicrobial Resistance 5: dlac123. 10.1093/jacamr/dlac123 PMC9833284 36644414

[304] Koeijers, J. J. , A. Verbon , A. G. H. Kessels , A. Bartelds , G. Donkers , S. Nys , and E. E. Stobberingh . 2010. “Urinary Tract Infection in Male General Practice Patients: Uropathogens and Antibiotic Susceptibility.” Urology 76: 336–340. 10.1016/j.urology.2010.02.052 20494416

[305] Ughade, Prachi A. , Deepti Shrivastava , and Kamlesh Chaudhari . 2024. “Navigating the Microbial Landscape: Understanding Dysbiosis in Human Genital Tracts and its Impact on Fertility.” Cureus 16: e67040. 10.7759/cureus.67040 39286717 PMC11403153

[306] Zhou, Jiedong , Shian Hu , Yong Ouyang , Yucheng Kong , and Min Liu . 2025. “Immunometabolism and Male Reproductive Function: Linking Inflammation, Oxidative Stress, And Declining Fertility.” Frontiers in Immunology 16: 1736492. 10.3389/fimmu.2025.1736492 41445731 PMC12722913

[307] Pan, Melody , and Cristian O'Flaherty . 2026. “Gut Microbial Dysbiosis and Male Reproductive Health: Current Insights and Future Directions.” International Journal of Molecular Sciences 27: 4482. 10.3390/ijms27104482 42196459 PMC13207464

[308] Chen, Zi‐Feng , Yi‐Feng Shen , Da‐Wei Gao , Deng‐Feng Lin , Wen‐Zhe Ma , and De‐Gui Chang . 2025. “Metabolic Pathways and Male Fertility: Exploring the Role of Sertoli Cells in Energy Homeostasis and Spermatogenesis.” American Journal of Physiology‐Endocrinology and Metabolism 329: E160–E178. 10.1152/ajpendo.00074.2025 40522874

[309] Lliberos, Carolina , Seng H. Liew , Pirooz Zareie , Nicole L. La Gruta , Ashley Mansell , and Karla Hutt . 2021. “Evaluation of Inflammation and Follicle Depletion During Ovarian Ageing in Mice.” Scientific Reports 11: 278. 10.1038/s41598-020-79488-4 33432051 PMC7801638

[310] Lliberos, Carolina , Seng H. Liew , Ashley Mansell , and Karla J. Hutt . 2020. “The inflammasome Contributes to Depletion of the Ovarian Reserve During Aging in Mice.” Frontiers in Cell and Developmental Biology 8: 628473. 10.3389/fcell.2020.628473 33644037 PMC7905095

[311] Moustakli, Efthalia , Sofoklis Stavros , Periklis Katopodis , Anastasios Potiris , Peter Drakakis , Stefanos Dafopoulos , Athanasios Zachariou , et al. 2025. “Gut Microbiome Dysbiosis and Its Impact on Reproductive Health: Mechanisms and Clinical Applications.” Metabolites 15: 390. 10.3390/metabo15060390 40559414 PMC12195147

[312] Ashonibare, Victory J. , Bolaji A. Akorede , Precious J. Ashonibare , Tunmise M. Akhigbe , and Roland Eghoghosoa Akhigbe . 2024. “Gut Microbiota‐Gonadal Axis: The Impact of Gut Microbiota on Reproductive Functions.” Frontiers in Immunology 15: 1346035. 10.3389/fimmu.2024.1346035 38482009 PMC10933031

[313] Hoffmann, Alexander , Genhong Cheng , and David Baltimore . 2025. “NF‐κB: Master Regulator of Cellular Responses in Health and Disease.” Immunity & Inflammation 1: 2. 10.1007/s44466-025-00014-0 41669117 PMC12883533

[314] Fang, Su , Tianming Wang , Yuanyuan Li , Haoyu Xue , Juan Zou , Jingyi Cai , Rong Shi , Jiasheng Wu , and Yueming Ma . 2022. “Gardenia Jasminoides Ellis Polysaccharide Ameliorates Cholestatic Liver Injury by Alleviating Gut Microbiota Dysbiosis and Inhibiting the TLR4/NF‐κB Signaling Pathway.” International Journal of Biological Macromolecules 205: 23–36. 10.1016/j.ijbiomac.2022.02.056 35176320

[315] Zhang, Hong , Heng Zou , Chanyu Zhang , and Shen Zhang . 2024. “Chronic Endometritis and the Endometrial Microbiota: Implications for Reproductive Success in Patients With Recurrent Implantation Failure.” Annals of Clinical Microbiology and Antimicrobials 23: 49. 10.1186/s12941-024-00710-6 38816832 PMC11140900

[316] Shen, Jialu , Shuai Xu , Qingyu Zhao , Junmin Zhang , and Huiyan Zhang . 2026. “β‐Glucuronidase at the Microbiota—Host Interface: Dual Regulatory Roles and Precision Modulation by Natural Products.” Molecules 31: 601. 10.3390/molecules31040601 41752380 PMC12943147

[317] Huang, Feiling , Ying Cao , Jinghui Liang , Ruiyi Tang , Si Wu , Peng Zhang , and Rong Chen . 2024. “The Influence of the Gut Microbiome on Ovarian Aging.” Gut Microbes 16: 2295394. 10.1080/19490976.2023.2295394 38170622 PMC10766396

[318] Geng, Lulu , Xin Yang , Jiani Sun , Ximing Ran , Dan Zhou , Mingming Ye , Li Wen , Ruirui Wang , and Miaoxin Chen . 2025. “Gut Microbiota Modulation by Inulin Improves Metabolism and Ovarian Function in Polycystic Ovary Syndrome.” Advanced Science 12: e2412558. 10.1002/advs.202412558 40192074 PMC12120758

[319] Lv, Shuya , Jingrong Huang , Yadan Luo , Yuhang Wen , Baoting Chen , Hao Qiu , Huanxin Chen , et al. 2024. “Gut Microbiota is Involved in Male Reproductive Function: A Review.” Frontiers in Microbiology 15: 1371667. 10.3389/fmicb.2024.1371667 38765683 PMC11099273

[320] Jin, Zirun , Yuzhuo Yang , Yalei Cao , Qi Wen , Yu Xi , Jianxing Cheng , Qiancheng Zhao , et al. 2023. “The Gut Metabolite 3‐Hydroxyphenylacetic Acid Rejuvenates Spermatogenic Dysfunction in Aged Mice Through GPX4‐Mediated Ferroptosis.” Microbiome 11: 212. 10.1186/s40168-023-01659-y 37752615 PMC10523725

[321] Dubey, Itishree , Nandheeswari, Vigneshwaran , Gourav Rohilla , Lalruatmawii, Pratik Naxine , Mahesh Rachamalla , and Sapana Kushwaha . 2024. “Exploring the Hypothetical Links Between Environmental Pollutants, Diet, and the Gut‐testis Axis: The Potential Role of Microbes in Male Reproductive Health.” Reproductive Toxicology 130: 108732. 10.1016/j.reprotox.2024.108732 39395506

[322] Ignatiuk, Vasilina M. , Viktoria S. Sharova , and Liudmila A. Zakharova . 2025. “The Role of Cytokines in the Development and Functioning of the Hypothalamic‐Pituitary‐Gonadal Axis in Mammals In Normal and Pathological Conditions.” International Journal of Molecular Sciences 26: 11057. 10.3390/ijms262211057 41303539 PMC12652693

[323] Yan, Ting , Ruiyu Wang , Jingfei Yao , and Minmin Luo . 2023. “Single‐Cell Transcriptomic Analysis Reveals Rich Pituitary‐immune Interactions Under Systemic Inflammation.” PLoS Biology 21: e3002403. 10.1371/journal.pbio.3002403 38109308 PMC10727439

[324] Xiao, Liwen , Zhenqiang Zuo , and Fangqing Zhao . 2024. “Microbiome in Female Reproductive Health: Implications for Fertility and Assisted Reproductive Technologies.” Genomics Proteomics Bioinformatics 22: qzad005. 10.1093/gpbjnl/qzad005 38862423 PMC11104452

[325] Hiratsuka, Daiki , Mitsunori Matsuo , and Yasushi Hirota . 2026. “The Reproductive Tract Microbiome and Female Fertility: Dysbiosis, Disease Links, and Emerging Therapeutic Strategies.” Fertility and Sterility 125: 574–582. 10.1016/j.fertnstert.2026.01.010 41577072

[326] De Seta, Francesco , Giuseppina Campisciano , Nunzia Zanotta , Giuseppe Ricci , and Manola Comar . 2019. “The Vaginal Community State Types Microbiome‐immune Network as Key Factor for Bacterial VaGinosis and Aerobic Vaginitis.” Frontiers in Microbiology 10: 2451. 10.3389/fmicb.2019.02451 31736898 PMC6831638

[327] Swidsinski, Sonja , Wiltrud Maria Moll , and Alexander Swidsinski . 2023. “Bacterial Vaginosis‐Vaginal Polymicrobial Biofilms and Dysbiosis.” Deutsches Ärzteblatt International 120: 347–354. 10.3238/arztebl.m2023.0090 37097068 PMC10412922

[328] Sobel, Jack D. 2025. “Biofilm in Bacterial Vaginosis: A Legitimate Therapeutic Challenge?” The Journal of Infectious Diseases 231: 40–43. 10.1093/infdis/jiae135 38680028

[329] Motrich, Ruben D. , Florencia C. Salazar , Maria L. Breser , Juan P. Mackern‐Oberti , Gloria J. Godoy , Carolina Olivera , Daniela A. Paira , and Virginia E. Rivero . 2018. “Implications of Prostate Inflammation on Male Fertility.” Andrologia 50: e13093. 10.1111/and.13093 30569650

[330] Ihsan, Awais Ullah , Farhan Ullah Khan , Puregmaa Khongorzul , Khalil Ali Ahmad , Muhammad Naveed , Sufia Yasmeen , Yanfang Cao , et al. 2018. “Role of Oxidative Stress in Pathology of Chronic Prostatitis/chronic Pelvic Pain Syndrome and Male Infertility and Antioxidants Function in Ameliorating Oxidative Stress.” Biomedicine & Pharmacotherapy 106: 714–723. 10.1016/j.biopha.2018.06.139 29990863

[331] Garcia‐Segura, Sergio , Javier del Rey , Laia Closa , Iris Garcia‐Martínez , Carlos Hobeich , Ana Belén Castel , Francisco Vidal , et al. 2022. “Seminal Microbiota of Idiopathic Infertile Patients and Its Relationship With Sperm DNA Integrity.” Frontiers in Cell and Developmental Biology 10: 937157. 10.3389/fcell.2022.937157 35837328 PMC9275566

[332] Kuribayashi, Sohei , Noopur Naik , Aaron W. Miller , and Scott D. Lundy . 2026. “Micro‐Management: How the Male Reproductive Microbiome Shapes Male Fertility.” Fertility and Sterility 125: 583–595. 10.1016/j.fertnstert.2026.01.019 41611115

[333] Agarwal, Ashok , Neel Parekh , Manesh Kumar Panner Selvam , Ralf Henkel , Rupin Shah , Sheryl T. Homa , Ranjith Ramasamy , et al. 2019. “Male Oxidative Stress Infertility (MOSI): Proposed Terminology And Clinical Practice Guidelines for Management of Idiopathic Male Infertility.” The World Journal of Men's Health 37: 296–312. 10.5534/wjmh.190055 PMC6704307 31081299

[334] Yang, Meina , Shu Wen , Jing Zhang , Jin Peng , Xiaoyang Shen , and Liangzhi Xu . 2022. “Systematic Review and Meta‐Analysis: Changes of Gut Microbiota Before and After Menopause.” Disease Markers 2022: 3767373. 10.1155/2022/3767373 35923245 PMC9343210

[335] Baker, James M. , Layla Al‐Nakkash , and Melissa M. Herbst‐Kralovetz . 2017. “Estrogen‐Gut Microbiome Axis: Physiological and Clinical Implications.” Maturitas 103: 45–53. 10.1016/j.maturitas.2017.06.025 28778332

[336] Fan, Yong , and Oluf Pedersen . 2021. “Gut Microbiota in Human Metabolic Health and Disease.” Nature Reviews Microbiology 19: 55–71. 10.1038/s41579-020-0433-9 32887946

[337] Moore, Kate H. , Samantha Ognenovska , Xin‐Yi Chua , Zhuoran Chen , Chloe Hicks , Fatima El‐Assaad , Nevine Te West , and Emad El‐Omar . 2024. “Change in Microbiota Profile After Vaginal Estriol Cream in Postmenopausal Women With Stress Incontinence.” Frontiers in Microbiology 15: 1302819. 10.3389/fmicb.2024.1302819 38505551 PMC10948564

[338] Chakraborty, Binita , Jovita Byemerwa , Taylor Krebs , Felicia Lim , Ching‐Yi Chang , and Donald P. McDonnell . 2023. “Estrogen Receptor Signaling in the Immune System.” Endocrine Reviews 44: 117–141. 10.1210/endrev/bnac017 35709009

[339] Santos‐Marcos, Jose Antonio , Marina Mora‐Ortiz , Manuel Tena‐Sempere , Jose Lopez‐Miranda , and Antonio Camargo . 2023. “Interaction Between Gut Microbiota and Sex Hormones and Their Relation to Sexual Dimorphism in Metabolic Diseases.” Biology of Sex Differences 14: 4. 10.1186/s13293-023-00490-2 36750874 PMC9903633

[340] DeLeon, Orlando , and Eugene B. Chang . 2025. “Assessing the Health of the Gut Microbial Organ: Why and How?” Journal of Clinical Investigation 135: e184313. 10.1172/jci184313 40454478 PMC12126215

[341] Mann, Elizabeth R. , Ying Ka Lam , and Holm H. Uhlig . 2024. “Short‐Chain Fatty Acids: Linking Diet, the Microbiome and Immunity.” Nature Reviews Immunology 24: 577–595. 10.1038/s41577-024-01014-8 38565643

[342] Chen, Bandy , and Laurent Gautron . 2025. “Gut‐Derived Lipopolysaccharides and Metabolic Endotoxemia: A Critical Review.” American Journal of Physiology‐Endocrinology and Metabolism 329: E746–E754. 10.1152/ajpendo.00355.2025 41083214

[343] Li, Xia , Chentao Li , Wanying Zhang , Yanan Wang , Pengxu Qian , and He Huang . 2023. “Inflammation and Aging: Signaling Pathways and Intervention Therapies.” Signal Transduction and Targeted Therapy 8: 239. 10.1038/s41392-023-01502-8 37291105 PMC10248351

[344] Mukhopadhya, Indrani , and Petra Louis . 2025. “Gut Microbiota‐Derived Short‐Chain Fatty Acids and Their Role in Human Health and Disease.” Nature Reviews Microbiology 23: 635–651. 10.1038/s41579-025-01183-w 40360779

[345] Neyrinck, Audrey M. , Julie Rodriguez , Zhengxiao Zhang , Benjamin Seethaler , Cándido Robles Sánchez , Martin Roumain , Sophie Hiel , et al. 2021. “Prebiotic Dietary Fibre Intervention Improves Fecal Markers Related to Inflammation in Obese Patients: Results From the Food4Gut Randomized Placebo‐Controlled Trial.” European Journal of Nutrition 60: 3159–3170. 10.1007/s00394-021-02484-5 33544206 PMC8354918

[346] Bailey, Melisa A. , Sharon V. Thompson , Annemarie R. Mysonhimer , Jessica N. Bennett , James J. Vanhie , Michael De Lisio , Nicholas A. Burd , Naiman A. Khan , and Hannah D. Holscher . 2023. “Dietary Fiber Intake and Fecal Short‐Chain Fatty Acid Concentrations Are Associated With Lower Plasma Lipopolysaccharide‐Binding Protein and Inflammation.” American Journal of Physiology‐Gastrointestinal and Liver Physiology 324: G369–G377. 10.1152/ajpgi.00176.2021 36791082

[347] Lv, Jing , Shengkai Jin , Yuwei Zhang , Yuhua Zhou , Menglu Li , and Ninghan Feng . 2024. “Equol: A Metabolite of Gut Microbiota With Potential Antitumor Effects.” Gut Pathogens 16: 35. 10.1186/s13099-024-00625-9 38972976 PMC11229234

[348] Kumari, Nikki , Rashmi Kumari , Ankita Dua , Mona Singh , Roushan Kumar , Poonam Singh , Susan Duyar‐Ayerdi , et al. 2024. “From Gut to Hormones: Unraveling The Role of Gut Microbiota in (Phyto)estrogen Modulation in Health and Disease.” Molecular Nutrition & Food Research 68: 2300688. 10.1002/mnfr.202300688 38342595

[349] Sillen, Mart , Sarah Lebeer , and Patrick Van Dijck . 2025. “Through Thick and Thin: The Vaginal Microbiome as Both Occupant and Healer.” PLoS Pathogens 21: e1013346. 10.1371/journal.ppat.1013346 40694551 PMC12282852

[350] Yang, Jiali , Mengyun Peng , Shaochong Tan , Shengchan Ge , Li Xie , Tonghai Zhou , Wei Liu , et al. 2023. “Calcium Tungstate Microgel Enhances the Delivery and Colonization of Probiotics During Colitis Via Intestinal Ecological Niche Occupancy.” ACS Central Science 9: 1327–1341. 10.1021/acscentsci.3c00227 37521784 PMC10375893

[351] Pendharkar, Sonal , Axel Skafte‐Holm , Gizem Simsek , and Thor Haahr . 2023. “ *Lactobacilli* and Their Probiotic Effects in the Vagina of Reproductive Age Women.” Microorganisms 11: 636. 10.3390/microorganisms11030636 36985210 PMC10056154

[352] Argentini, Chiara , Federico Fontana , Giulia Alessandri , Gabriele Andrea Lugli , Leonardo Mancabelli , Maria Cristina Ossiprandi , Douwe van Sinderen , et al. 2022. “Evaluation of Modulatory Activities of *Lactobacillus crispatus* Strains in the Context of the Vaginal Microbiota.” Microbiology Spectrum 10: e0273321. 10.1128/spectrum.02733-21 35266820 PMC9045136

[353] Rezazadeh, Mahnaz Boroumand , Minoo Zanganeh , Lida Jarahi , and Zahra Fatehi . 2024. “Comparative Efficacy of Oral and Vaginal Probiotics in Reducing the Recurrence of Bacterial Vaginosis: A Double‐Bblind Clinical Trial.” BMC Women's Health 24: 575. 10.1186/s12905-024-03418-z 39462408 PMC11515199

[354] Murjani, Karan , Dharmisha Solanki , and Vijai Singh . 2026. “Recombinant Live Biotherapeutics and Synthetic Biology: Recent Advancement and Perspective.” Progress in Molecular Biology and Translational Science 220: 65–86. 10.1016/bs.pmbts.2026.01.004 41714087

[355] Farahani, Linda , Tharu Tharakan , Tet Yap , Jonathan W. Ramsay , Channa N. Jayasena , and Suks Minhas . 2021. “The Semen Microbiome and Its Impact on Sperm Function and Male Fertility: A Systematic Review and Meta‐Analysis.” Andrology 9: 115–144. 10.1111/andr.12886 32794312

[356] DeFilipp, Zachariah , Patricia P. Bloom , Mariam Torres Soto , Michael K. Mansour , Mohamad R. A. Sater , Miriam H. Huntley , Sarah Turbett , et al. 2019. “Drug‐rResistant *E. coli Bacteremia* Transmitted by Fecal Microbiota Transplant.” New England Journal of Medicine 381: 2043–2050. 10.1056/NEJMoa1910437 31665575

[357] Khoruts, Alexander , and Michael J. Sadowsky . 2016. “Understanding the Mechanisms of Faecal Microbiota Transplantation.” Nature Reviews Gastroenterology & Hepatology 13: 508–516. 10.1038/nrgastro.2016.98 27329806 PMC5909819

[358] Boehme, Marcus , Katherine E. Guzzetta , Thomaz F. S. Bastiaanssen , Marcel van de Wouw , Gerard M. Moloney , Andreu Gual‐Grau , Simon Spichak , et al. 2021. “Microbiota From Young Mice Counteracts Selective Age‐associated Behavioral Deficits.” Nature Aging 1: 666–676. 10.1038/s43587-021-00093-9 37117767

[359] Parker, Aimée , Stefano Romano , Rebecca Ansorge , Asmaa Aboelnour , Gwenaelle Le Gall , George M. Savva , Matthew G. Pontifex , et al. 2022. “Fecal Microbiota Transfer Between Young and Aged Mice Reverses Hallmarks of the Aging Gut, Eye, and Brain.” Microbiome 10: 68. 10.1186/s40168-022-01243-w 35501923 PMC9063061

[360] Wrønding, Tine , Kilian Vomstein , Elleke F. Bosma , Brynjulf Mortensen , Henrik Westh , Julie Elm Heintz , Sarah Mollerup , et al. 2023. “Antibiotic‐Free Vaginal Microbiota Transplant With Donor Engraftment, Dysbiosis Resolution and Live Birth After Recurrent Pregnancy Loss: A Proof Of Concept Case Study.” eClinicalMedicine 61: 102070. 10.1016/j.eclinm.2023.102070 37528843 PMC10388571

[361] Luo, Haiqin , Chuhui Zhou , Lepeng Zhou , Yan He , and Ri‐Hua Xie . 2024. “The Effectiveness of Vaginal Microbiota Transplantation for Vaginal Dysbiosis and Bacterial Vaginosis: A Scoping Review.” Archives of Gynecology and Obstetrics 310: 643–653. 10.1007/s00404-024-07611-1 38914708

[362] Mehta, Supriya D. 2026. “Role of the Penile Microbiome in Female Sex Partner Risk of Bacterial Vaginosis and Sexually Transmitted Infections: A Narrative Review.” Clinical Microbiology Reviews 39: e0033125. 10.1128/cmr.00331-25 PMC13560657 42233652

[363] Vodstrcil, Lenka A. , Erica L. Plummer , Christopher K. Fairley , Jane S. Hocking , Matthew G. Law , Kathy Petoumenos , Deborah Bateson , et al. 2025. “Male‐Partner Treatment to Prevent Recurrence of Bacterial Vaginosis.” New England Journal of Medicine 392: 947–957. 10.1056/NEJMoa2405404 40043236

[364] Molina, Nerea M. , Analuce Canha‐Gouveia , Irene Leonés‐Baños , Alberto Sola‐Leyva , Eva Vargas , Susana Ruíz‐Durán , Celia M. Tenorio , et al. 2025. “The Complementary Seminovaginal Microbiome In Health and Disease.” Reproductive BioMedicine Online 50: 104707. 10.1016/j.rbmo.2024.104707 40174296

[365] Lin, Feiyun , Lin Ma , and Zhumei Sheng . 2025. “Health Disorders in Menopausal Women: Microbiome Alterations, Associated Problems, and Possible Treatments.” BioMedical Engineering OnLine 24: 84. 10.1186/s12938-025-01415-3 40624665 PMC12235801

[366] Ticinesi, Andrea , Stefania Maggi , Antonio Nouvenne , Giovanni Zuliani , and Claudio Franceschi . 2026. “The Gut Microbiome and Ageing Trajectories: Mechanisms and Clinical Implications.” Nature Reviews Endocrinology 22: 323–337. 10.1038/s41574-026-01236-x 41776008

[367] Schulze‐Späte, Ulrike , Ludwig Wurschi , Emiel P. C. van der Vorst , Frank Hölzle , Rogerio B. Craveiro , Michael Wolf , and Heidi Noels . 2024. “Crosstalk Between Periodontitis and Cardiovascular Risk.” Frontiers in Immunology 15: 1469077. 10.3389/fimmu.2024.1469077 39717783 PMC11663742

[368] Fu, Y. D. , C. L. Li , C. L. Hu , M. D. Pei , W. Y. Cai , Y. Q. Li , Lang Xu , and Yan Zeng . 2024. “Meta Analysis of the Correlation Between Periodontal Health and Cognitive Impairment in the Older Population.” The Journal of Prevention of Alzheimer's Disease 11: 1307–1315. 10.14283/jpad.2024.87 39350376

[369] Ghosh, Tarini Shankar , Simone Rampelli , Ian B. Jeffery , Aurelia Santoro , Marta Neto , Miriam Capri , Enrico Giampieri , et al. 2020. “Mediterranean Diet Intervention Alters the Gut Microbiome in Older People Reducing Frailty and Improving Health Status: The NU‐AGE 1‐Year Dietary Intervention Across Five European Countries.” Gut 69: 1218–1228. 10.1136/gutjnl-2019-319654 32066625 PMC7306987

[370] Zmora, Niv , Gili Zilberman‐Schapira , Jotham Suez , Uria Mor , Mally Dori‐Bachash , Stavros Bashiardes , Eran Kotler , et al. 2018. “Personalized Gut Mucosal Colonization Resistance to Empiric Probiotics Is Associated With Unique Host And Microbiome Features.” Cell 174: 1388–1405.e21. 10.1016/j.cell.2018.08.041 30193112

[371] Li, Simone S. , Ana Zhu , Vladimir Benes , Paul I. Costea , Rajna Hercog , Falk Hildebrand , Jaime Huerta‐Cepas , et al. 2016. “Durable Coexistence of Donor and Recipient Strains After Fecal Microbiota Transplantation.” Science 352: 586–589. 10.1126/science.aad8852 27126044

[372] Youngster, Ilan , Jenny Sauk , Christina Pindar , Robin G. Wilson , Jess L. Kaplan , Mark B. Smith , Eric J. Alm , et al. 2014. “Fecal Microbiota Transplant for Relapsing *Clostridium difficile* Infection Using a Frozen Inoculum From Unrelated Donors: A Randomized, Open‐label, Controlled Pilot Study.” Clinical Infectious Diseases 58: 1515–1522. 10.1093/cid/ciu135 24762631 PMC4017893

[373] Chaudhary, Sakshi , Pardeep Kaur , Thokchom Arjun Singh , Kaniz Shahar Bano , Ashish Vyas , Alok Kumar Mishra , Prabhakar Singh , and Mohammad Murtaza Mehdi . 2024. “The Dynamic Crosslinking Between Gut Microbiota and Inflammation During Aging: Reviewing the Nutritional and Hormetic Approaches Against Dysbiosis and Inflammaging.” Biogerontology 26(1): 1. 10.1007/s10522-024-10146-2 39441393

[374] Yang, Qijiang , Xiaoyun Wu , Jinlan Duan , Yiyin Chen , and Tianrui Yang . 2025. “Inflammatory Parameters Mediates the Relationship Between Dietary Index for Gut Microbiota and Frailty in Middle‐aged and Older Adults in the United States: Findings From a Large‐scale Population‐Based Study.” Frontiers in Nutrition 12: 1553467. 10.3389/fnut.2025.1553467 40308632 PMC12040679

[375] Swoboda, Jessica R. , H Asuman Kiyak , Richard Darveau , and G Rutger Persson . 2008. “Correlates of Periodontal Decline and Biologic Markers in Older Adults.” Journal of Periodontology 79: 1920–1926. 10.1902/jop.2008.080005 18834247

[376] Cavagnino, Andrea , Olivier Gouin , Lionel Breton , and Martin Baraibar . 2026. “An Ex Vivo ‘Leaky Skin’ Model to Study Early Events Induced by Staphylococcus Aureus Protease.” Microorganisms 14: 1244. 10.3390/microorganisms14061244 42354869 PMC13304451

[377] Fang, Jiaqing , Xiaobo He , and Junjun Zhou . 2026. “Multi‐Site Microbiota Crosstalk in the Postmenopausal: From Dysbiosis Mechanisms to Precision Interventions.” Frontiers in Microbiology 17: 1702700. 10.3389/fmicb.2026.1702700 41800413 PMC12960634

[378] Quince, Christopher , Alan W. Walker , Jared T. Simpson , Nicholas J. Loman , and Nicola Segata . 2017. “Shotgun Metagenomics, From Sampling to Analysis.” Nature Biotechnology 35: 833–844. 10.1038/nbt.3935 28898207

[379] Callahan, Benjamin J. , Paul J. McMurdie , Michael J. Rosen , Andrew W. Han , Amy Jo A. Johnson , and Susan P. Holmes . 2016. “DADA2: High‐Resolution Sample Inference From Illumina Amplicon Data.” Nature Methods 13: 581–583. 10.1038/nmeth.3869 27214047 PMC4927377

[380] Chen, Liang , Na Zhao , Jiabao Cao , Xiaolin Liu , Jiayue Xu , Yue Ma , Ying Yu , et al. 2022. “Short‐ and Long‐read Metagenomics Expand Individualized Structural Variations in Gut Microbiomes.” Nature Communications 13: 3175. 10.1038/s41467-022-30857-9 PMC9177567 35676264

[381] Beghini, Francesco , Lauren J. McIver , Aitor Blanco‐Míguez , Leonard Dubois , Francesco Asnicar , Sagun Maharjan , Ana Mailyan , et al. 2021. “Integrating Taxonomic, Functional, and Strain‐level Profiling of Diverse Microbial Communities With bioBakery 3.” eLife 10: e65088. 10.7554/eLife.65088 33944776 PMC8096432

[382] Douglas, Gavin M. , Vincent J. Maffei , Jesse R. Zaneveld , Svetlana N. Yurgel , James R. Brown , Christopher M. Taylor , Curtis Huttenhower , and Morgan G. I. Langille . 2020. “PICRUSt2 for Prediction of Metagenome Functions.” Nature Biotechnology 38: 685–688. 10.1038/s41587-020-0548-6 PMC7365738 32483366

[383] Ma, Siyuan , Dmitry Shungin , Himel Mallick , Melanie Schirmer , Long H. Nguyen , Raivo Kolde , Eric Franzosa , et al. 2022. “Population Structure Discovery in Meta‐Analyzed Microbial Communities and Inflammatory Bowel Disease Using MMUPHin.” Genome Biology 23: 208. 10.1186/s13059-022-02753-4 36192803 PMC9531436

[384] Ling, Wodan , Jiuyao Lu , Ni Zhao , Anju Lulla , Anna M. Plantinga , Weijia Fu , Angela Zhang , et al. 2022. “Batch Effects Removal for Microbiome Data Via Conditional Quantile Regression.” Nature Communications 13: 5418. 10.1038/s41467-022-33071-9 PMC9477887 36109499

[385] Sun, Shan , Huijun Wang , Matthew Cb Tsilimigras , Annie Green Howard , Wei Sha , Jiguo Zhang , Chang Su , et al. 2020. “Does Geographical Variation Confound The Relationship Between Host Factors and the Human Gut Microbiota: A Population‐Based Study in China.” BMJ Open 10: e038163. 10.1136/bmjopen-2020-038163 PMC7678355 33444181

[386] Almeida, Alexandre , Stephen Nayfach , Miguel Boland , Francesco Strozzi , Martin Beracochea , Zhou Jason Shi , Katherine S. Pollard , et al. 2021. “A Unified Catalog of 204,938 Reference Genomes From the Human Gut Microbiome.” Nature Biotechnology 39: 105–114. 10.1038/s41587-020-0603-3 PMC7801254 32690973

[387] Coelho, Luis Pedro , Renato Alves , Álvaro Rodríguez del Río , Pernille Neve Myers , Carlos P. Cantalapiedra , Joaquín Giner‐Lamia , Thomas Sebastian Schmidt , et al. 2022. “Towards the Biogeography of Prokaryotic Genes.” Nature 601: 252–256. 10.1038/s41586-021-04233-4 34912116 PMC7613196

[388] Partridge, Sally R. , Stephen M. Kwong , Neville Firth , and Slade O. Jensen . 2018. “Mobile Genetic Elements Associated With Antimicrobial Resistance.” Clinical Microbiology Reviews 31: e00088−17. 10.1128/cmr.00088-17 30068738 PMC6148190

[389] Blanco‐Míguez, Aitor , Francesco Beghini , Fabio Cumbo , Lauren J. McIver , Kelsey N. Thompson , Moreno Zolfo , Paolo Manghi , et al. 2023. “Extending and Improving Metagenomic Taxonomic Profiling With Uncharacterized Species Using MetaPhlAn 4.” Nature Biotechnology 41: 1633–1644. 10.1038/s41587-023-01688-w PMC10635831 36823356

[390] Lloréns‐Rico, Verónica , Joshua A. Simcock , Geert R. B. Huys , and Jeroen Raes . 2022. “Single‐Cell Approaches in Human Microbiome Research.” Cell 185: 2725–2738. 10.1016/j.cell.2022.06.040 35868276

[391] Lagier, J.‐C. , F. Armougom , M. Million , P. Hugon , I. Pagnier , C. Robert , F. Bittar , et al. 2012. “Microbial Culturomics: Paradigm Shift in the Human Gut Microbiome Study.” Clinical Microbiology and Infection 18: 1185–1193. 10.1111/1469-0691.12023 23033984

[392] Lv, Bo‐Min , Yuan Quan , and Hong‐Yu Zhang . 2021. “Causal Inference in Microbiome Medicine: Principles and Applications.” Trends in Microbiology 29: 736–746. 10.1016/j.tim.2021.03.015 33895062

[393] Tian, Siyu , Xingyu Liao , Siqi Chen , Yu Wu , and Min Chen . 2024. “Genetic Association of the Gut Microbiota With Epigenetic Clocks Mediated by Inflammatory Cytokines: A Mendelian Randomization Analysis.” Frontiers in Immunology 15: 1339722. 10.3389/fimmu.2024.1339722 38903525 PMC11186987

[394] Kurilshikov, Alexander , Carolina Medina‐Gomez , Rodrigo Bacigalupe , Djawad Radjabzadeh , Jun Wang , Ayse Demirkan , Caroline I. Le Roy , et al. 2021. “Large‐Scale Association Analyses Identify Host Factors Influencing Human Gut Microbiome Composition.” Nature Genetics 53: 156–165. 10.1038/s41588-020-00763-1 33462485 PMC8515199

[395] Horvath, Steve. 2013. “DNA Methylation Age of Human Tissues and Cell Types.” Genome Biology 14: 3156. 10.1186/gb-2013-14-10-r115. PMC4015143 24138928

[396] Torma, Ferenc , Csaba Kerepesi , Mátyás Jókai , Gergely Babszki , Erika Koltai , Balázs Ligeti , Regina Kalcsevszki , et al. 2024. “Alterations of the Gut Microbiome Are Associated With Epigenetic Age Acceleration and Physical Fitness.” Aging Cell 23: e14101. 10.1111/acel.14101 38414315 PMC11019127

[397] Jans, Maude , and Lars Vereecke . 2025. “A Guide To Germ‐Free and Gnotobiotic Mouse Technology to Study Health and Disease.” The FEBS Journal 292: 1228–1251. 10.1111/febs.17124 38523409

[398] Bayer, Giuliano , Caroline M. Ganobis , Emma Allen‐Vercoe , and Dana J. Philpott . 2021. “Defined Gut Microbial Communities: Promising Tools to Understand and Combat Disease.” Microbes and Infection 23: 104816. 10.1016/j.micinf.2021.104816 33785422

[399] Lyko, Frank . 2018. “The DNA Methyltransferase Family: A Versatile Toolkit for Epigenetic Regulation.” Nature Reviews Genetics 19: 81–92. 10.1038/nrg.2017.80 29033456

[400] Kohli, Rahul M. , and Yi Zhang . 2013. “TET Enzymes, TDG and the Dynamics of DNA Demethylation.” Nature 502: 472–479. 10.1038/nature12750 24153300 PMC4046508

[401] Davletgildeeva, Anastasiia T. , and Nikita A. Kuznetsov . 2024. “The Role of DNMT Methyltransferases and TET Dioxygenases in the Maintenance of the DNA Methylation Level.” Biomolecules 14: 1117. 10.3390/biom14091117 39334883 PMC11430729

[402] Sommer, Felix , Joana P. Bernardes , Lena Best , Nina Sommer , Jacob Hamm , Berith Messner , Víctor A López‐Agudelo , et al. 2025. “Life‐Long Microbiome Rejuvenation Improves Intestinal Barrier Function and Inflammaging in Mice.” Microbiome 13: 91. 10.1186/s40168-025-02089-8 40176137 PMC11963433

[403] Böhnke, Jan R. 2016. “Book Review: Explanation in Causal Inference: Methods for Mediation and Interaction.” Quarterly Journal of Experimental Psychology 69: 1243–1244. 10.1080/17470218.2015.1115884 28190383

[404] Brown, Andrew J. , Susan M. Goldsworthy , Ashley A. Barnes , Michelle M. Eilert , Lili Tcheang , Dion Daniels , Alison I. Muir , et al. 2003. “The Orphan G Protein‐Coupled Receptors GPR41 and GPR43 are Activated by Propionate and Other Short Chain Carboxylic Acids.” Journal of Biological Chemistry 278: 11312–11319. 10.1074/jbc.M211609200 12496283

[405] Bravo, Javier A. , Paul Forsythe , Marianne V. Chew , Emily Escaravage , Hélène M Savignac , Timothy G. Dinan , John Bienenstock , and John F. Cryan . 2011. “Ingestion of *Lactobacillus strain* Regulates Emotional Behavior and Central GABA Receptor Expression in a Mouse via the Vagus Nerve.” Proceedings of the National Academy of Sciences 108: 16050–16055. 10.1073/pnas.1102999108 PMC3179073 21876150

[406] Bonaz, Bruno , Thomas Bazin , and Sonia Pellissier . 2018. “The Vagus Nerve At The Interface of the Microbiota‐Gut‐Brain Axis.” Frontiers in Neuroscience 12: 49. 10.3389/fnins.2018.00049 29467611 PMC5808284

[407] Pasolli, Edoardo , Lucas Schiffer , Paolo Manghi , Audrey Renson , Valerie Obenchain , Duy Tin Truong , Francesco Beghini , et al. 2017. “Accessible, Curated Metagenomic Data Through ExperimentHub.” Nature Methods 14: 1023–1024. 10.1038/nmeth.4468 29088129 PMC5862039

[408] Mitchell, Alex L. , Alexandre Almeida , Martin Beracochea , Miguel Boland , Josephine Burgin , Guy Cochrane , Michael R. Crusoe , et al. 2020. “MGnify: The Microbiome Analysis Resource in 2020.” Nucleic Acids Research 48: D570–D578. 10.1093/nar/gkz1035 31696235 PMC7145632

[409] Liu, Can , Xiaohan Wang , Zhilin Zhang , Wenboxin Wang , Tianci Wang , Yuchen Zhao , Mingyu Wang , and Wei‐Hua Chen . 2026. “GMrepo v3: A Curated Human Gut Microbiome Database With Expanded Disease Coverage and Enhanced Cross‐Dataset Biomarker Analysis.” Nucleic Acids Research 54: D734–D742. 10.1093/nar/gkaf1190 41277537 PMC12807640

[410] Proctor, Lita M. , Heather H. Creasy , Jennifer M. Fettweis , Jason Lloyd‐Price , Anup Mahurkar , Wenyu Zhou , Gregory A. Buck , et al., Integrative HMP (iHMP) Research Network Consortium . 2019. “The Integrative Human Microbiome Project.” Nature 569: 641–648. 10.1038/s41586-019-1238-8 31142853 PMC6784865

[411] Mirzayi, Chloe , Audrey Renson , Cesare Furlanello , Susanna‐Assunta Sansone , Fatima Zohra , Shaimaa Elsafoury , Ludwig Geistlinger , et al. 2021. “Reporting Guidelines for Human Microbiome Research: The STORMS Checklist.” Nature Medicine 27: 1885–1892. 10.1038/s41591-021-01552-x PMC9105086 34789871

[412] Yilmaz, Pelin , Renzo Kottmann , Dawn Field , Rob Knight , James R. Cole , Linda Amaral‐Zettler , Jack A. Gilbert , et al. 2011. “Minimum Information About a Marker Gene Sequence (MIMARKS) and Minimum Information About Any (x) Sequence (MIxS) Specifications.” Nature Biotechnology 29: 415–420. 10.1038/nbt.1823 PMC3367316 21552244

[413] Sinha, Rashmi , Christian C. Abnet , Owen White , Rob Knight , and Curtis Huttenhower . 2015. “The Microbiome Quality Control Project: Baseline Study Design and Future Directions.” Genome Biology 16: 276. 10.1186/s13059-015-0841-8 26653756 PMC4674991

[414] Wirbel, Jakob , Konrad Zych , Morgan Essex , Nicolai Karcher , Ece Kartal , Guillem Salazar , Peer Bork , Shinichi Sunagawa , and Georg Zeller . 2021. “Microbiome Meta‐Analysis and Cross‐Disease Comparison Enabled by the Siamcat Machine Learning Toolbox.” Genome Biology 22: 93. 10.1186/s13059-021-02306-1 33785070 PMC8008609

[415] Greathouse, K Leigh , and Ankan Choudhury . 2025. “Precision nutrition and the Gut Microbiome: Harnessing AI to Revolutionize Cancer Prevention and Therapy.” Cell Host & Microbe 33: 766–776. 10.1016/j.chom.2025.05.011 40505617

[416] Haran, John P. , Alden L. Zeamer , Doyle V. Ward , Protiva Dutta , Vanni Bucci , and Beth McCormick . 2021. “The Nursing Home Older Adult Gut Microbiome Composition Shows Time‐Dependent Dysbiosis and Is Influenced by Medication Exposures, Age, Environment, and Frailty.” Journals of Gerontology: Series A 76: 1930–1938. 10.1093/gerona/glab175 PMC8514073 34125200

[417] Jeffery, Ian B. , Denise B. Lynch , and Paul W. O'Toole . 2016. “Composition and Temporal Stability of the Gut Microbiota in Older Persons.” The ISME Journal 10: 170–182. 10.1038/ismej.2015.88 26090993 PMC4681863

[418] Zhou, Xin , Xiaotao Shen , Jethro S. Johnson , Daniel J. Spakowicz , Melissa Agnello , Wenyu Zhou , Monica Avina , et al. 2024. “Longitudinal Profiling of the Microbiome at Four Body Sites Reveals Core Stability and Individualized Dynamics During Health and Disease.” Cell Host & Microbe 32: 506–526.e9. 10.1016/j.chom.2024.02.012 38479397 PMC11022754

[419] Stennett, Christina A. , Michael France , Michelle Shardell , Sarah J. Robbins , Sarah E. Brown , Elizabeth D. Johnston , Katrina Mark , Jacques Ravel , and Rebecca M. Brotman . 2024. “Longitudinal Profiles of the Vaginal Microbiota of Pre‐, Peri‐, and Postmenopausal Women: Preliminary Insights From a Secondary Data Analysis.” Menopause 31: 537–545. 10.1097/gme.0000000000002358 38787353 PMC11886898

